# Identification of hypoxia- and mitophagy-related diagnostic biomarkers for ulcerative colitis based on bioinformatic analysis and machine learning

**DOI:** 10.1371/journal.pone.0339296

**Published:** 2026-01-21

**Authors:** Zewei Sheng, Lun Zhao, Yu Fu, Xuefeng Liu, Yuyu Peng, Yangling Huang, Yuhan Jian, Yanlin Zhu, Yuedong Liu

**Affiliations:** 1 Third Clinical College, Liaoning University of Traditional Chinese Medicine, Shenyang, Liaoning, China; 2 Third Affiliated Hospital, Liaoning University of Traditional Chinese Medicine, Shenyang, Liaoning, China; 3 College of Acupuncture–Moxibustion and Massage, Liaoning University of Traditional Chinese Medicine, Shenyang, Liaoning, China; 4 Technology Center, Liaoning University of Traditional Chinese Medicine, Shenyang, Liaoning, China; University of Helsinki: Helsingin Yliopisto, FINLAND

## Abstract

**Background:**

Ulcerative colitis (UC) is a chronic nonspecific inflammatory bowel disease of unknown etiology that is associated with a significant risk of progression to colorectal cancer. The aim of this study was to systematically identify hypoxia- and mitophagy-related molecular signatures associated with UC, thereby providing novel insights into disease mechanisms and therapeutic strategies.

**Methods:**

A comprehensive analytical framework integrating differential expression analysis and functional enrichment assessment was employed to systematically characterize dysregulated mitophagy-related genes (MRGs) and hypoxia-related genes (HRGs) in UC and their associated pathogenic pathways. We employed two advanced machine learning methods, support vector machine with recursive feature elimination (SVM-RFE) and least absolute shrinkage and selection operator (LASSO), to evaluate diagnostic models validated by receiver operating characteristic (ROC) curves and optimize feature selection. These results were verified by basic experiments. We subsequently analyzed immune cell infiltration to clarify the interaction between mitophagy/hypoxia and immunological disorders in UC pathogenesis. Finally, mRNA–transcription factor (TF) and mRNA–miRNA regulatory networks were constructed, revealing intricate molecular crosstalk among hub genes through systematic bioinformatic analyzes.

**Results:**

After validation with two machine learning approaches, two pivotal biomarkers (*CD55* and *CPT1A*) with diagnostic potential were rigorously selected. ROC curve analysis revealed the superior diagnostic efficacy of these key genes, confirming their clinical discriminative capacity. Experimental verification confirmed these findings. Notably, subsequent immune profiling revealed significant upregulation of multiple immune cell populations in the high-risk UC subgroup. Furthermore, the expression of diagnostic biomarkers was significantly correlated with dynamic changes in immune cell infiltration, suggesting that these biomarkers play immunomodulatory roles in UC progression. Finally, mRNA–miRNA and mRNA–TF regulatory network analyzes revealed complex interactions.

**Conclusions:**

We elucidated the relationship between UC and hypoxia/mitophagy and identified potential diagnostic biomarkers. This study provides a reference for the future development of targeted treatment strategies to improve diagnostic and therapeutic protocols for UC.

## Introduction

Ulcerative colitis (UC), the most common type of inflammatory bowel disease (IBD), is characterized by chronic nonspecific inflammation and ulceration of the colonic mucosa, often leading to abdominal pain and bloody diarrhea. Currently, the primary therapeutic approaches for UC include nonsteroidal anti-inflammatory drugs (NSAIDs), steroid hormones, immunosuppressive agents, and surgical interventions. However, these treatment modalities present limitations [1]. As urbanization progresses and as environmental factors and dietary practices shift, the annual incidence of UC continues to increase [2]. Furthermore, as a precursor lesion for colorectal cancer, UC not only causes significant morbidity—manifested as symptoms such as diarrhea and hematochezia—but also imposes a substantial burden on patients’ families and society as a whole [3,4]. The prevailing perspective suggests that the development of UC is influenced by the interplay of genetic predispositions and environmental elements, with more than 200 genetic loci associated with the occurrence of UC. Environmental factors predispose individuals to UC by influencing the diversity and structure of the trillions of bacteria, viruses, and fungi that constitute the gut microbiota [4,5]. However, the large number of genetic loci, hundreds of millions of gut microbes, and unpredictable environmental factors have prevented a full understanding of the pathogenesis of UC. Therefore, exploring the underlying pathological mechanism of UC is important for improving the diagnosis and treatment of this disease.

Mitochondria are required to maintain intestinal homeostasis because of the chronic “physiological” “resource-poor” state of the intestine [6,7]. Under hypoxic conditions, mitochondria produce large amounts of reactive oxygen species (ROS) to maintain intestinal homeostasis. However, when excessive amounts of ROS are produced, they can damage cellular structure and function [7,8]. The body selectively removes damaged mitochondria through lysosomal degradation, a process known as mitophagy, to halt this vicious cycle [9]. Emerging evidence suggests that in UC and Crohn’s disease pathogenesis, increased ROS generation concomitant with compromised antioxidant defense disrupts homeostasis, which manifests clinically through proinflammatory cytokine cascades, compromised epithelial tight junction integrity, and sustained oxidative tissue injury [10]. In addition, mitophagy plays a dual role in UC; excessive mitophagy leads to cellular energy depletion and induces apoptosis, whereas the excessive inhibition of mitophagy hinders the self-clearance of damaged mitochondria, accelerates ROS production and exacerbates inflammatory responses [11–13]. Systematic investigations identifying hypoxia–mitophagy axis-associated genetic signatures and their mechanistic contributions to UC pathology are lacking. The objective of this research is to fill the existing gaps via a comprehensive analysis of DEGs associated with hypoxia and mitophagy. This analysis will facilitate a clearer understanding of their potential utility as diagnostic biomarkers in the management of UC.

## Materials and methods

### Data acquisition

We utilized the R package GEO query [14] to obtain the UC datasets GSE75214 [15] and GSE179285 [16] from the GEO database [17], which is accessible at https://www.ncbi.nlm.nih.gov/geo/. The samples in both GSE75214 and GSE179285 were derived from colonic tissues from *Homo sapiens*. The chip platform for the GSE75214 dataset is GPL6244, whereas the GPL6480 platform is used for the GSE179285 dataset; see S1 Table for details. In the GSE75214 dataset, 74 cases of UC_colon_active were used as UC samples, and 11 cases were used as control samples. The GSE179285 dataset comprises 23 cases of UC and 23 control samples representing nonactive sigmoid colon tissues.

We utilized the GeneCards database (https://www.genecards.org/) to identify mitochondria-related genes (MRGs) [18]. By employing the keyword “mitophagy” in the search and subsequently filtering for MRGs that are classified as “protein coding” with a relevance score exceeding 2, we identified a total of 1680 MRGs, whose detailed information is provided in S4 Table. In a similar manner, we identified 2360 hypoxia-related genes (HRGs), with detailed information provided in S5 Table.

### Analysis of differentially expressed genes

The R package sva [19] was applied to remove batch effects from GSE75214 and GSE179285, resulting in a merged GEO dataset (the training set). Among them, the combined datasets contained 97 UC samples and 34 control samples. Finally, the R package limma [20] was applied to normalize the merged GEO dataset. Principal component analysis (PCA) [21] and relative log expression (RLE) analysis were performed on the gene expression data before and after batch effect removal to evaluate the effectiveness of the process.

To identify robust differentially expressed genes (DEGs) associated with UC, we employed a complementary analytical strategy to mitigate potential systematic biases introduced by any single method. Differential expression analysis between the UC and control groups was first performed on the batch-corrected combined datasets using the R package limma (version 3.58.1). Genes with a |logFC| > 0.5 and a p value < 0.05 were considered DEGs for subsequent analysis. Afterward, to enhance the robustness and statistical efficacy of the results, with reference to previous studies [22], we used two well-established methods of differential expression analysis, namely, RobustRankAggreg (RRA) and DExMA, as a supplementary analysis. The RRA package was used to integrate gene ranking lists from the separate analyzes of the original GSE75214 and GSE179285 datasets, prioritizing genes that consistently ranked high across both independent datasets. The DExMA package, specifically its metaAnalysisDE function, provided a full meta-analysis pipeline based on standardized expression data, in which DEGs were identified using Fisher’s method. The high-confidence gene sets derived from both RRA ranking and DExMA analysis were then intersected with the DEGs obtained from the primary limma analysis of the integrated dataset. This intersection was visualized using a Venn diagram.

To investigate the specific roles of mitophagy and hypoxia in UC, we focused on the DEGs identified from the primary limma analysis of the integrated training set. From this complete set of limma-derived DEGs (|logFC| > 0.5, p value < 0.05), we selected those that were also present among the predefined mitophagy-related genes (MRGs) and hypoxia-related genes (HRGs). Intersections were taken and plotted as Wayne plots, and heatmaps were drawn for presentation via the R package pheatmap (Version 1.0.12). The expression of differentially expressed genes filtered from the MAHRDEG list is displayed, and all genes are sorted in logFC descending order. In the clustering analysis, both the gene and sample dimensions were hierarchically clustered using Euclidean distance.

Notably, in this study, we prioritized the selection of the publicly available microarray datasets GSE75214 and GSE179285 from the GEO database based on the following criteria: relevance to UC, consistency in sampling site and species, completeness of information, and a sufficiently large sample size. Currently, the number of publicly available RNA-seq datasets for UC remains limited, and their sample sizes are generally insufficient to meet the requirements of a rigorous machine learning analysis. In contrast, microarray datasets allow for more effective batch effect correction and integration, thereby ensuring greater stability and reliability of the analysis. Furthermore, we employed an unsupervised batch correction approach to mitigate technical batch effects between the two datasets while preventing potential data leakage. The ComBat algorithm from the sva package was applied to GSE75214 and GSE179285 using only the dataset origin as the batch covariate, without utilizing any sample class labels (control or ulcerative colitis). The aim of this process was to align the overall distribution of the two datasets. The resulting batch-corrected data were merged to form the training set (Combined datasets). For validation, we used the raw, uncorrected GSE179285 dataset as an independent validation set. This dataset was only used for the final evaluation of the diagnostic performance and generalization capabilities of the model. The validation set remained “unseen” until the model was constructed.

### Gene Ontology (GO) and pathway (KEGG) enrichment analyzes

In this study, GO and KEGG functional enrichment analyzes of genes associated with mitophagy and hypoxia were performed using the R package clusterProfiler (version 4.10.0). The FDR (q-value) threshold was set at <0.25 during the initial screening stage, referencing the practice of some exploratory transcriptome studies that have used looser thresholds to preserve potential biological signals in high-dimensional data while testing multiple hypotheses [23–25].

### Gene set enrichment analysis (GSEA)

GSEA was performed on the comprehensive gene set from the integrated GEO datasets using the R package clusterProfiler (version 4.10.0). The GSEA parameters were set as follows: a seed value of 2020 was used, and the analysis was run for a total of 1000 computations. The gene sets were limited to include between 10 and 500 genes per set. From the Molecular Signatures Database (MSigDB) [26], we acquired the C2 gene set (v2023.2) and performed GSEA using human gene symbols (*Homo sapiens*, Hs). The Benjamini–Hochberg (BH) method was used for p value correction, and the FDR was controlled at 0.25.

### Establishment of a diagnostic model for ulcerative colitis

A logistic regression model was constructed using the MHRDEGs from the combined datasets to develop a diagnostic model for UC. When the outcome variable was a categorical variable, particularly for differentiating between the UC and control groups, logistic regression was applied to assess the association between the independent variables and the categorical outcome. When the p value was < 0.05, the MHRDEGs were selected, and a logistic regression model was constructed. A forest plot of the model was constructed.

MHRDEGs were subsequently analyzed using the R package e1071 (Version 1.7–14). The SVM-RFE algorithm [27] was employed to identify candidate biomarkers. In applying the SVM-RFE algorithm, 5-fold cross-validation (5-fold cross-validation) was used to ensure the reproducibility and stability of the results.

Afterward, a LASSO analysis of the MHRDEGs screened by the SVM-RFE algorithm was performed using the R package glmnet [28] (version 4.1–8).

The LASSO regression analysis, which used a seed value of 500 and a family designation of “binomial”, was built on the foundation of the linear regression analysis. A regularization term was incorporated into the analysis to reduce the risk of overfitting and improve the generalization of the model. In constructing the LASSO regression model, the optimal regularization parameter λ is determined through 10-fold cross-validation. This process calculates the model deviation for a range of λ values, identifying the λ that minimizes the cross-validation error (lambda.min) and the λ within one standard error of this minimum (lambda.1 se). To achieve a more concise model with enhanced generalizability, the model associated with lambda.1 se was chosen, adhering to the “one standard error” principle, which ultimately identified key genes and effectively mitigated the risk of overfitting. This term was defined as the product of lambda and the absolute value of the coefficient. The outcomes derived from the LASSO regression analysis are illustrated in both a diagnostic model diagram and a variable trajectory diagram. The findings from the LASSO regression analysis were used to construct a diagnostic model for UC. LASSO regression analysis was employed to construct a diagnostic model for UC that incorporated genes associated with mitophagy and hypoxia, which were identified as key genes.

In addition, we employed two complementary machine learning feature selection methods: support vector machine recursive feature elimination (SVM-RFE) and least absolute shrinkage with selection operator (LASSO). SVM-RFE is based on support vector classification, which emphasizes the contribution of each feature’s ranking in high-dimensional data, whereas LASSO regression uses L1 regularization for variable filtering and model sparsification to reduce the risk of overfitting. The combination of these two methods has been widely used to improve the robustness of feature screening [29–31]. All feature selection (including SVM-RFE and LASSO) and model training steps used only the data and labels from the training set (combined datasets). The performance of the final model was evaluated on the validation set (original GSE179285).

The LASSO risk score was derived from the risk coefficients obtained through LASSO regression analysis. The risk score was computed using the following formula:


$$RiskScore=\sum_{\dot{l}}Cofficient\left({gene}_{\dot{l}}\right)*mRNAExpressiont\left({gene}_{\dot{l}}\right)$$

### Validation of the diagnostic model for ulcerative colitis

The R package rms (version 6.7−1) was used to construct a diagram based on the logistic regression results, which revealed the relationships among key genes. A calibration curve was constructed through a calibration analysis based on the results of the LASSO regression analysis. The R package ggDCA (version 1.1) was used to generate decision curve analysis (DCA) maps based on key genes from the combined GEO datasets [32].

The UC cohort was divided into high-risk and low-risk groups based on the median risk score from the UC diagnostic model. A comparative analysis was conducted to further investigate the discrepancies in the expression of key genes between the high-risk and low-risk groups of UC patients, resulting in a graphical representation of the expression levels of these key genes.

Afterward, the R package GOSemSim [33] (version 2.28.0) was used to calculate the functional correlations of key genes, and the functional correlations between key genes were analyzed by functional similarity. Finally, the R package RCircos (version 1.2.2) [34] was used to determine the chromosomal location of the key genes.

### Verification of the differential expression of key genes

Charts of the comparative analysis were generated using the expression levels of these key genes to better clarify the differential expression of key genes between the UC group and the control group within the combined GEO datasets and GSE179285. The R package pROC (version 1.18.5) was used to construct ROC curves for the key genes and calculate their corresponding AUC values.

The Spearman correlation coefficient was calculated to analyze the associations between the expression levels of the key genes in the combined GEO datasets and GSE179285 to explore the relationships among the key genes. The findings of the correlation analysis were visualized using the R package pheatmap. The correlation coefficients were categorized as follows: an absolute value less than 0.3 indicated a weak or no correlation, values from 0.3 to 0.5 indicated a low correlation, coefficients from 0.5 to 0.8 indicated a moderate correlation, and coefficients above 0.8 indicated a strong correlation.

### Analysis of immune cell infiltration associated with key genes in the high- and low-risk groups using the ssGSEA algorithm

The ssGSEA method was used to evaluate the proportions of different immune cell types present in each sample [35]. Initially, various types of infiltrating immune cells, including but not limited to activated CD8^+^ T cells, activated dendritic cells, gamma-delta T cells, natural killer cells, and several subtypes of human immune cells, such as regulatory T cells, were identified and categorized. Next, the R package ggplot2 (version 3.4.4) was used to create comparative visualizations, illustrating the differences in the levels of immune cells in UC samples between the low-risk and high-risk cohorts from the combined GEO datasets. Immune cells whose abundance significantly differed between the two groups were subsequently selected for in-depth analysis. The relationships among immune cells were evaluated using the Spearman correlation method, and heatmaps were constructed. Afterward, the correlations between key genes and immune cells were evaluated using the Spearman algorithm, and the R package ggplot2 (version 3.4.4) was used to generate a correlation bubble chart.

### Analysis of the mRNA–TF and mRNA–miRNA regulatory networks of key genes

Using the ChIPBase database [36] (http://rna.sysu.edu.cn/chipbase/), we identified TFs and analyzed their regulatory effects on key genes. Subsequently, Cytoscape [37] was used to construct and visualize the mRNA–TF regulatory network.

The key genes and their relationships with miRNAs were subsequently analyzed using the StarBase v3.0 database [38] (https://starbase.sysu.edu.cn/) and, and the mRNA–miRNA regulatory network was visualized using Cytoscape software.

### Experimental validation of key genes

#### Animal modeling and grouping.

Twelve male specific pathogen-free (SPF)-grade rats, weighing 220 g ± 10 g (Certificate of Conformity No. SCXK (Liao) 2020−0001), were provided by Liaoning Changsheng Biotechnology Co. Prior to the experiments, the rats were acclimated for 1 week; all of them were fed and watered ad libitum, and they were housed in a quiet environment (3 rats in 1 cage) and maintained on a light–dark cycle for 12 hours at a room temperature of 22°C.

When experimental animal research is being conducted, the NC3R guidelines on humane endpoints must be followed. Humane endpoints were established to ensure animal welfare. Criteria for humane endpoints included severe weight loss exceeding 15% of the baseline weight, pain that was ineffectively relieved by analgesics, an inability to eat or drink, lethargy, weakness, dehydration, obvious and severe pain, and abnormal behaviors, such as difficulty breathing and impaired movement. Euthanasia was performed as soon as the animal met the criteria for humane endpoints, and the time before euthanasia was minimized to minimize suffering. In the present study, we monitored all the animals closely, and no animals died before the established euthanasia criteria were met.

The UC model was established as described in a previous study [39]. Briefly, the animals were fasted for 24 hours, and water was freely available. In a blinded manner, the rats were randomly divided into blank and model groups of 6 rats each. The rats in the UC group were anesthetized with an intraperitoneal injection of 20% urethane (0.5 ml/100 g); then, a disposable rubber catheter was inserted approximately 8 cm into the anus of the rats, and TNBS (40 mg/kg, in 50% ethanol) was slowly injected. The head was kept down for 5 min to ensure that the TNBS remained in the colon. After modeling, both groups of rats were fed and watered ad libitum, and the health and behavioral status of all the rats were monitored. TNBS was administered for 7 d, and on day 8, the rats in the 2 groups were sacrificed, and the colon tissues were removed.

At the end of the experiment, we administered an intraperitoneal injection of urethane solution for euthanasia by an anesthesia overdose. After the injection, the rats were observed for 10–15 min to confirm their death, which was assessed by the disappearance of respiration, cardiac arrest and pupil dilation. After the rats were confirmed to be dead, the colon tissue was removed. Biological materials and animal carcasses were disposed of in accordance with the Laboratory Animal Center Hazardous Waste Management Program to ensure compliance with biohazard safety standards and to maintain animal welfare.

### Ethics statement

All animal experimental protocols were performed in accordance with relevant guidelines and regulations, including the ARRIVE guidelines and the guidelines of the Animal Ethics Committee of the Second Affiliated Hospital of Liaoning University of Traditional Chinese Medicine. The animal experiments were approved by the Experimental Animal Ethics Committee of the Second Hospital of Liaoning University of Traditional Chinese Medicine (Approval No. LZYY250303). Optimal animal welfare conditions were ensured at all stages of our study. The rats were monitored daily to assess their general health.

### Hematoxylin and eosin (H&E) staining

The tissue sections were sequentially incubated with xylene I for 15 min, xylene II for 15 min, xylene III for 15 min, anhydrous ethanol I for 5 min, and anhydrous ethanol II for 5–75% alcohol for 5 min and then were washed with tap water. Afterward, the sections were stained with a hematoxylin staining solution for 3–5 min, washed with tap water, differentiated in differentiation solution, washed with tap water, returned to blue in return blue solution, and rinsed with running water. Afterward, the sections were sequentially dehydrated in a gradient of 85% and 95% alcohol for 5 min each and stained with the eosin staining solution for 5 min. Finally, the sections were sequentially incubated with anhydrous ethanol I for 5 min, anhydrous ethanol II for 5 min, anhydrous ethanol III for 5 min, xylene I for 5 min, and xylene II for 5 min for transparency and sealed with neutral gum. Images were acquired for analysis. An H&E kit (Cat: No. G1120-3; Solarbio, Ltd.), neutral gum (Cat: No. 10004160; Sinopharm Chemical Reagent Co., Ltd.), and image acquisition software (TissueGnostics, AUT) were used.

### Real-time qPCR assay

Total RNA was extracted from colon tissues using the total RNA extraction reagent RNAiso plus (cat. no. 9108; Takara, Ltd.), the concentration of RNA was subsequently determined using a NanoDrop 2000c spectrophotometer (Thermo Fisher Scientific, USA), and its purity was determined by calculating the A260/A280 ratio. Afterward, reverse transcription was performed using the PrimeScript RT Master Mix kit (cat. no. RR036A; Takara, Ltd) in strict compliance with the protocol provided by the manufacturer to generate cDNA. Real-time fluorescence quantitative PCR was subsequently performed, and a 20 µl PCR system was constructed as follows: (1) 0.5 µl of forward primer (10 µM); (2) 0.5 µl of reverse primer (10 µM); (3) 0.5 µl of cDNA; (4) 10 µl of TB Green Premix Ex Taq II (cat. no. RR820A; Takara, Ltd.); and (5) 10 µl of nuclease-free water (cat. no. 9012 Takara, Ltd.). All the reagents were mixed well, added to a 96-well PCR plate, sealed and centrifuged. The reaction was performed on a PCR instrument (Analytik Jena, DEU) with the following temperature settings: (1) 50°C for 2 min, (2) 95°C for 2 min, (3) 95°C for 15 s, and (4) 60°C for 40 s. Steps (3) and (4) were performed for 40 cycles, and changes in the expression of the target genes were calculated statistically using the 2-ΔΔCt method. β-Actin was used as an internal reference, and the primer sequences are shown in Table 1.

**Table 1:** Primer sequences.

Primer name	Primer sequence
*CD55* F	AGTAGTGCACCACCCAAGTG
*CD55* R	TGTGAGACGTTGGTTTGACTCT
*CPT1A* F	AGGTCTGGCTCTACCACGAT
*CPT1A* R	CGCATCCAGGGACTGCTTAT
*β-actin* F	CTGTGTGGATTGGTGGCTCT
*β-actin* R	AGCTCAGTAACAGTCCGCCT

### Western blotting

Colon tissues were lysed using RIPA lysis solution (Cat. No. P0013B; Beyotime, Ltd.) supplemented with a serine protease inhibitor (PMSF) (Cat. No. ST2573−5 g; Beyotime, Ltd.), after which the samples were placed on ice. The protein concentration was determined using a BCA protein concentration assay kit (Cat. No. P0010S; Beyotime, Ltd.). Subsequently, aliquots of lysates containing 20 μg of protein were treated with protein sampling buffer (Cat. No. P0015A, Beyotime, Ltd) and PBS (Cat. No. G4202, Servicebio, Ltd), and the samples were subsequently boiled for 5 min and stored at −20°C. Equal amounts of total protein (20 μg/lane) extracted from the different samples were separated on a 10% SDS–PAGE gel at 120 V for 90 min and transferred to a 0.45 μm polyvinylidene difluoride membrane (cat. no. IPVH00010; MERCK Millipore) for 70 min at 70 V. Subsequently, the membranes were blocked with 5% skim milk powder in TBS (TBST) containing 0.05% Tween-20 for 1 h at room temperature. The PVDF membrane was subsequently washed with TBST 3 times for 10 min each. The specific primary antibody was subsequently incubated with the membrane overnight at 4°C. The next day, the PVDF membranes were washed 3 times with TBST for 10 min each, followed by incubation with goat anti-rabbit IgG H&L (HRP) (1:20,000; Cat. No. ab6721; Abcam). Each PVDF membrane was exposed to an enhanced chemiluminescence kit (ECL reagent kit) (Cat. No. BL523B; Biosharp, Ltd.). The antibodies used were β-actin (1:100000; Cat. No. AC026; ABclonal, Ltd.), *CD55* (1:1000; Cat. No. AF5259; Affinity, Ltd), and *CPT1A* (1:1000; Cat. No. DF12004; Affinity, Ltd.). Finally, the images were analyzed using ImageJ software (version 2.14.0; National Institutes of Health).

### Immunohistochemical staining

The dehydration, embedding, and sectioning steps were the same as those for H&E staining. First, the antigen retrieval solution was placed into a heat-resistant container and microwaved to boiling. The section rack was placed into the antigen retrieval solution and continuously heated at low heat for 10 min, naturally cooled to room temperature, and rinsed by immersion in PBS for 5 min; this process was repeated 3 times. The samples were subsequently incubated with 3% H^2^O^2^ at room temperature for 15 min and rinsed by immersion in PBS for 5 min; this process was repeated 3 times. BSA was added dropwise and incubated for 15 min at room temperature, after which primary antibody working solution was added dropwise and incubated overnight at 4°C in a humid chamber. After an overnight incubation, the samples were immersed in PBS for 5 min, which was repeated 3 times. Afterward, the secondary antibody working solution was added and incubated for 30 min at 37°C in a humid chamber, which was followed by immersion in PBS for 5 min for washing, and this process was repeated 3 times. One hundred microliters of color-developing reagent was added dropwise, and the reaction was quickly terminated in water when the color was just darkened. The slices were immersed in hematoxylin for 3 min and then rinsed with running tap water for 2 min. The slices were immersed in 1% hydrochloric acid in ethanol for 3 s and then immediately rinsed with running tap water for 20 min to return the color to blue. Finally, the slices were blocked with neutral dendrimers. A Metal-Enhanced DAB Substrate Kit (Cat. No. DA1016, Solarbio, Ltd.) was used. Image acquisition was carried out using image acquisition software (TissueGnostics, AUT). Finally, the average optical density value (AOD) was measured using image analysis software (Image-Pro Plus) for quantitative analysis. The formula is as follows: Integrated Optical Density (IOD)/Area-SUM = Average optical density value (AOD).

### Statistical analysis

The data processing and analysis procedures described in this study were performed using R software (version 4.2.2). The statistical significance of the differences in normally distributed continuous variables between two groups was evaluated using the independent Student’s t test, unless otherwise specified. The Mann–Whitney U test, also known as the Wilcoxon rank sum test, was used to assess differences among variables that did not follow a normal distribution. The Kruskal–Wallis test was employed for comparative analyzes involving three or more groups. Furthermore, Spearman’s correlation analysis was performed to determine the correlations between various molecules. All statistical p values were two-tailed and considered significant at p < 0.05, unless otherwise specified (Fig 1).

## Results

### Technology roadmap

**Fig 1:**
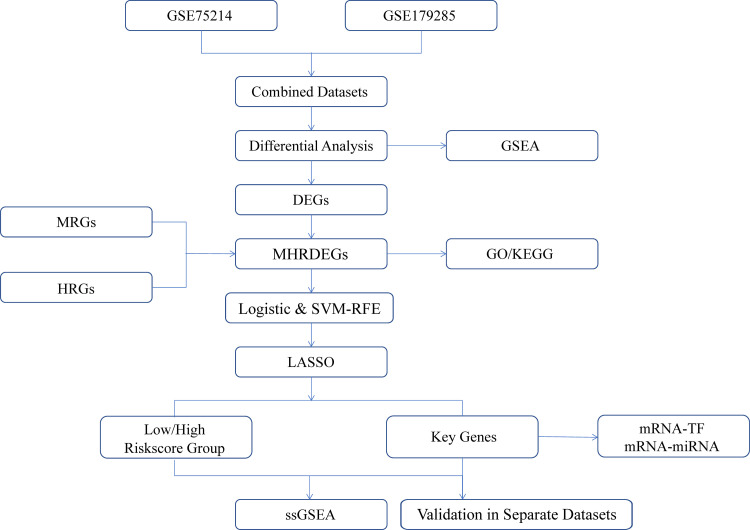
Technology roadmap. DEGs, differentially expressed genes; MHRDEGs, mitophagy and hypoxia-related differentially expressed genes; MRGs, mitophagy-related genes; HRGs, hypoxia-related genes; GSEA, gene set enrichment analysis; GO, Gene Ontology; KEGG, Kyoto Encyclopedia of Genes and Genomes; LASSO, least absolute shrinkage and selection operator; TF, transcription factor; ssGSEA, single-sample gene set enrichment analysis.

### Merging the ulcerative colitis datasets

**Fig 2:**
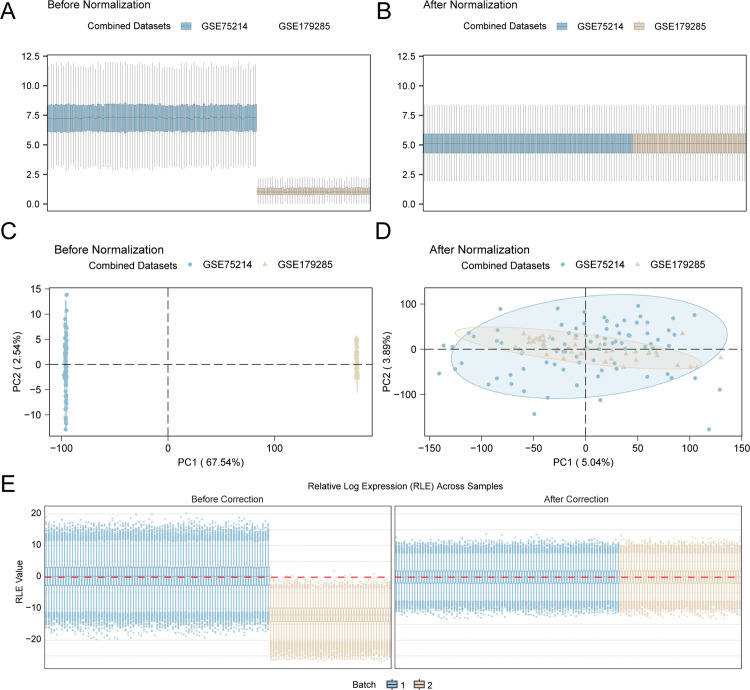
Batch effect removal. A. Prior to batch effect removal, the distribution of GEO datasets was visualized in a box plot. B. Box plots showing the distribution of GEO datasets after batch correction. C. PCA plot showing the distribution of low-dimensional features within the datasets before the elimination of batch effects. D. PCA plot of the GEO datasets after batch correction. In this plot, the UC dataset GSE179285 is presented in light yellow, and the UC dataset GSE75214 is shown in light blue. GEO datasets: combined datasets. E. RLE plot of the combined datasets after debatch processing; UC: ulcerative colitis; PCA: principal component analysis.

First, the R package sva was employed to eliminate the batch effects present in the UC datasets GSE75214 and GSE179285, resulting in the creation of combined GEO datasets. Following this step, boxplots were constructed to assess the expression levels derived from the integrated GEO datasets both before and after the elimination of the batch effect, as displayed in Figs 2A and 2B. A principal component analysis (PCA) plot (Fig 2C–D) was generated to evaluate the distribution of low-dimensional features in the dataset before and after batch effect removal. RLE plots (Fig 2E) were complemented with PCA to quantitatively demonstrate sample expression consistency. The results from the distribution boxplot, the PCA plot and the RLE plot suggested that batch effects across UC samples were effectively minimized after removing batch influences.

### Differentially expressed genes related to mitophagy and hypoxia in UC

**Fig 3:**
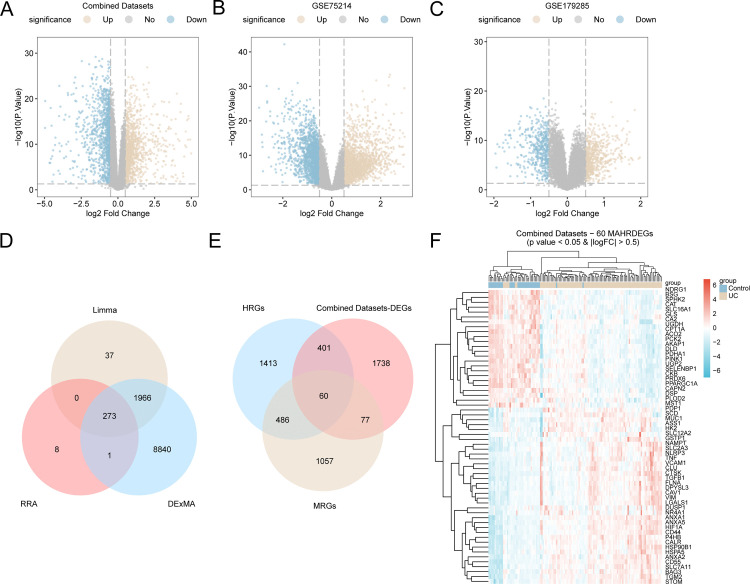
Differential gene expression analysis. A–C. Volcano plots illustrating the results of the differential gene expression analysis between the UC group and control group in the combined GEO datasets (A), GSE179285 (B) and GSE75214 (C) were generated. D. Venn diagram of the overlapping genes from the combined datasets, the RRA-sorted high-ranking genes, and the DExMA-identified high-ranking genes. E. Venn diagram of MRGs and HRGs identified among the DEGs in all ulcerative colitis samples from the integrated GEO datasets. D-E. Heatmap of mitochondrial autophagy- and hypoxia-associated differentially expressed genes in the combined datasets. DEGs, differentially expressed genes; MRGs, mitophagy-related genes; HRGs, hypoxia-related genes. The UC group is presented in pale yellow, and the control group is presented in pale blue. In the heatmap, high expression is shown in red, while low expression is shown in blue.

The R package limma was used to analyze DEGs in the combined datasets, GSE179285 datasets and GSE75214 datasets, with the goal of identifying DEGs across both datasets. The analysis yielded a total of 2276 DEGs from the combined datasets that satisfied the criteria of |logFC| > 0.5 and a p value < 0.05. Using these thresholds, a total of 1196 genes were upregulated (logFC > 0.5 and p value < 0.05), whereas 1080 genes were downregulated (logFC < −0.5 and p value < 0.05). On the basis of the differential expression results for this dataset, a volcano map was generated (Fig 3A). The GSE75214 dataset included a total of 3739 DEGs whose |logFC| > 0.5, p value < 0.05, and downregulated genes (logFC < −0.5 and p value < 0.05); a total of 1729 genes were identified, and a volcano plot (Fig 3B) was constructed based on the results of the variance analysis of this dataset. A total of 2570 DEGs in the GSE179285 dataset met the thresholds of a |logFC| > 0.5 and a p value < 0.05. When these thresholds were used, a total of 1249 genes were upregulated (logFC > 0.5 and p value < 0.05), whereas 1321 genes were downregulated (logFC < −0.5 and p value < 0.05). A volcano map was created based on the results derived from the differential expression analysis of this dataset (Fig 3C).

After all the DEGs screened in the GEO dataset (Combined Datasets) whose |logFC| was > 0.5 and p value was < 0.05 were integrated with the highly ranked genes obtained from the GSE179285 and GSE75214 datasets after differential analysis and then RRA sorting and DExMA analysis, Venn diagrams were constructed (Fig 3D), and a total of 273 highly ranked genes were obtained, as shown in S11 Table.

We subsequently intersected these DEGs with MRGs and HRGs from all UC samples. The overlapping genes were visualized in a Venn diagram (Fig 3E). A total of 60 MHRDEGs were identified, and detailed information can be found in S6 Table. Based on the results of the intersection analysis, a heatmap was generated using the R package pheatmap (Fig 3F).

### Gene ontology (GO) and pathway (KEGG) enrichment analyzes

**Fig 4:**
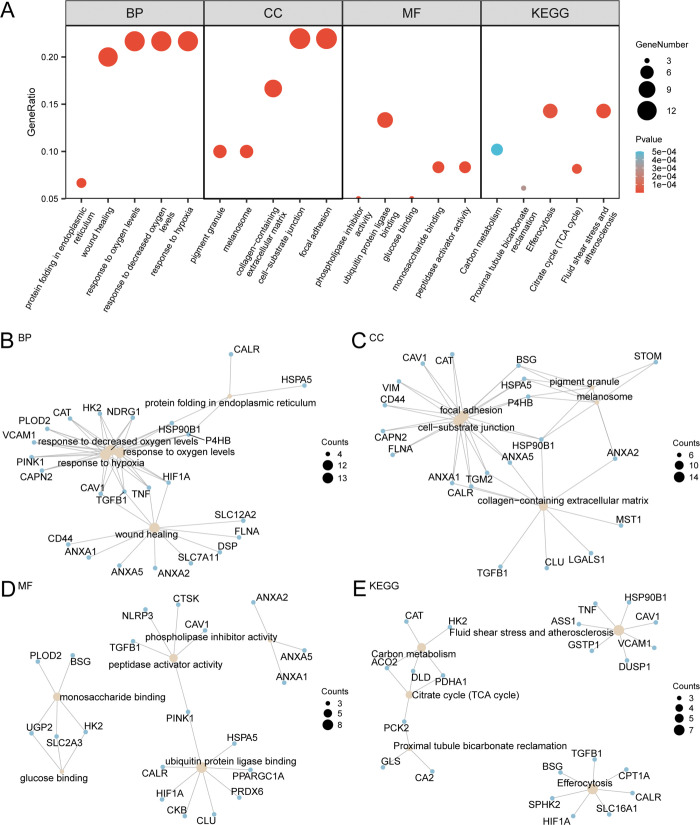
GO and KEGG enrichment analyzes. A. Bubble plot of the results of the GO and KEGG enrichment analyzes of differentially expressed genes related to mitophagy and hypoxia: BP, CC, and MF terms and KEGG pathways. GO terms and KEGG pathways are shown on the abscissa. B-E. The results of the GO and KEGG enrichment analyzes of genes related to mitophagy and hypoxia are shown in the network diagram: BP (B), CC (C), and MF (D) terms and KEGG pathways (E). The light yellow nodes represent items, the light blue nodes represent molecules, and the connecting lines indicate the relationships between items and molecules. GO, Gene Ontology; KEGG, Kyoto Encyclopedia of Genes and Genomes; BP, biological process; CC, cellular component; MF, molecular function. In the bubble plot, the size of the bubbles represents the number of genes, whereas the color of the bubbles indicates the size of the p value. The redder the color is, the smaller the p value, and the bluer the color is, the larger the p value. The screening criteria for the GO and KEGG enrichment analyzes were a p value < 0.05 and an FDR value (q value) < 0.25.

GO and KEGG pathway enrichment analyzes were performed to explore the associations among BP, CC, MF, and KEGG pathways related to the 60 DEGs linked to mitophagy and hypoxia in UC. A total of 60 genes identified as MHRDEGs were subjected to GO and KEGG enrichment analyzes, and the detailed findings are presented in S2 Table. The results revealed that the 60 MHRDEGs were predominantly enriched in the response to oxygen levels, wound healing, protein folding in the endoplasmic reticulum and other BPs; focal adhesion, cell-substrate junction, collagen-containing extracellular matrix, melanosome, pigment granule and other CCs; and peptidase activator activity, monosaccharide binding, glucose binding, ubiquitin protein ligase binding, phospholipase inhibitor activity and other MFs in the UC group. They were also enriched in fluid shear stress and atherosclerosis, the citrate cycle (TCA cycle), efferocytosis, proximal tubule bicarbonate regeneration, carbon metabolism and other biological pathways (KEGG). The findings from the GO and KEGG pathway enrichment analyzes are depicted in bubble charts (Fig 4A).

Simultaneously, network diagrams presenting the BP, CC, and MF terms and KEGG pathways were constructed based on the results of the GO and KEGG enrichment analyzes (Fig 4B–E). The network diagram shows the relationships between molecules and their annotations. The size of the nodes corresponds to the number of molecules in each entry, with larger nodes indicating a greater number of associated molecules.

### Gene set enrichment analysis (GSEA)

**Fig 5:**
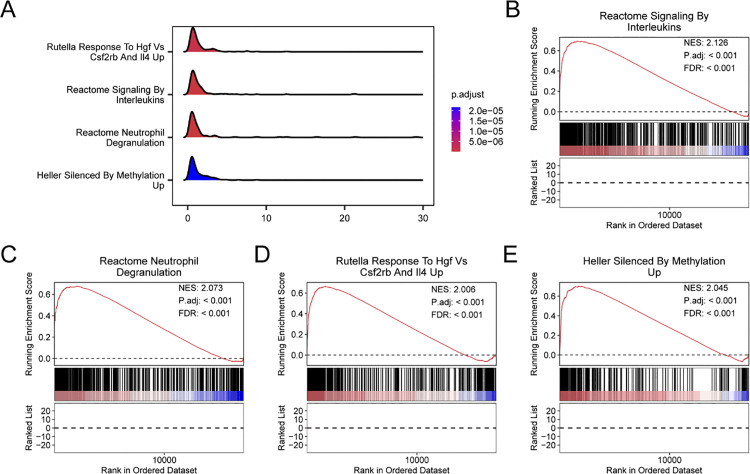
GSEA of ulcerative colitis. A. Mountain plots showing four biological functions from the GSEA of the combined GEO datasets. B–E. GSEA revealed that all genes were significantly enriched in the reactome signaling pathways involving interleukins (B), neutrophil degranulation (C), the response of the retina to Hgf vs. Csf2rb and Il4 (D), and Heller silencing by methylation (E). GSEA, gene set enrichment analysis. In GSEA, the standards for screening included an adjusted p value less than 0.05 and an FDR (q value) less than 0.25. The Benjamini–Hochberg (BH) method was used for p value correction, and the FDR was controlled at 0.25.

GSEA was performed on all genes between the UC and control groups within the combined datasets based on the logFC values to determine the impact of the expression levels of all genes in the combined datasets on UC. The results are illustrated in the mountain plot (Fig 5A). The comprehensive findings are presented in S3 Table. The analysis indicated that every gene within the combined datasets exhibited notable enrichment in the Reactome pathway associated with signaling by interleukins, as illustrated in Fig 5B. Notably, neutrophil degranulation was prominently represented (Fig 5C), the response of the Rutella to hgf vs. csf2rb and Il4 increased (Fig 5D), and it was more strongly silenced by methylation (Fig 5E); moreover, various biologically significant functions and signaling cascades were identified.

### Establishment of a diagnostic model for ulcerative colitis

**Fig 6:**
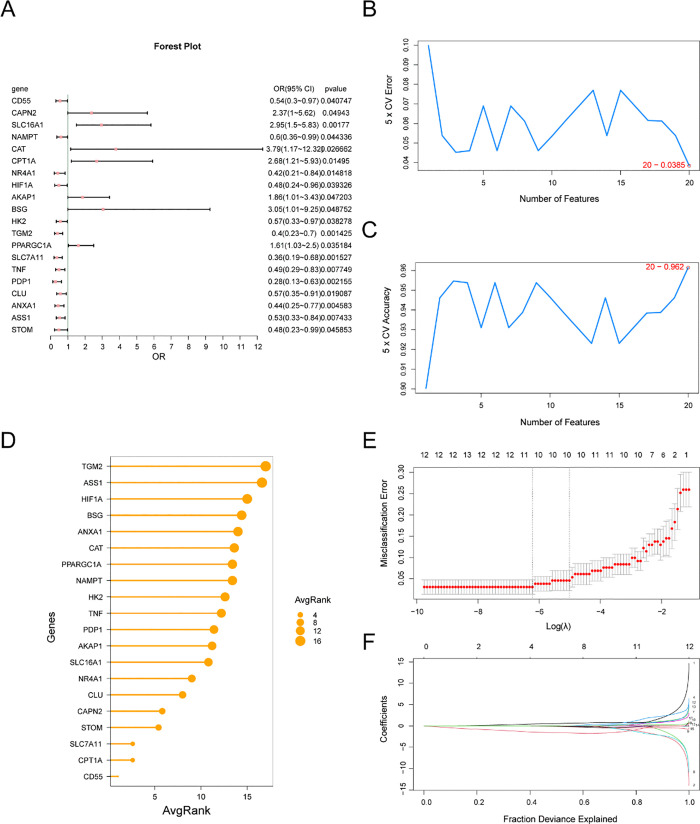
Diagnostic model of UC. A. Forest plot of 20 differentially expressed genes related to mitophagy and hypoxia included in the logistic regression model for UC diagnosis. B–C. The number of genes achieving the lowest error rate (B) and the number of genes achieving the highest accuracy (C) were visualized using the SVM-RFE algorithm. D. The average importance ranking of the top 20 genes, as determined by the SVM-RFE algorithm, was visualized in a lollipop plot. E–F. Diagnostic plot of the LASSO regression model (E): the horizontal coordinate represents the log(λ) value, and the vertical coordinate represents the binomial deviation. The red dashed line represents the optimal λ value with minimum error, and the gray line represents the standard error. LASSO; variable trajectory plot (F): Each colored curve corresponds to a key gene; the horizontal coordinate is log(λ), and the vertical coordinate is the coefficient value. The vertical dashed line indicates the selected value of λ. UC, ulcerative colitis; SVM, support vector machine; LASSO, least absolute shrinkage and selection operator.

Logistic regression analysis was first performed to evaluate the diagnostic value of the 60 MHRDEGs for UC. The findings are displayed in a forest plot (Fig 6A). The results of the logistic regression model revealed that 20 genes linked to autophagy and hypoxia were significantly differentially expressed (p value < 0.05). These genes were *CD55, CAPN2, SLC16A1, NAMPT, CAT, CPT1A, NR4A1, HIF1A, AKAP1, BSG, HK2, TGM2, PPARGC1A, SLC7A11, TNF, PDP1, CLU, ANXA1, ASS1,* and *STOM.* Second, using the 20 MRDEGs as a foundation, the SVM-RFE algorithm was employed to establish 5-fold cross-validation. The average rank of the genes was calculated, and the number of genes that achieved the lowest error rate (Fig 6B) and the highest accuracy rate (Fig 6C) in the model was determined. The results revealed that the SVM model achieved the highest accuracy when the number of genes was 20. Therefore, the top 20 genes, based on their average rank, were further studied (Fig 6D). The 20 genes were as follows: *CD55, CAPN2, SLC16A1, NAMPT, CAT, CPT1A, NR4A1, HIF1A, AKAP1, BSG, HK2, TGM2, PPARGC1A, SLC7A11, TNF, PDP1, CLU, ANXA1, ASS1,* and *STOM.* Using the 20 genes selected for the SVM model, LASSO regression was performed to construct a diagnostic model for UC. A LASSO regression model diagram (Fig 6E) and a LASSO variable trajectory diagram (Fig 6F) were generated to facilitate visualization. The results showed that the LASSO regression model included the following 10 genes related to mitophagy and hypoxia, which were identified as the key genes: *CD55, CPT1A, SLC7A11, STOM, CAPN2, NR4A1, AKAP1, PDP1, HK2,* and *ASS1.*

In addition, a detailed description of the cross-validation methodology has been added to the Materials and Methods; sensitivity, specificity, precision, and recall are in the S10 Table Supplement.

### Validation of the diagnostic model for ulcerative colitis

**Fig 7:**
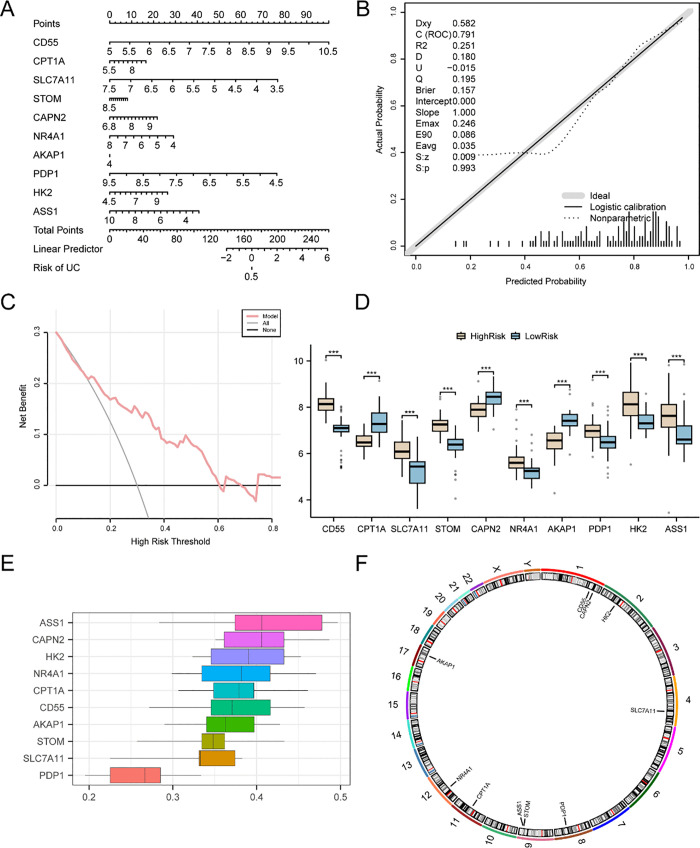
Analysis and validation of the UC diagnostic model. A. Nomograms of genes included in the UC diagnostic model based on the combined GEO datasets. B. Plot of the calibration curves for genes from the merged GEO dataset that were included in the model: the black diagonal line represents the ideal calibration, the red curve represents the actual model performance, and the blue points represent the observation probabilities. C. Decision curve analysis (DCA) plot of modeled genes from the merged GEO dataset: the vertical coordinate is the net benefit, and the horizontal coordinate is the threshold probability; the red curve represents the combined model, the gray line indicates the all-treatment scenario, and the black line indicates the no-treatment scenario; all colors and symbols are defined in the figure legend. D. Comparison of key genes between the high-risk and low-risk cohorts of patients with UC. E. Box plot of the functional similarity (Friends) analysis of key genes. The y-axis of the DCA represents the net benefit, while the x-axis represents the threshold probability. *** represents a p value < 0.001 and is highly statistically significant.

A nomogram based on key genes was created to illustrate the relationships of key genes in the combined datasets and to further validate the diagnostic model for UC (Fig 7A). The results showed that compared with the other variables, *CD55* expression had the highest utility in the diagnostic model of UC, whereas *AKAP1* expression had the lowest utility.

A calibration curve was generated through a calibration analysis to evaluate the accuracy and discrimination of the diagnostic model for UC. The predictive performance of the model was evaluated by examining the alignment between the actual and predicted probabilities under various conditions, as shown in Fig 7B. The calibration curve for the diagnostic model of UC shows that the calibration line slightly deviates from the ideal diagonal line but remains close to the fitted line. DCA was employed to assess the clinical utility of the UC diagnostic models using key genes from the combined datasets (Fig 7C). The results revealed that within a specific range, the performance of the model was consistently superior to that of both the all-positive and all-negative strategies.

The UC group was categorized into high-risk and low-risk groups based on the median risk score from the UC diagnostic model. The risk score was derived through the application of the following formula:


$$\begin{array}{c} RiskScore=CD\text{5}\text{5}\;*\;(\text{2}.\text{4618})\;+\;CPT\text{1}A\;*\;(-\text{2}.\text{7127})\;+\;SLC\text{7}A\text{1}\text{1}\;*\;(-\text{1}.\text{6616})\;+\;STOM\;\\ \;\;\;\;\;*\;(\text{2}.\text{4943})\;+\;CAPN\text{2}\;*\;(-\text{2}.\text{4889})\;+\;NR\text{4}A\text{1}\;*\;(\text{0}.\text{9492})+\;AKAP\text{1}\;*\;(-\text{0}.\text{4715})\;\\ \;\;\;\;\;+\;PDP\text{1}\;*\;(\text{1}.\text{2365})\;+\;HK\text{2}\;*\;(\text{1}.\text{1045})\;+\;ASS\text{1}\;*\;(\text{0}.\text{2151}) \end{array}$$

Comparative analysis was performed to analyze the differential expression of key genes within the UC cohort, which revealed the expression levels of ten pivotal genes between the high-risk and low-risk UC groups (Fig 7D). The comparison of the results (Fig 7E) indicated that the expression levels of the 10 key genes were highly significantly different (p value < 0.001) between the high-risk and low-risk groups.

The results of the functional similarity (Friends) analysis were used to identify genes that play significant roles in the biological processes of UC (Fig 7E). These findings indicated that *ASS1* is particularly important in UC and is the gene closest to the critical threshold (cutoff value = 0.50).

Chromosome localization analysis of ten significant genes was performed using the R package RCircos, which generated a chromosome localization map, as shown in Fig 7F. Chromosome mapping revealed that the majority of key genes, including *CD55, CAPN2, ASS1*, and *STOM*, were mapped to chromosome 1 and chromosome 9.

### Validation of the differential expression of key genes

**Fig 8:**
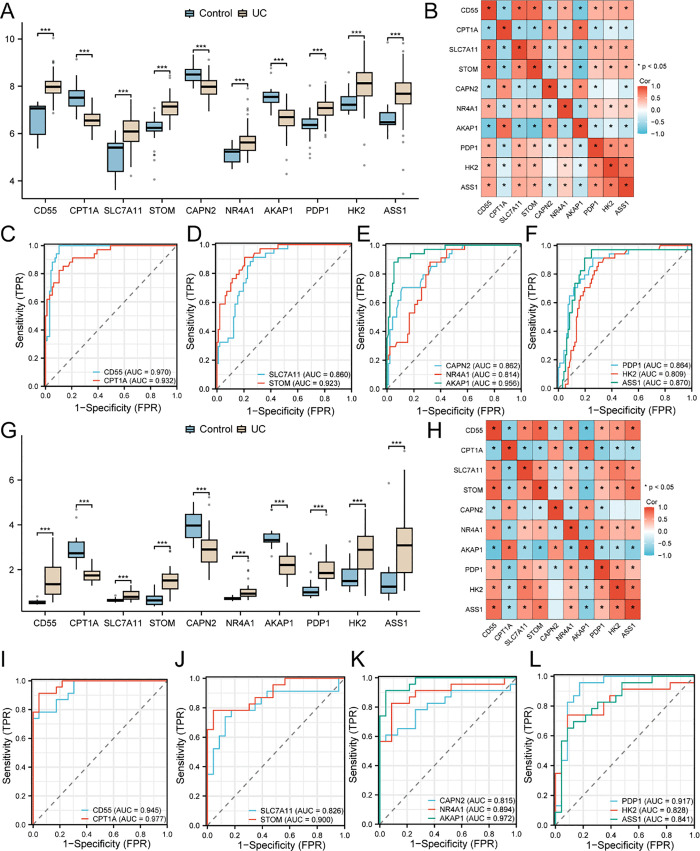
Validation of differentially expressed genes. A. Plots comparing the expression of the key genes in the UC and control groups in the combined GEO datasets. B. Heatmap of the associations between key genes in the integrated GEO datasets. C–F. ROC curves for *CD55*, *CPT1A* (C), *SLC7A11*, *STOM* (D), *CAPN2*, *NR4A1*, and *AKAP1* (E) as well as *PDP1*, *HK2*, and *ASS1* (F) in the combined GEO dataset. G. Plots comparing the expression of key genes in the UC and control groups in the GSE179285 dataset. H. Heatmap showing the correlations of key genes in the GSE179285 dataset. *CD55* and *CPT1A* (I) in key genes; *SLC7A11* and *STOM* (J); *CAPN2*, *NR4A1* and *AKAP1* (K); ROC curves for *PDP1*, *HK2*, and *ASS1* (L) in the GSE179285 dataset. *** represents a p value < 0.001 and is highly statistically significant. When the AUC is greater than 0.5, the expression of the molecule tends to promote the occurrence of the event. The closer the AUC is to 1, the better the diagnostic performance. An AUC between 0.7 and 0.9 indicated moderate diagnostic accuracy, whereas an AUC above 0.9 indicated high diagnostic accuracy. ROC, receiver operating characteristic; AUC, area under the curve; TPR, true positive rate; FPR, false positive rate; UC, ulcerative colitis. Light blue indicates the control group, while light yellow corresponds to the UC group. Red corresponds to a positive correlation, while blue corresponds to a negative correlation. The strength of the correlation is indicated by the color depth, with a moderate correlation defined as an r value between 0.5 and 0.8 and a strong correlation defined as an r value greater than 0.8.

The differential expression levels of ten pivotal genes derived from the integrated combined datasets were compared between the UC group and the control group to explore the differences in the expression of key genes (*CD55, CPT1A, SLC7A11, STOM, CAPN2, NR4A1, AKAP1, PDP1, HK2, ASS1*) in the combined datasets, as shown in Fig 8A. The results of the differential expression analysis (Fig 8A) revealed that the expression levels of the ten key genes differed significantly between the UC group and the control group in the combined GEO datasets, with a p value < 0.001.

Afterward, we performed a correlation analysis and generated a correlation heatmap for the expression of the 10 key genes in the combined datasets (Fig 8B). Among them, *CD55* and *SLC7A11* exhibited the strongest positive correlation (r = 0.80, p < 0.05), while *STOM* and *AKAP1* showed the strongest negative correlation (r = −0.78, p < 0.05).

The R package pROC was used to generate ROC curves based on the expression levels of key genes in the combined datasets. The ROC curves (Fig 8C–F) revealed that the expression levels of *SLC7A11, CAPN2, NR4A1, PDP1, HK2* and *ASS1* among the key genes accurately classified the UC group and control group (0.7 < AUC < 0.9). The expression levels of *CD55, CPT1A, STOM and AKAP1* were highly accurate (AUC > 0.9) in the classification of the UC group and control group.

A comparative analysis was performed to explore the differences in the expression of key genes (*CD55, CPT1A, SLC7A11, STOM, CAPN2, NR4A1, AKAP1, PDP1, HK2, ASS1*) in the GSE179285 dataset, and the results presented in Fig 8G illustrate the differential expression levels of ten pivotal genes between the UC group and the control group, as derived from the GSE179285 dataset. The results revealed that the expression levels of ten pivotal genes differed significantly between the UC group and the control group in the GSE179285 dataset (p value < 0.001).

Similarly, we conducted a correlation analysis and generated a correlation heatmap for the expression of the 10 key genes in the GSE179285 dataset (Fig 8H). Correlation analysis revealed that *CD55* and *STOM* were most strongly positively correlated (r = 0.83, p < 0.05), whereas AKAP1 was least strongly negatively correlated (r = −0.81, p < 0.05).

The R package pROC was applied to produce the ROC curves using the expression levels of key genes in the GSE179285 dataset. The ROC curves (Fig 8I–L) revealed that the expression levels of *SLC7A11, CAPN2, NR4A1, HK2* and *ASS1* among the key genes accurately classified the UC group and control group (0.7 < AUC < 0.9). The expression levels of *CD55, CPT1A, STOM, AKAP1* and *PDP1* were highly accurate (AUC > 0.9) for the classification of the UC group and control group.

Notably, we observed that these 10 key genes maintained high diagnostic performance in the original validation set (GSE179285), which was completely independent and did not undergo any preprocessing operations. These results suggest that the model may capture transcriptomic features with some generalization ability in ulcerative colitis patients rather than merely overfitting them to technical fluctuations specific to the training set. The good performance of this model across datasets also reduces the likelihood of overfitting to some extent, providing initial support for the biological significance of relevant markers.

### Analysis of immune cell infiltration using the ssGSEA algorithm based on logistic risk score grouping

**Fig 9:**
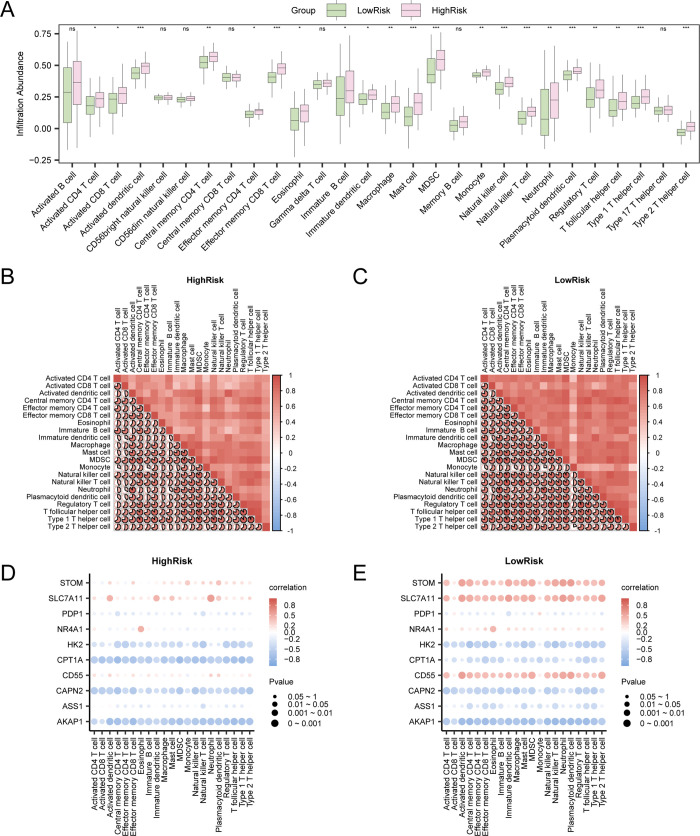
Analysis of immune cell infiltration using the ssGSEA algorithm. A. Comparison of immune cells in the low-risk and high-risk groups within the combined GEO datasets. B–C. The results of the correlation analysis for immune cell infiltration levels in the high-risk (B) and low-risk (C) groups of UC patients are presented. D–E. The correlations between immune cell infiltration levels and the expression of key genes in the high-risk (D) and low-risk (E) groups of UC patients are shown in a bubble chart. ssGSEA, single-sample gene-set enrichment analysis; UC, ulcerative colitis. ns represents a p value ≥ 0.05 that is not statistically significant; * represents a p value < 0.05 that is statistically significant; ** represents a p value < 0.01 that is highly statistically significant; and *** represents a p value < 0.001 that is highly statistically significant. An absolute correlation coefficient (r value) less than 0.3 indicated a weak or no correlation, a value between 0.3 and 0.5 indicated a weak correlation, a value between 0.5 and 0.8 indicated a moderate correlation, and a value greater than 0.8 indicated a strong correlation. Light green indicates the low-risk group, while light pink indicates the high-risk group. Red corresponds to a positive correlation, blue corresponds to a negative correlation, and the intensity of the color reflects the magnitude of the correlation.

The ssGSEA algorithm was employed to assess the levels of infiltration of 28 distinct immune cell types in UC samples utilizing the expression matrices derived from the combined GEO datasets of UC samples. A comparative plot (Fig 9A) revealed that all 21 immune cell types were significantly different (p < 0.05) and included activated CD4^+^ T cells, activated CD8^+^ T cells, activated dendritic cells, central memory CD4^+^ T cells, effector memory CD8^+^ T cells, eosinophils, immature B cells, immature dendritic cells, macrophages, mast cells, myeloid-derived suppressor cells (MDSCs), monocytes, natural killer cells, natural killer T cells, neutrophils, plasmacytoid dendritic cells, regulatory T cells, T follicular helper cells, and type 1 T helper cells. The results of the correlation analysis showing the abundance of 21 types of infiltrating immune cells in UC samples are illustrated in a correlation heatmap (Fig 9B–C). The results indicated that most immune cells in the high-risk group were strongly correlated, with T follicular helper cells and type 1 T helper cells showing the strongest significant positive correlation (r = 0.932, p < 0.05) (Fig 9B). The results revealed that most immune cells in the low-risk group of UC samples were strongly correlated. Specifically, the strongest positive correlation was observed between regulatory T cells and natural killer cells (r = 0.949, p < 0.05) (Fig 9C). The correlations between key genes and immune cell infiltration levels were visualized in a correlation bubble chart (Fig 9D–E). Most immune cells in the high-risk group were strongly correlated, with the *CPT1A* gene and MDSCs showing the strongest significant negative correlation (r = −0.726, p < 0.05) (Fig 9D). The correlation bubble plot results demonstrated that most immune cells in the low-risk group of UC samples were strongly correlated, with the *AKAP1* gene and natural killer T cells showing the strongest significant negative correlation (r = −0.765, p < 0.05) (Fig 9E).

### Analysis of mRNA–TF and mRNA–miRNA regulatory networks of key genes

**Fig 10:**
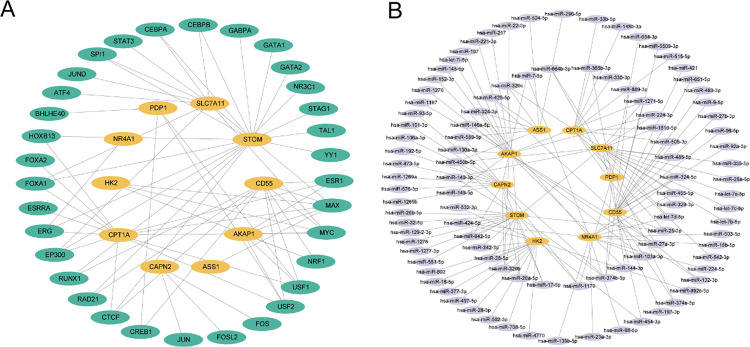
Regulatory network of key genes. A. Regulatory network of mRNAs and TFs for key genes. B. Regulatory network involving mRNAs and miRNAs for key genes. TF, transcription factor; RBP, RNA-binding protein. Orange represents mRNAs, green represents TFs, and purple represents miRNAs.

TFs that interact with key genes were retrieved from the ChIPBase database. The mRNA–TF regulatory network was subsequently constructed and visualized using Cytoscape software, as shown in Fig. 10A. The network is composed of 10 key genes and 68 TFs, with additional details presented in S7 Table.

The miRNAs linked to the key genes were retrieved from the StarBase database. The mRNA–miRNA regulatory network was subsequently constructed and visualized using Cytoscape software, as shown in Fig. 10B. The analysis included a total of 10 key genes and 161 miRNAs, and additional details are provided in S8 Table.

### Establishment and characterization of a TNBS-induced colitis model in rats

**Fig 11:**
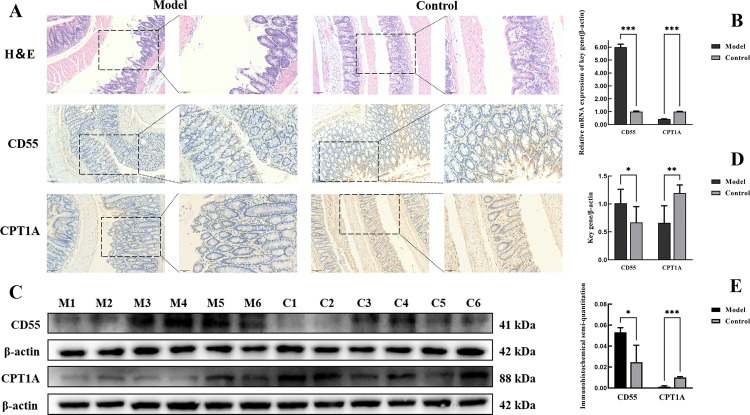
Pathological changes and expression of key genes in rats with TNBS-induced colitis. A. Representative images of H&E staining and immunohistochemical staining at the level of *CD55* and *CPT1A* in normal and UC groups of rats; scale bars, 100 μm (left panel) and 50 μm (right panel). B. Histogram showing the comparison of the results of RT–qPCR assays of the expression of key genes in the intestines of normal rats and UC rats. C. Western blot analysis of the expression levels of *CD55* and *CPT1A* in the intestines of normal and UC rats. D. Histogram showing the protein expression levels of *CD55* and *CPT1A* in normal and UC rats. E. Histogram showing the immunohistochemical AOD values of *CD55* and CPT1A in normal rats and UC rats. *** p < 0.001; ** p < 0.01; and * p < 0.05.

We induced UC in rats via a TNBS enema for 7 days, while control mice were administered distilled water to investigate the roles of selected key genes in mitochondrial autophagy and hypoxia and their association with UC. Rats treated with TNBS showed typical UC symptoms, including behavioral changes, blood in the stool, and wasting. Compared with the model group, the blank group showed normal performance. Afterward, we observed the colon samples of the two groups using H&E staining, which revealed significant pathological differences between the two groups (Fig 11A). UC rats exhibit obvious inflammatory cell infiltration and destruction of the intestinal mucosal epithelium, which are key pathological changes that occur during the development of UC. These results confirmed the successful establishment of the UC model and provided a basis for further studies.

### Validation of the expression of key genes in colon samples from UC rats

RT–qPCR was performed to verify the differences in the expression of key genes in the colon tissues of UC rats and healthy rats, and significant differences in the expression of *CD55* and *CPT1A* were detected between UC rats and healthy rats (Fig 11B). Subsequent Western blot and immunohistochemical staining again verified that, compared with that in normal tissues, the expression of *CD55* was increased in the colons of UC rats. In contrast, *CPT1A* expression decreased (p < 0.05) (Fig 11A, C, D). These findings further suggest that *CD55* and *CPT1A* may play crucial roles in the disease process and pathogenesis of UC. The potential of these genes as biomarkers deserves further exploration in future studies.

## Discussion

Due to its increasing incidence, UC has become one of the most common inflammatory bowel diseases worldwide [2]. The symptoms of UC are distressing and are accompanied by an increased risk of colorectal cancer. Additionally, its complex pathogenesis makes treatment challenging, placing a significant economic burden on society [3]. Currently, no gold standard exists for UC diagnosis. Owing to the nonspecific nature of its symptoms, clinicians struggle to distinguish functional bowel disorders (e.g., functional dyspepsia and irritable bowel syndrome) from potential UC cases. More than 50% of patients with functional diagnoses are subjected to unnecessary colonoscopies [40,41]. Furthermore, the differential diagnosis between UC and Crohn’s disease (CD) remains uncertain [41,42]. While an endoscopic examination is generally considered intuitive and definitive for a UC diagnosis, it is not only expensive and uncomfortable but also has a risk of perforation. Notably, patients with early-stage UC often exhibit colonic inflammation without the presence of ulcers or erosions [43,44]. Therefore, exploring the mechanisms underlying UC and identifying new, specific diagnostic strategies remain critical and complex areas of UC research.

Hypoxia and mitophagy are crucial for UC pathogenesis and the regulation of the immune response [10–12,45] and these processes have attracted significant research interest. However, the underlying mechanisms of mitophagy and hypoxia in UC pathogenesis, as well as their potential as therapeutic targets, remain largely unexplored. Our study explored the diagnostic and prognostic significance of HRGs and MRGs in UC pathogenesis, identified potential hub genes, and investigated possible regulatory targets.

An analysis of GEO datasets comparing UC patients with healthy controls revealed 2,276 DEGs, including 1,196 upregulated genes and 1,080 downregulated genes. Afterward, RRA and DEXMA were used for complementary analysis. Notably, 273 genes were consistently identified as differentially expressed genes in all three independent methods. These genes may be highly robust and reproducible in UC and are largely independent of specific data analysis methods or batch effects. Although these 273 genes with high methodological robustness have limited overlap with the 60 genes screened based on specific biological assumptions (mitochondrial autophagy vs. hypoxia), this result has important independent value. These results may shed further light on the different levels of biological mechanisms involved in the pathological process of UC. These 273 genes may contain effectors that drive the most basic phenotypes of the UC inflammatory response, mucosal destruction and repair. The mitochondrial autophagy- and hypoxia-related genes that are the focus of this study may play regulatory or supportive roles in the development of UC; their importance lies in their overall role as functional modules in response to the intestinal inflammatory environment and in the regulation of cellular energy metabolism. Thus, the discovery of these 273 genes provides a high-confidence reference list of core genes for UC studies, which can be deeply mined and experimentally validated in future studies. Our in-depth exploration of 60 mitochondrial autophagy and hypoxia-related genes builds on this reliability to provide insight into a specific biological mechanism.

After confirming the robustness of our overall analytical framework, we further investigated the role of the specific mechanism of mitochondrial autophagy and hypoxia in UC. LASSO logistic regression (to minimize classification errors) and support vector machine-recursive feature elimination (SVM-RFE, for ranking and selecting key features) were used in this study to identify diagnostic biomarkers for UC. Ultimately, *CD55* and *CPT1A* had the most significant AUC values (Fig 8C, I) and were identified as core diagnostic biomarkers for UC.

In support of the experimental validation data, compared with the normal control group, the present study revealed increased expression of *CD55* in the ulcerative colitis group and decreased expression of *CPT1A* in the UC group, emphasizing the significant association between these two key genes and UC pathology. These findings open new avenues for clarifying the pathological mechanisms of UC and provide potential biomarkers. *CD55*, a glycosylphosphatidylinositol-anchored protein, inhibits complement pathway activation by accelerating the decay of C3/C5 convertases through binding to C3b/C4b [46]. Our findings corroborate previous reports of elevated fecal *CD55* levels in UC patients, particularly those with active disease [47,48]. The hypoxic intestinal microenvironment is maintained by HIF-1α to ensure barrier integrity and metabolic function, and HIF-1α further accumulates during inflammation [49]. HIF-1α upregulates *CD55* expression under hypoxic conditions [50] while simultaneously activating BNIP3/NIX-mediated mitophagy [51]. Although elevated HIF-1α levels in UC enterocytes correlate with disease activity, this compensatory mechanism appears insufficient to reverse disease progression [11], highlighting the potential dual role of *CD55* in the hypoxia response and mitophagy regulation and its promise as a biomarker in UC biopsy specimens. The expression of *CPT1A*, which is a rate-limiting mitochondrial enzyme for fatty acid β-oxidation, is reduced in individuals with UC. Dysregulated fatty acid metabolism disrupts the balance of inflammatory mediators [52,53], influencing the severity of intestinal inflammation [54,55]. *CPT1A* downregulation exerts protective effects on DSS-induced colitis by suppressing PPARα signaling [56] while also modulating PINK1-mediated mitophagy and reducing cellular ROS generation, thereby alleviating tissue damage induced by oxidative stress [57,58]. Notably, HIF-mediated *CPT1A* inhibition contributes to fatty acid metabolic dysregulation under hypoxic conditions [59].

Enrichment analyzes revealed that DEGs related to mitophagy and hypoxia, such as *CD55* and *CPT1A*, are primarily linked to biological processes such as the hypoxic response, wound healing, and ER protein folding, as well as molecular functions such as peptidase activator activity, monosaccharide and glucose binding, ubiquitin protein ligase binding, and phospholipase inhibitor activity (Fig 4A). The regulatory pathways involved include primarily efferocytosis, the citrate cycle (TCA cycle), and carbon metabolism (Fig 4A). The GSEA results revealed that the DEGs were enriched mainly in pathways such as signaling by interleukins, neutrophil degranulation, the response of Rutella to HGF vs. CSF2RB and IL4, and Heller silencing by methylation, all of which are related to the inflammatory response in UC. Consistent with the findings of previous studies, signaling by interleukins plays a key role in UC treatment [44,53]. Increased mucosal and serum hepatocyte growth factor (HGF) levels in patients with active UC and in DSS-induced murine models correlate with the expression of inflammatory markers [60]. Similarly, GM-CSF levels are correlated with UC disease activity and may serve as biomarkers for diagnosis and mucosal healing monitoring, while interleukin-4 (IL-4) is significantly upregulated in UC patients [61]. Moreover, Taman [62] revealed that in patients with severe UC, DNA hypomethylation acts as a key epigenetic driver, with hypomethylated genes enriched in pathways essential for neutrophil degranulation and immune regulation within the lymphatic system. Collectively, the results underscore the functional significance of these genes in UC pathogenesis, indicating that they are involved in dysregulated immune activation, epigenetic modulation, and inflammatory tissue remodeling.

Immune dysfunction and abnormal immune cell activation are central to UC pathogenesis [45]. Compared with that in the low-risk group of patients with UC, the infiltration of various immune cell subsets was significantly greater in high-risk patients. These subsets included neutrophils, activated dendritic cells, effector memory CD8^+^ T cells, mast cells, MDSCs, and type 1 and type 2 helper T cells (Fig 9A). In the pathological conditions of UC, the activation of immune cells, particularly neutrophils, CD4^+^ T-cell subsets, dendritic cells and macrophages, can promote disease development. This effect is achieved through the excessive release of proinflammatory cytokines, which in turn sustain chronic inflammation [63–65]. This sustained inflammatory response disrupts intestinal immune homeostasis, damages the mucosal barrier, increases epithelial permeability, and perpetuates chronic inflammation [63,64]. Importantly, dysregulated immune responses have been directly linked to mucosal injury and disease exacerbation [66], suggesting that targeted modulation of these immune cells could mitigate UC progression. Our study further confirmed the significant correlation between the expression of *CD55* and *CPT1A* and the levels of multiple types of immune cells (Fig 9D, E). Given the role of *CD55* in regulating complement activation and *CPT1A* in modulating mitochondrial fatty acid oxidation (FAO) to maintain mitochondrial function and antioxidant status, as well as its involvement in immune cell metabolism, both *CD55* and *CPT1A* may be involved in the activation and functional regulation of these immune cells. These findings underscore their potential as therapeutic targets. Our study revealed that the strongest negative correlation occurred between *CPT1A* expression and myeloid-derived suppressor cells (MDSCs) (Fig 9E). FAO—a key metabolic pathway for MDSC function—relies on *CPT1A* as its rate-limiting enzyme [67]. The observed *CPT1A* suppression in UC likely triggers metabolic reprogramming of MDSCs toward glycolytic pathways, an adaptive strategy to sustain energy production under chronic hypoxic and inflammatory conditions [68].

The mRNA–miRNA and mRNA–TF regulatory networks constructed in this study revealed complex interactions, including 161 miRNAs and 68 transcription factors associated with key genes. These miRNAs and transcription factors are closely related to the hypoxia response, oxidative stress, inflammatory mediator generation, mitochondrial autophagy and cell death. Together, these multilevel regulatory mechanisms contribute to the pathological progression of ulcerative colitis. In this study, *CD55* and *CPT1A* were coregulated by transcription factors such as ESR and MYC. Estrogen receptors (ESRs) play a role in the immune response in localized tissues, and estrogen receptors are dysregulated in the intestinal mucosa of IBD patients [69]. Second, the myelocytomatosis oncogene (MYC) plays an important role in the repair of the colonic mucosa during the recovery phase in UC mice [70]. The ESR can regulate the expression of *CD55* and thus affect the colonization of *E. coli*, and when activated, it can protect against the inflammation of bladder epithelial cells caused by *E. coli* infection by downregulating *CD55* expression [71]. In addition, the expression of *CPT1A* is increased in ESR-positive breast cancer cell lines [72], suggesting that ESR likely has a regulatory effect on *CPT1A*. Second, MYC can bind canonical and noncanonical e-boxes in the *CD55* promoter to regulate its transcription [73]; at the same time, an interaction has been observed between *CPT1A* and MYC, in which *CPT1A* inhibits the ubiquitination and degradation of Myc, while the transcription of Myc induces the expression of *CPT1A* [74].

The mRNA–miRNA network revealed that *CD55* and *CPT1A* were coregulated by miR-505-3p, which is a component of miR-505 located on the X chromosome. Previous studies have shown that miR-505-3p plays a key role in the regulation of cell metabolism, growth, proliferation and survival and is involved in the development of a variety of diseases [75–77]. The expression of miR-505-3p is increased in the colon tissue of a UC mouse model and is involved in the differentiation of dendritic cells toward an inflammatory phenotype [78]. In summary, this study revealed the potential roles of ESR, MYC and miR-505-3p as key upstream regulators of *CD55* and *CPT1A* in UC through the construction of mRNA–miRNA and mRNA–TF regulatory networks. Although ESR and MYC are involved in the development of UC and can regulate the expression of *CD55* and *CPT1A*, respectively, and because miR-505-3p is abnormally expressed during UC inflammation and is involved in immune cell differentiation, no systematic study of the mechanism of action of ESR, MYC, and miR-505-3p as regulators of *CD55* and *CPT1A* on the epithelial cells of UC or the colon has been performed. Therefore, the results of the present study provide possible new perspectives for an in-depth understanding of the pathogenesis of UC and provide insights for subsequent studies.

While our study provides valuable insights, its limitations must be recognized. The differences observed in the control samples of healthy individuals and those with varying risk levels of UC suggest the involvement of complex mechanisms of hypoxia and mitochondrial autophagy in UC.

Validation through animal experiments alone cannot fully elucidate the roles of these key genes in the development of UC. In the future, we plan to further confirm these findings through cellular experiments and an assessment of clinical samples to enhance the scientific rigor and application value of our findings and deepen our understanding of this complex disease.

Second, this study ensured the robustness of the results by merging the existing GEO datasets and performing an initial external validation of the model using the independent dataset GSE179285 after rigorously removing the batch effect. However, further validation based on third-party external independent datasets has not yet been performed because of the limited number of publicly available high-throughput UC datasets and platform compatibility at this stage. We will continue to focus on the updates of data resources in the future and plan to introduce new independent datasets to further validate and improve the applicability and promotion prospects of the diagnostic model when conditions allow. Validation in larger and more diverse cohorts would help to confirm the broad applicability of biomarkers. We selected the microarray dataset for analysis due to the limited availability of publicly accessible RNA sequencing data in ulcerative colitis patients. The current sample size is insufficient for robust machine learning and batch calibration analysis. We plan to use high-throughput sequencing data for further analysis once conditions permit.

## Conclusions

In summary, in this study, we combined machine learning with bioinformatics to identify *CD55* and *CPT1A* as potential diagnostic biomarkers for UC. In addition, these genes are expected to be research targets for subsequent treatment. Analysis of immune cell infiltration revealed significant associations between *CD55* and *CPT1A* expression and the infiltration of various immune cells. Furthermore, we analyzed the mRNA–TF and mRNA–miRNA regulatory networks of the key genes, further elucidating their roles in the hypoxia response, oxidative stress, inflammatory mediator production, mitophagy, and cell death. Overall, these findings underscore the unique role of our research and highlight the importance of *CD55* and *CPT1A* in UC pathogenesis and as potential biomarkers.

## Supporting information

S1 Table GEO Microarray Chip Information. (DOCX)

S2 Table The results of the GO and KEGG enrichment analyzes. (DOCX)

S3 Table The results of GSEA for the combined datasets. (DOCX)

S4 Table MRGs. (XLSX)

S5 Table HRGs. (XLSX)

S6 Table MHRDEGs. (XLSX)

S7 Table mRNA-TF. (CSV)

S8 Table mRNA‒miRNA. (CSV)

S9 Table qRT‒PCR and WB data. (XLSX)

S10 Table Model Performance Metrics. (XLSX)

S11 Table Limma and RRA and DExMA. (XLSX)

S12 Table IHC Raw Date. (XLSX)

S1 Fig HE raw_image. (ZIP)

S2 Fig CD55 IHC_raw_image. (ZIP)

S3 Fig CPT1A IHC_raw_image. (ZIP)

S4 Fig Western blot_raw_image. (PDF)

S5 Fig qRT-PCR_raw_image. (ZIP)
