# Supplementary material for: Identification of hypoxia- and mitophagy-related diagnostic biomarkers for ulcerative colitis based on bioinformatic analysis and machine learning
Source: PLoS One. 2026 Jan 21;21(1):e0339296. doi: 10.1371/journal.pone.0339296 (PMC12822963; doi:10.1371/journal.pone.0339296)
Supplement: S1 Table — (DOCX) [file pone.0339296.s001.docx]

### Table 1 GEO Microarray Chip Information

|  | GSE75214 | GSE179285 |
| --- | --- | --- |
| Platform | GPL6244 | GPL6480 |
| Species | Homo sapiens | Homo sapiens |
| Tissue | Colon | Colon |
| Samples in UC group | 74 | 23 |
| Samples in Control group | 11 | 23 |
| Reference | PMID：28885228 | PMID：34561227 |

GEO，Gene Expression Omnibus；UC，Ulcerative Colitis。
