# Supplementary material for: Identification of hypoxia- and mitophagy-related diagnostic biomarkers for ulcerative colitis based on bioinformatic analysis and machine learning
Source: PLoS One. 2026 Jan 21;21(1):e0339296. doi: 10.1371/journal.pone.0339296 (PMC12822963; doi:10.1371/journal.pone.0339296)
Supplement: S2 Table — (DOCX) [file pone.0339296.s002.docx]

### Table 2 Results of GO and KEGG Enrichment Analysis

| ONTOLOGY | ID | Description | GeneRatio | BgRatio | pvalue | qvalue |
| --- | --- | --- | --- | --- | --- | --- |
| BP | GO:0001666 | response to hypoxia | 13/60 | 298/18870 | 7.78359E-12 | 1.15607E-08 |
| BP | GO:0036293 | response to decreased oxygen levels | 13/60 | 315/18870 | 1.56105E-11 | 1.15929E-08 |
| BP | GO:0070482 | response to oxygen levels | 13/60 | 343/18870 | 4.51548E-11 | 2.23556E-08 |
| BP | GO:0042060 | wound healing | 12/60 | 423/18870 | 7.29484E-09 | 2.70869E-06 |
| BP | GO:0034975 | protein folding in endoplasmic reticulum | 4/60 | 11/18870 | 2.99671E-08 | 7.663E-06 |
| CC | GO:0005925 | focal adhesion | 14/60 | 421/19886 | 2.0959E-11 | 1.52181E-09 |
| CC | GO:0030055 | cell-substrate junction | 14/60 | 431/19886 | 2.8628E-11 | 1.52181E-09 |
| CC | GO:0062023 | collagen-containing extracellular matrix | 10/60 | 429/19886 | 5.65762E-07 | 2.00498E-05 |
| CC | GO:0042470 | melanosome | 6/60 | 112/19886 | 1.09008E-06 | 2.31786E-05 |
| CC | GO:0048770 | pigment granule | 6/60 | 112/19886 | 1.09008E-06 | 2.31786E-05 |
| MF | GO:0016504 | peptidase activator activity | 5/60 | 52/18496 | 7.00667E-07 | 0.000151197 |
| MF | GO:0048029 | monosaccharide binding | 5/60 | 75/18496 | 4.39527E-06 | 0.00035132 |
| MF | GO:0005536 | glucose binding | 3/60 | 11/18496 | 5.25671E-06 | 0.00035132 |
| MF | GO:0031625 | ubiquitin protein ligase binding | 8/60 | 308/18496 | 6.51228E-06 | 0.00035132 |
| MF | GO:0004859 | phospholipase inhibitor activity | 3/60 | 13/18496 | 9.0696E-06 | 0.000362208 |
| KEGG | hsa05418 | Fluid shear stress and atherosclerosis | 7/49 | 141/8538 | 1.38876E-05 | 0.00130335 |
| KEGG | hsa00020 | Citrate cycle (TCA cycle) | 4/49 | 30/8538 | 2.35135E-05 | 0.00130335 |
| KEGG | hsa04148 | Efferocytosis | 7/49 | 157/8538 | 2.79289E-05 | 0.00130335 |
| KEGG | hsa04964 | Proximal tubule bicarbonate reclamation | 3/49 | 23/8538 | 0.000290213 | 0.010157453 |
| KEGG | hsa01200 | Carbon metabolism | 5/49 | 116/8538 | 0.00050251 | 0.011094328 |

### GO，Gene Ontology；BP，Biological Process；CC，Cellular Component；MF，Molecular Function；KEGG，Kyoto Encyclopedia of Genes and Genomes。
