# Supplementary material for: Identification of hypoxia- and mitophagy-related diagnostic biomarkers for ulcerative colitis based on bioinformatic analysis and machine learning
Source: PLoS One. 2026 Jan 21;21(1):e0339296. doi: 10.1371/journal.pone.0339296 (PMC12822963; doi:10.1371/journal.pone.0339296)
Supplement: S3 Table — (DOCX) [file pone.0339296.s003.docx]

### Table 3 Results of GSEA for Combined Datasets

| ID | enrichmentScore | NES | pvalue | p.adjust | qvalue |
| --- | --- | --- | --- | --- | --- |
| REACTOME_SIGNALING_BY_INTERLEUKINS | 445 | 445 | 0.693959835 | 2.125881643 | 1.00E-10 |
| REACTOME_NEUTROPHIL_DEGRANULATION | 449 | 449 | 0.675528929 | 2.072674121 | 1.11E-10 |
| RUTELLA_RESPONSE_TO_HGF_VS_CSF2RB_AND_IL4_UP | 379 | 379 | 0.665486373 | 2.006046518 | 5.18E-09 |
| HELLER_SILENCED_BY_METHYLATION_UP | 262 | 262 | 0.700546301 | 2.044734427 | 1.71E-07 |

GSEA，Gene Set Enrichment Analysis
