# Supplementary figures and images for: Identification of hypoxia- and mitophagy-related diagnostic biomarkers for ulcerative colitis based on bioinformatic analysis and machine learning
Source: PLoS One. 2026 Jan 21;21(1):e0339296. doi: 10.1371/journal.pone.0339296 (PMC12822963; doi:10.1371/journal.pone.0339296)

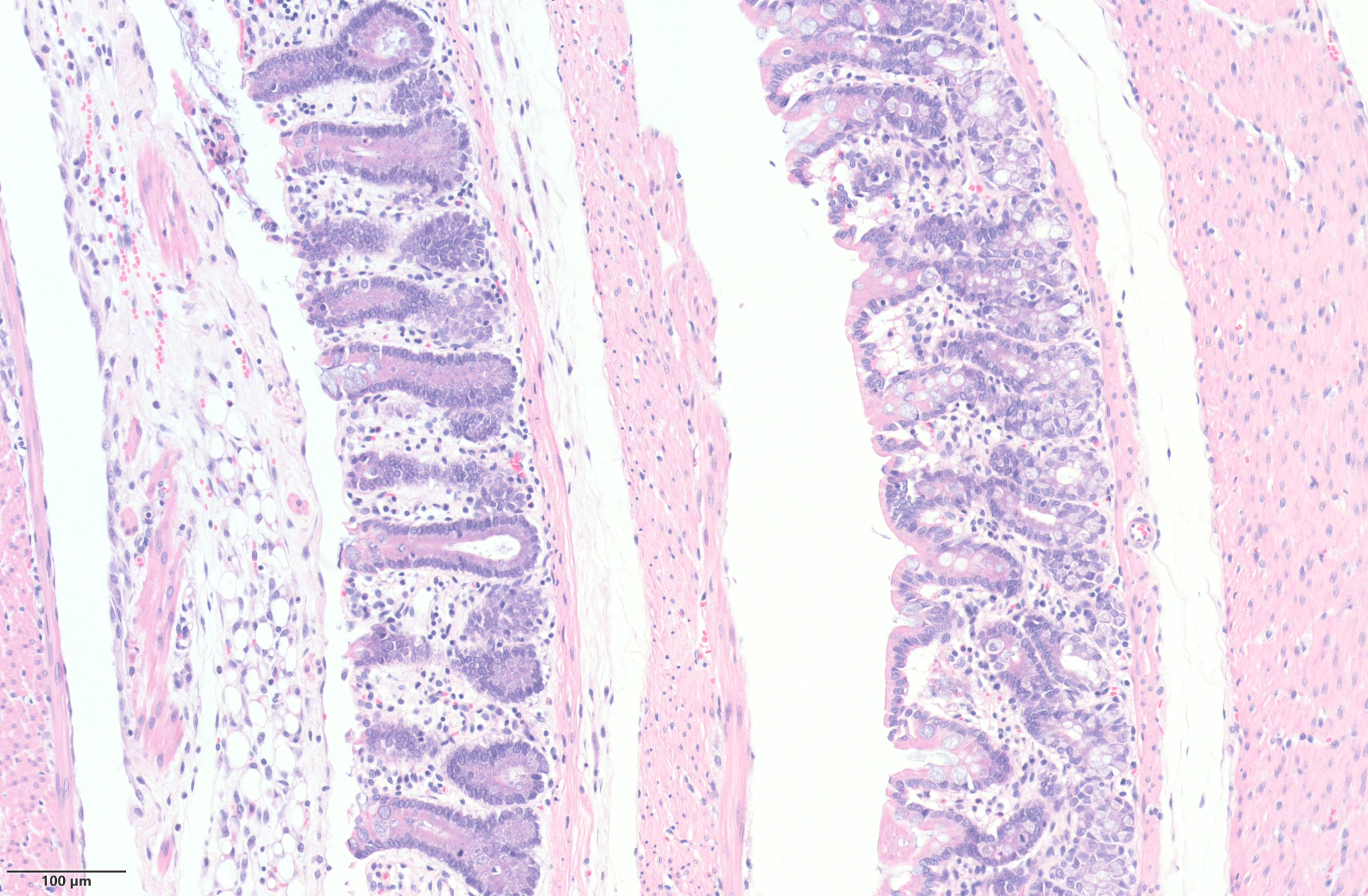

Supplement: S1 Fig — (ZIP) [file pone.0339296.s013.zip › HE/HE/Control-1-20x.tif]

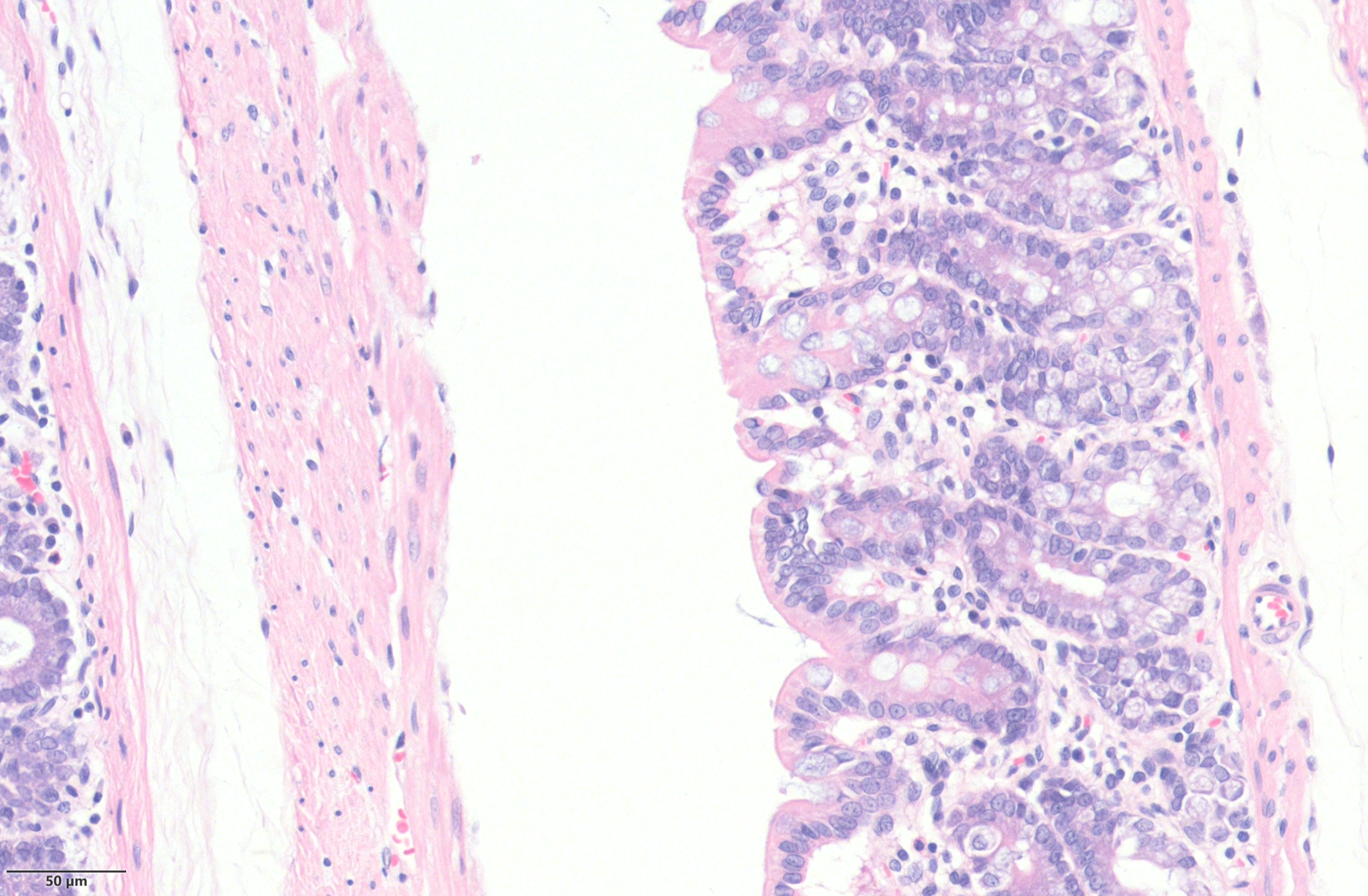

Supplement: S1 Fig — (ZIP) [file pone.0339296.s013.zip › HE/HE/Control-1-40x.tif]

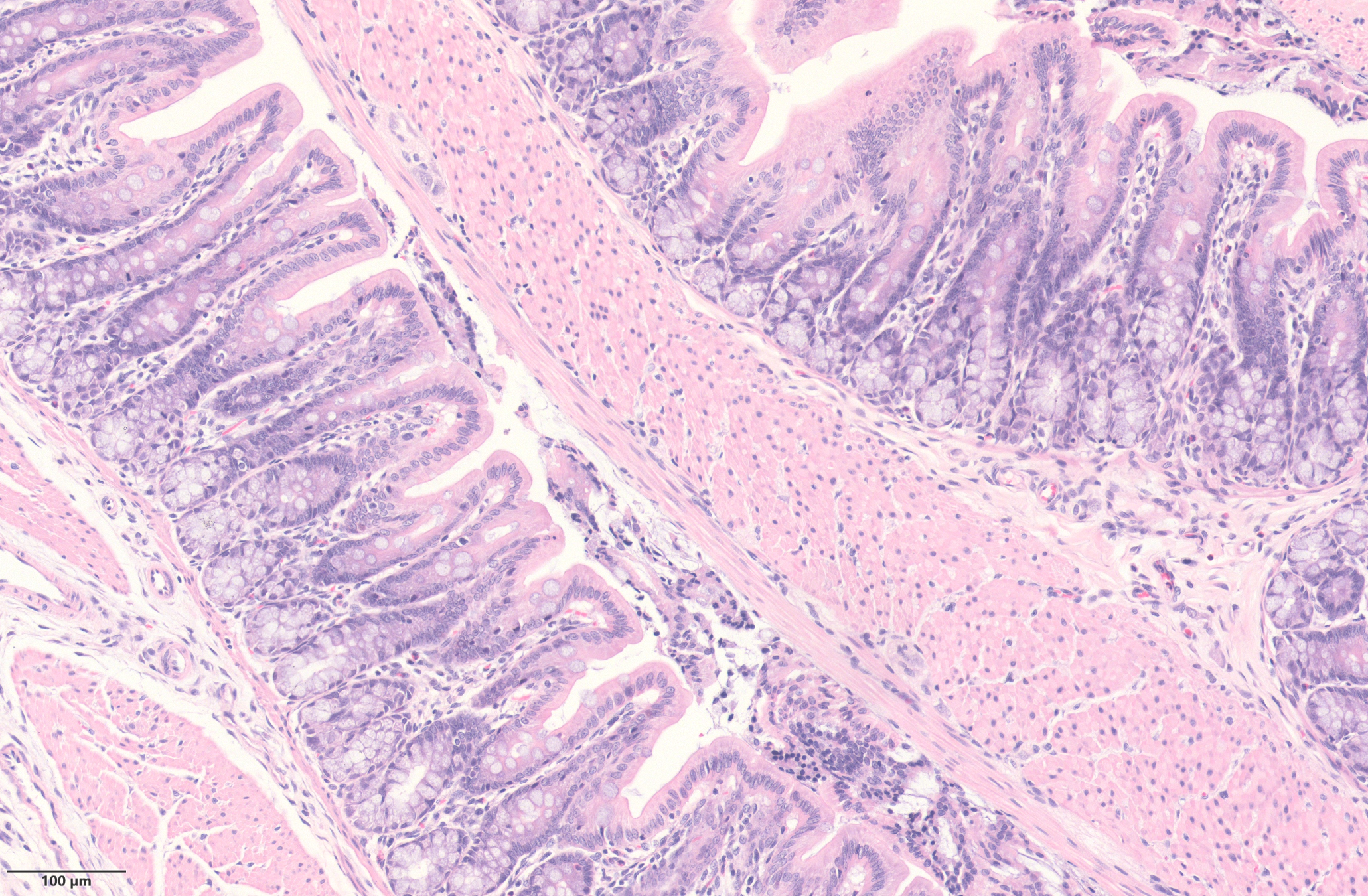

Supplement: S1 Fig — (ZIP) [file pone.0339296.s013.zip › HE/HE/Control-2-20X.tif]

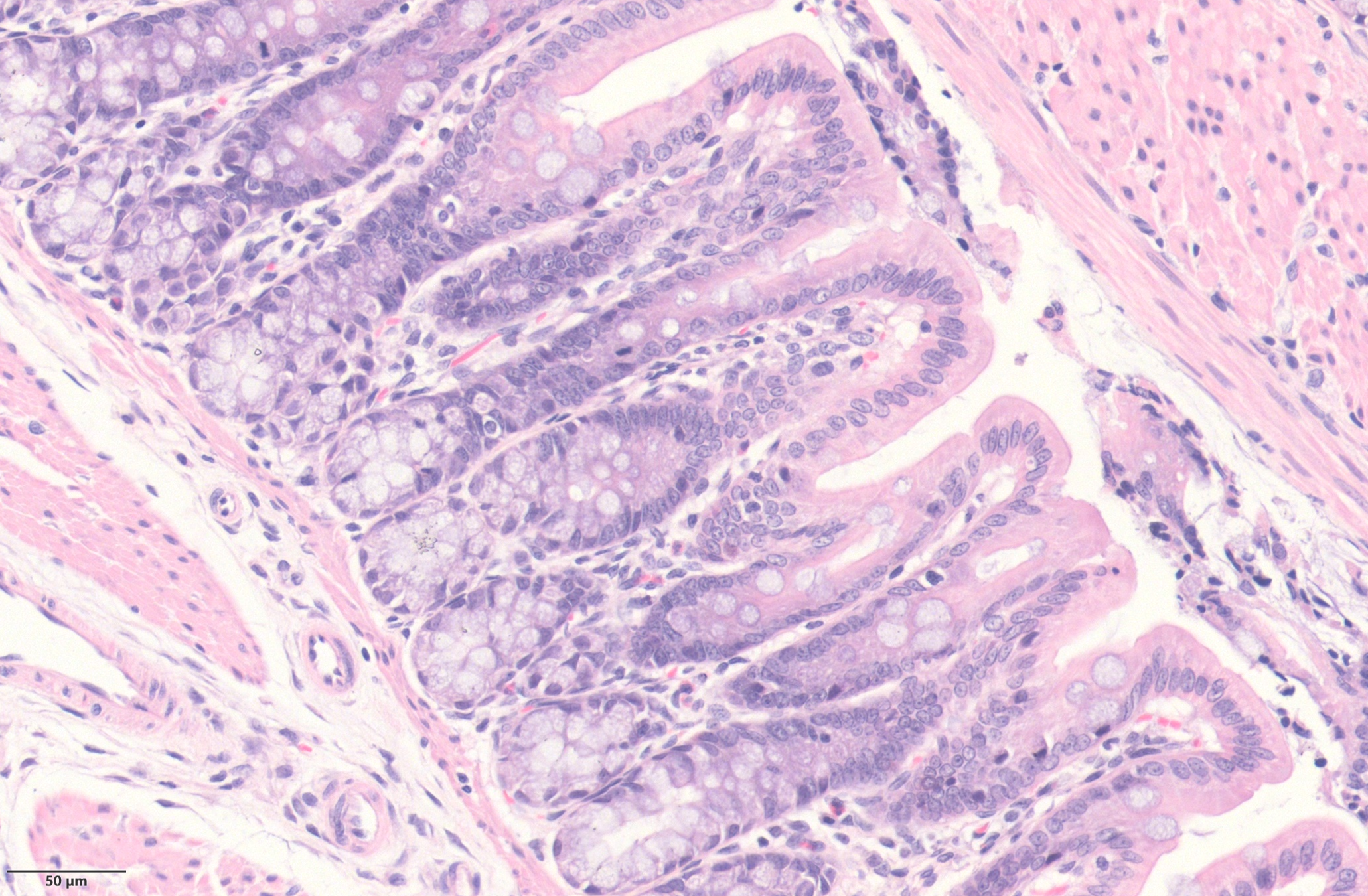

Supplement: S1 Fig — (ZIP) [file pone.0339296.s013.zip › HE/HE/Control-2-40X.tif]

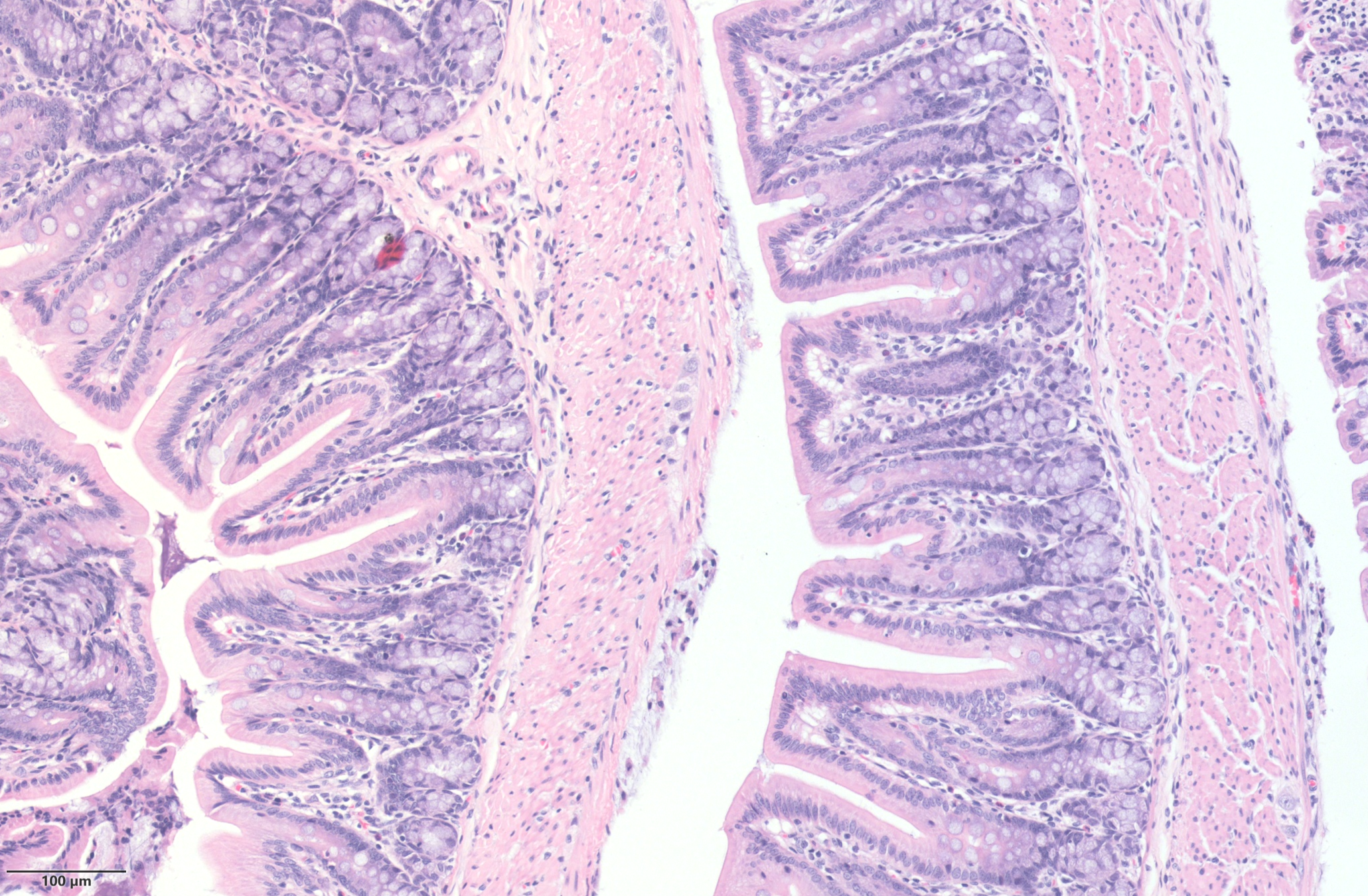

Supplement: S1 Fig — (ZIP) [file pone.0339296.s013.zip › HE/HE/Control-3-20X.tif]

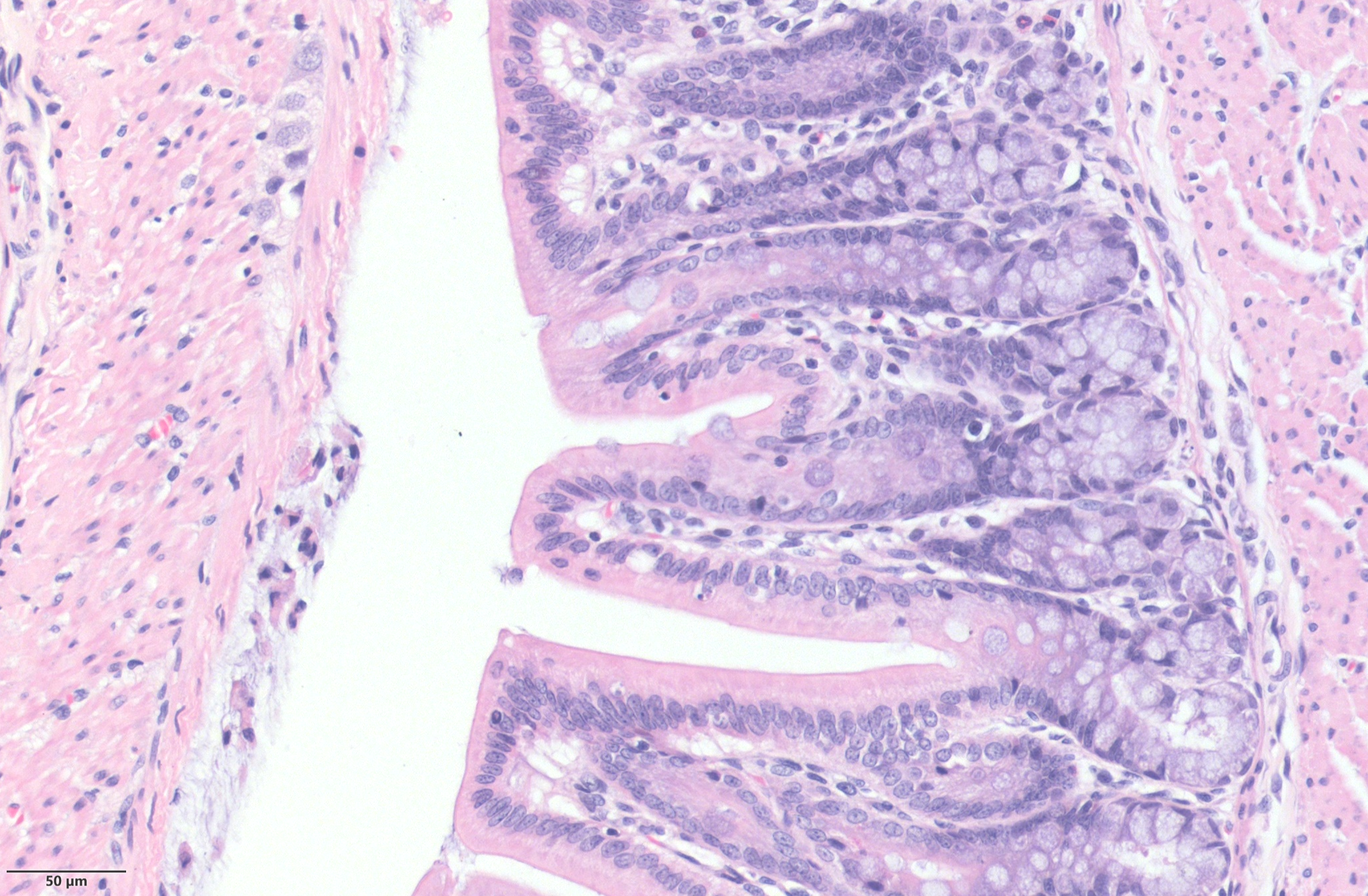

Supplement: S1 Fig — (ZIP) [file pone.0339296.s013.zip › HE/HE/Control-3-40X.tif]

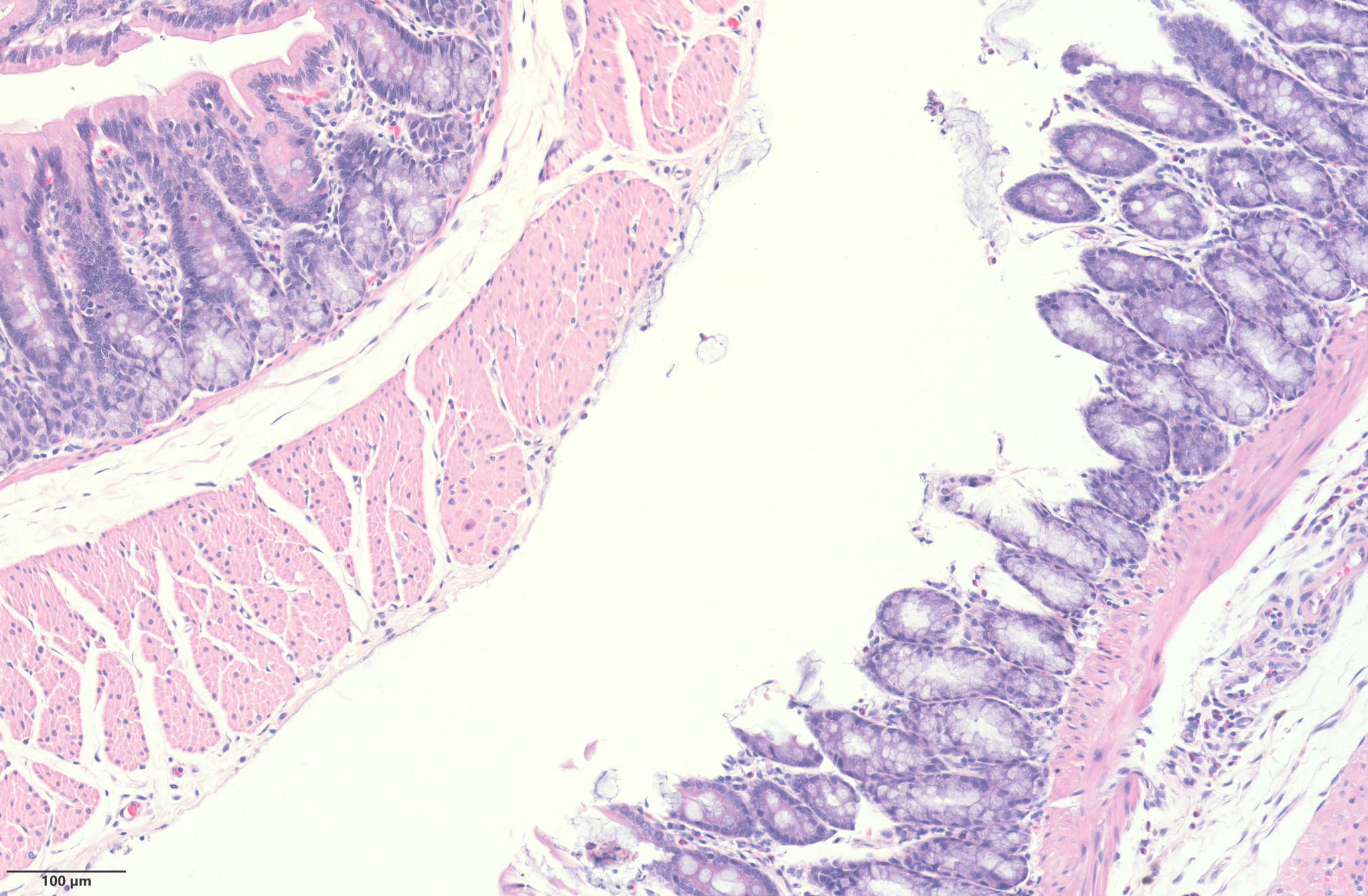

Supplement: S1 Fig — (ZIP) [file pone.0339296.s013.zip › HE/HE/Model-1-20x.tif]

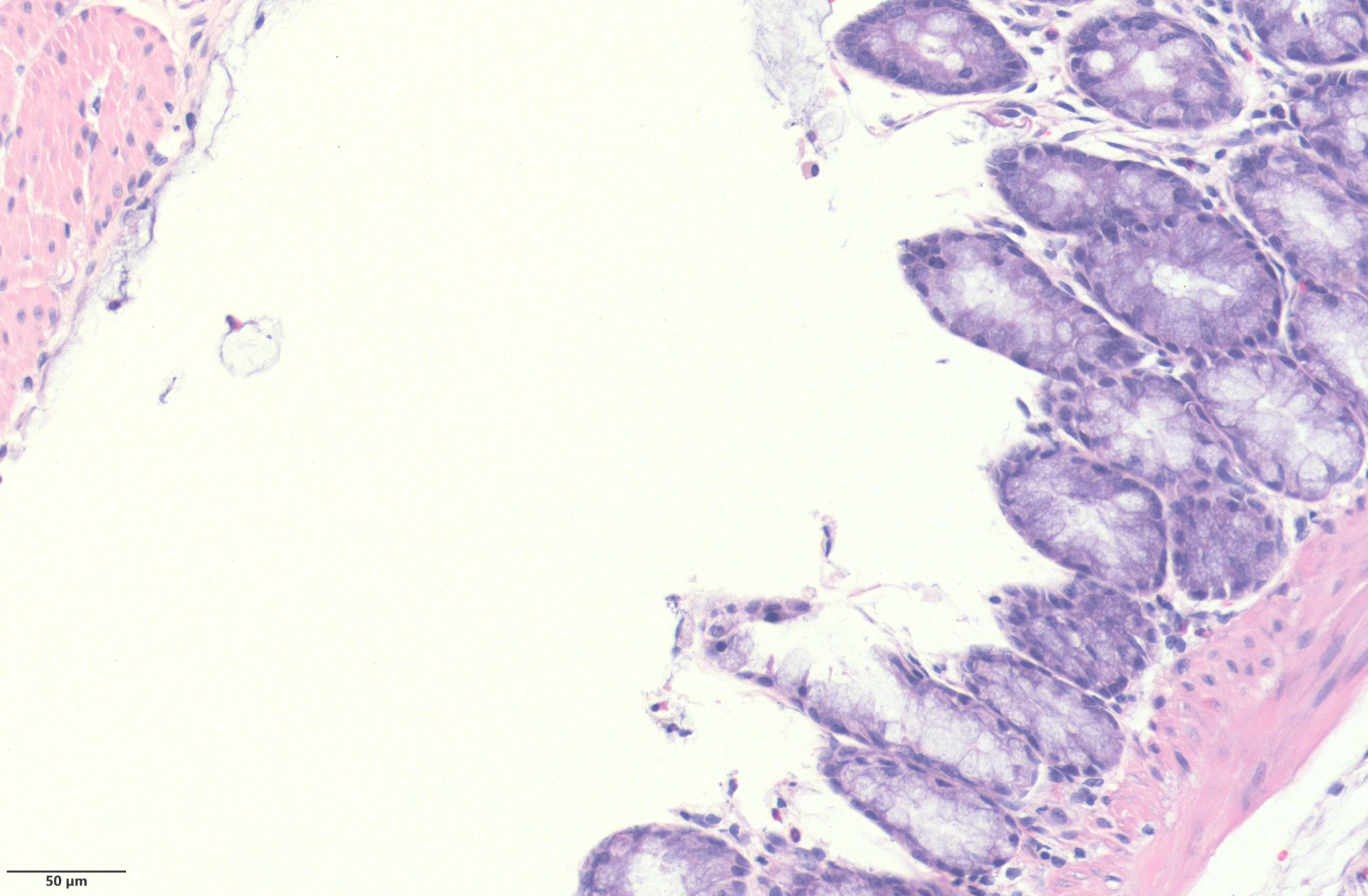

Supplement: S1 Fig — (ZIP) [file pone.0339296.s013.zip › HE/HE/Model-1-40x.tif]

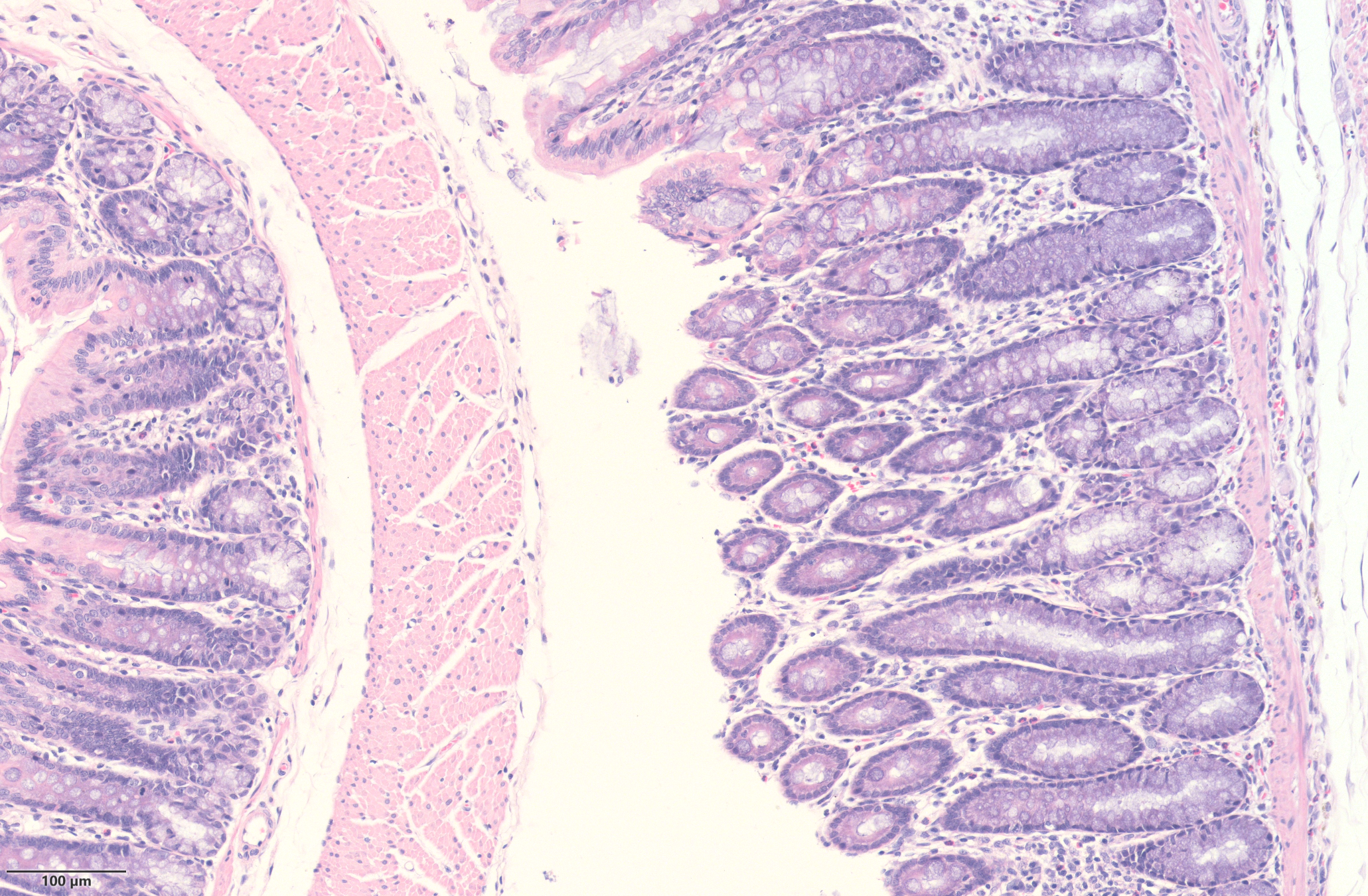

Supplement: S1 Fig — (ZIP) [file pone.0339296.s013.zip › HE/HE/Model-2-20X.tif]

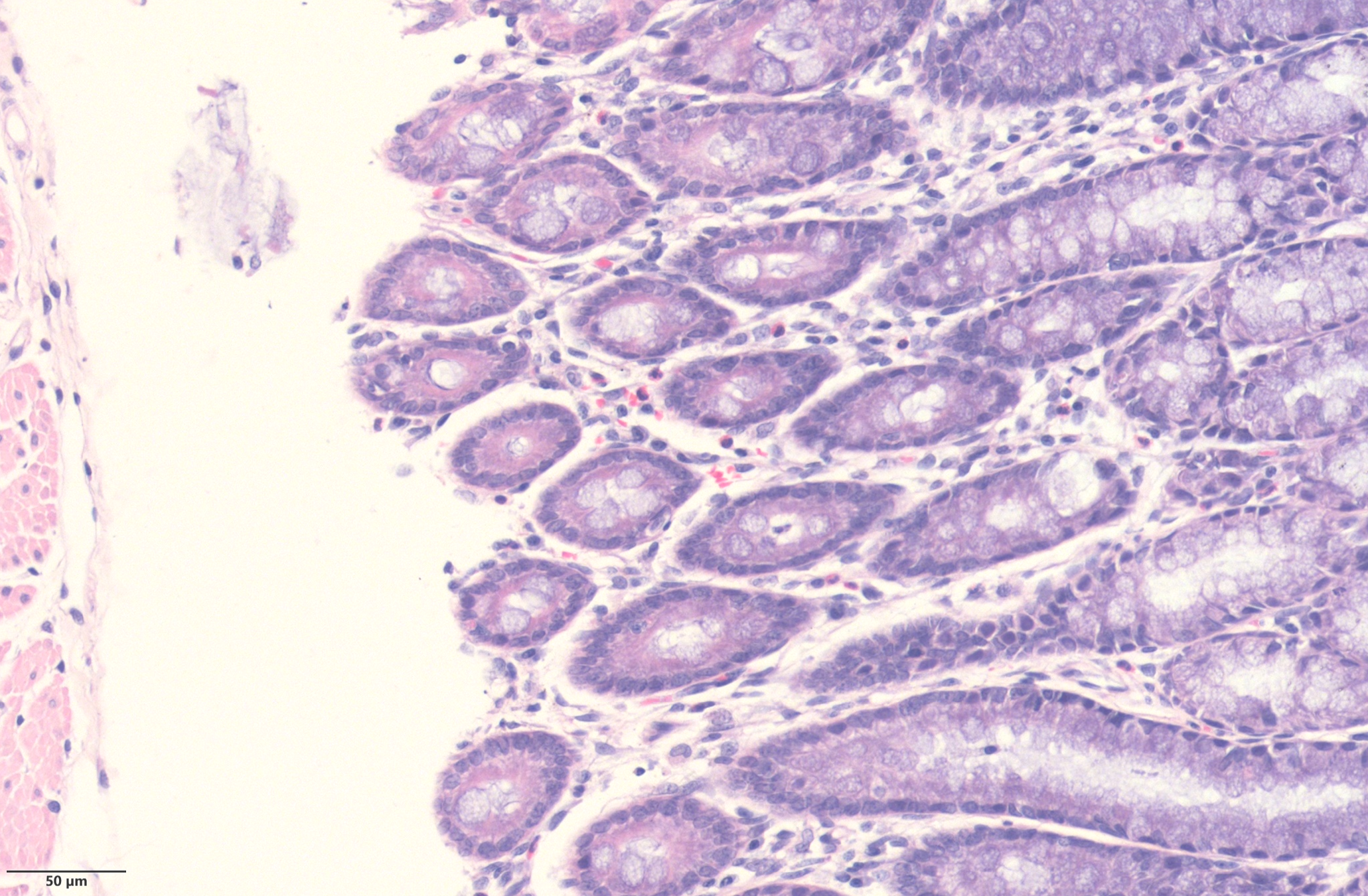

Supplement: S1 Fig — (ZIP) [file pone.0339296.s013.zip › HE/HE/Model-2-40x.tif]

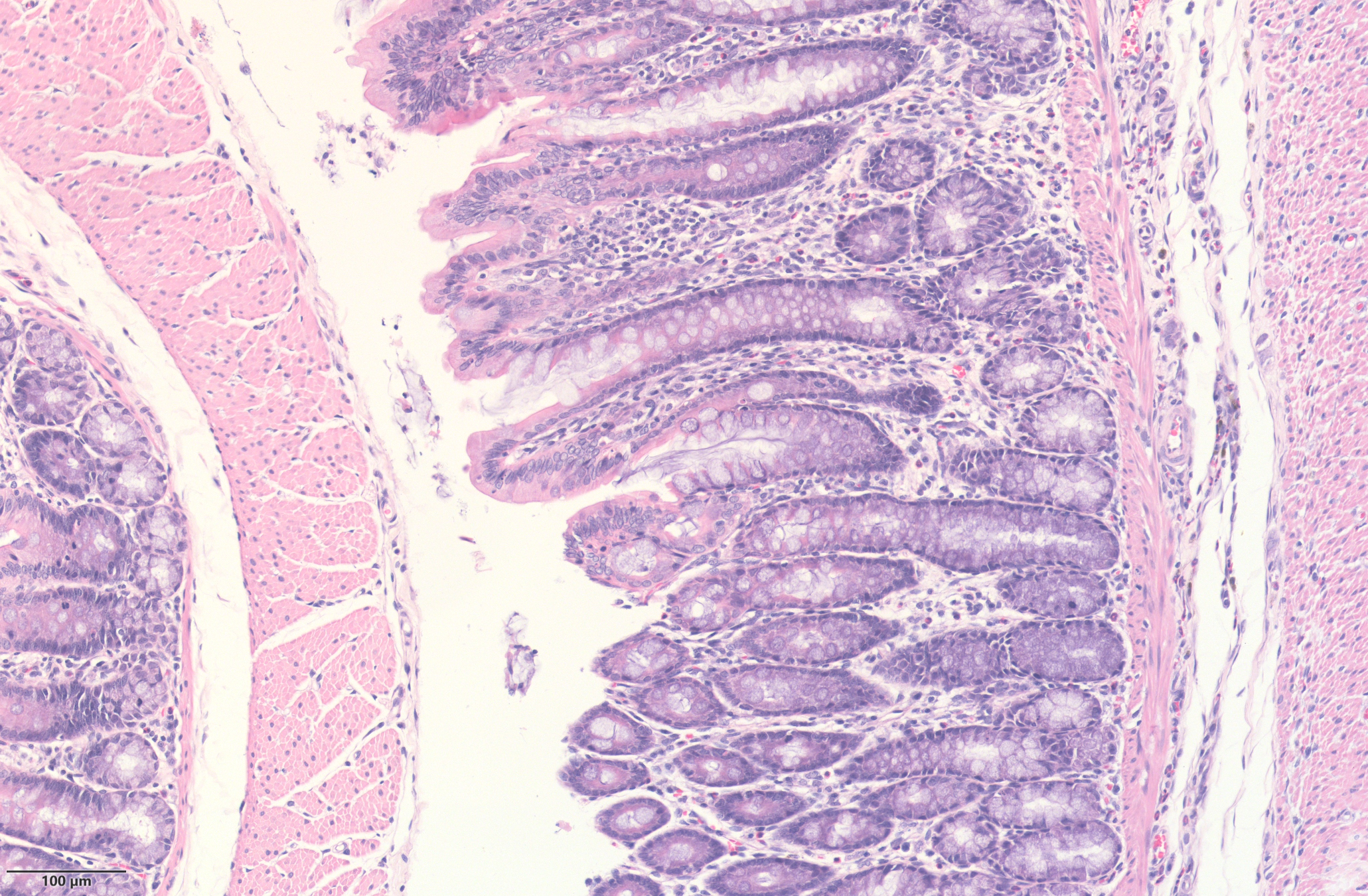

Supplement: S1 Fig — (ZIP) [file pone.0339296.s013.zip › HE/HE/Model-3-20x.tif]

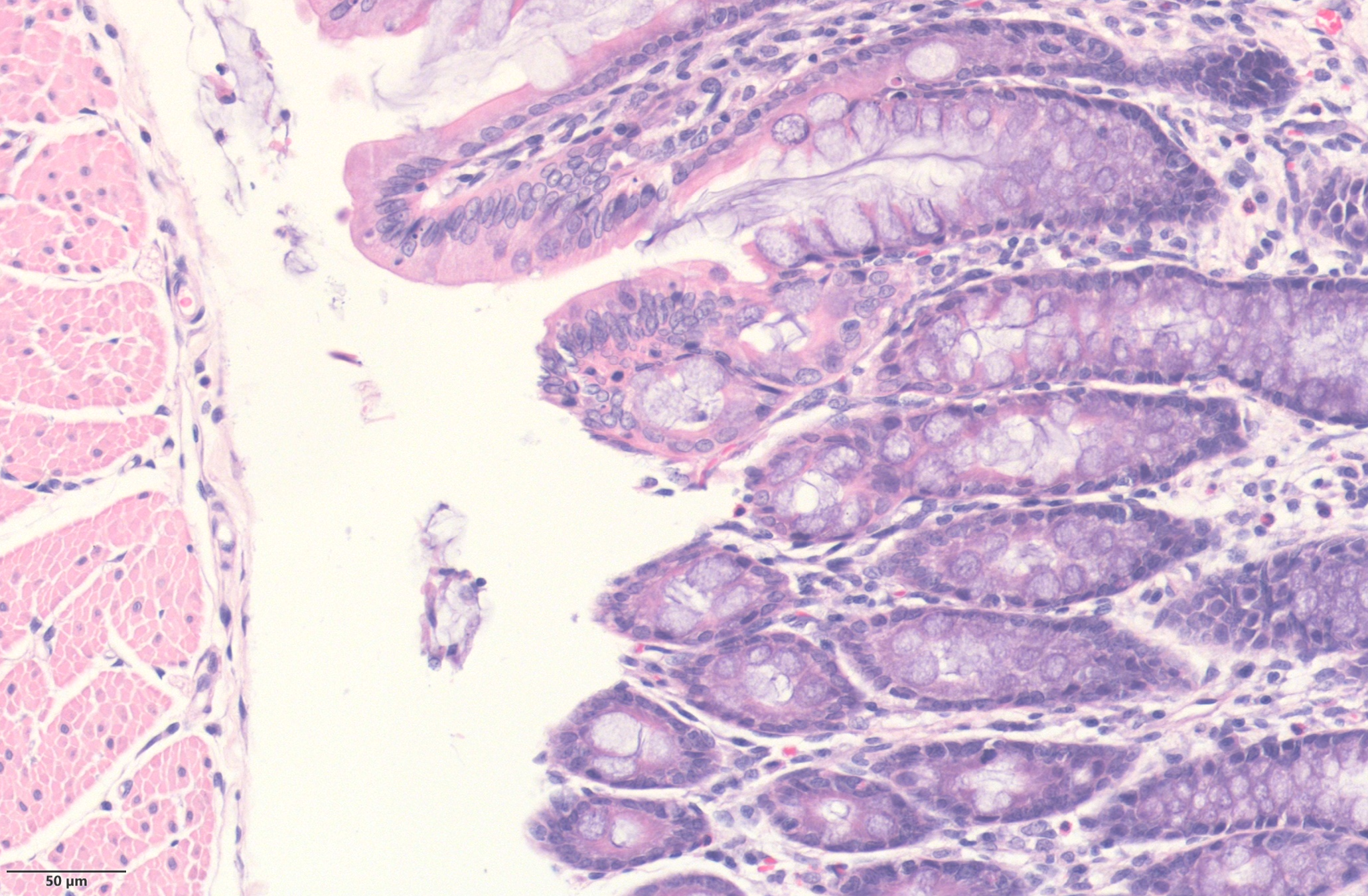

Supplement: S1 Fig — (ZIP) [file pone.0339296.s013.zip › HE/HE/Model-3-40X.tif]

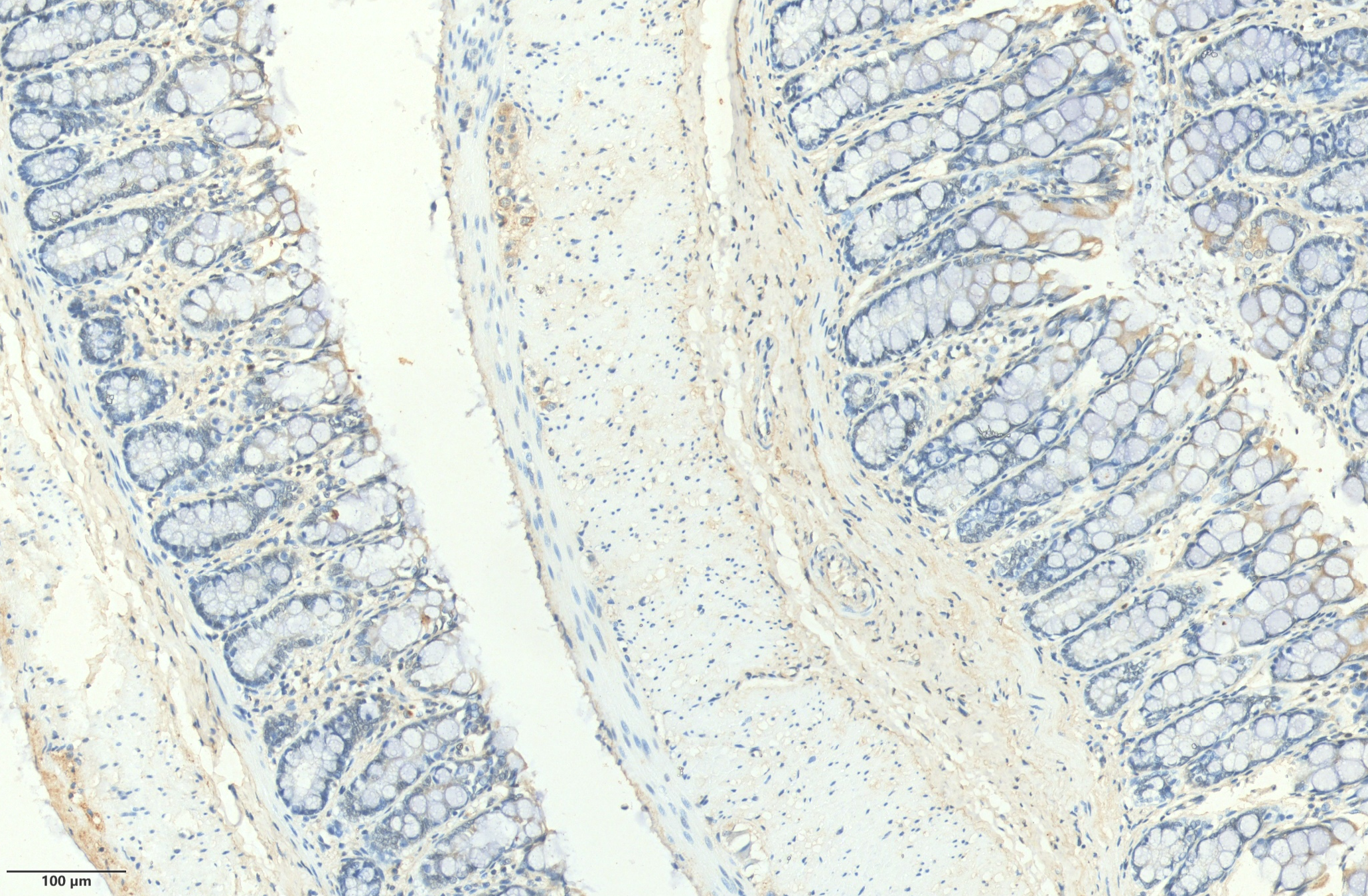

Supplement: S2 Fig — (ZIP) [file pone.0339296.s014.zip › CD55 IHC_raw_image/Control-20X-1.tif]

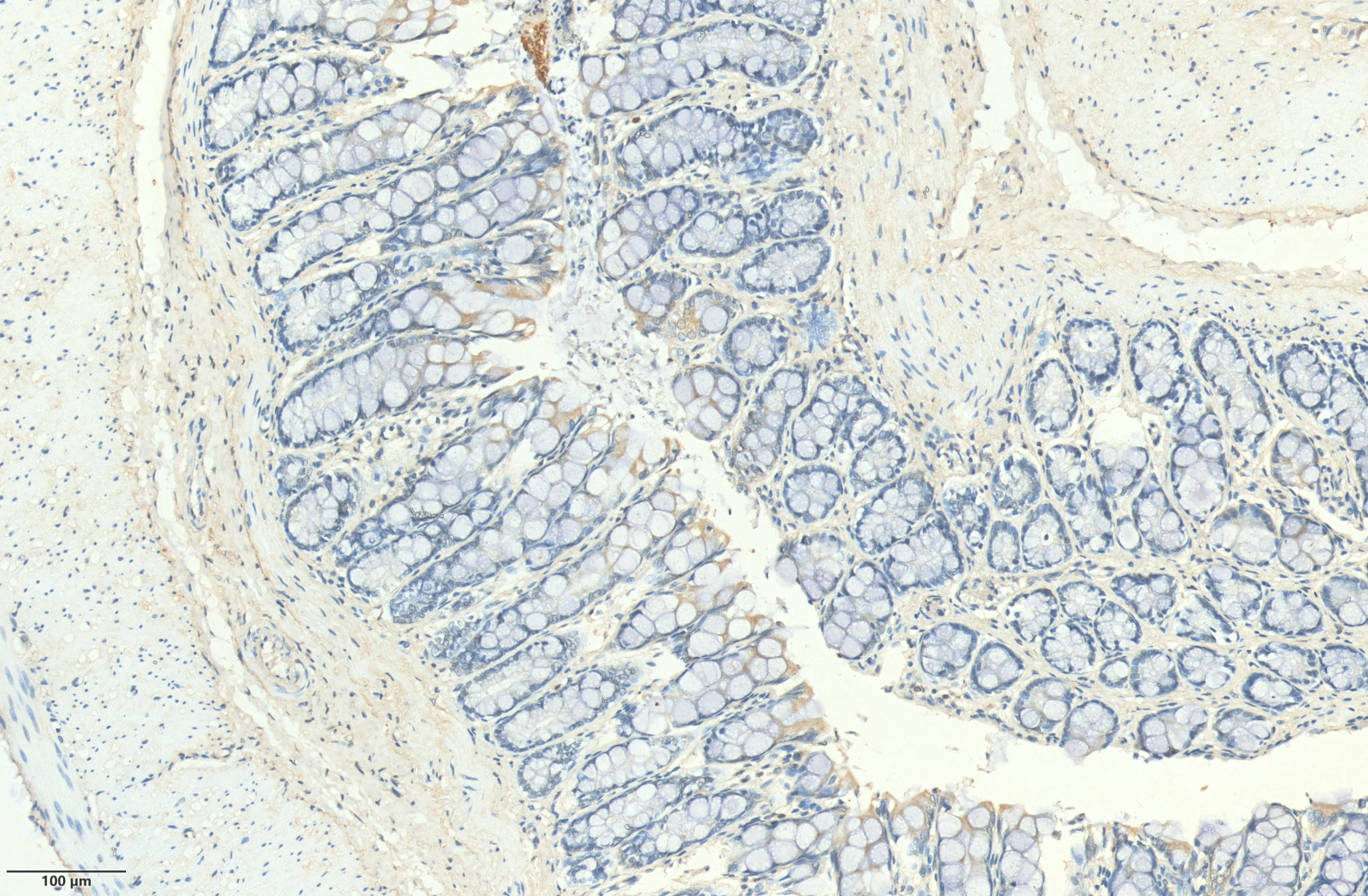

Supplement: S2 Fig — (ZIP) [file pone.0339296.s014.zip › CD55 IHC_raw_image/control20x-2.tif]

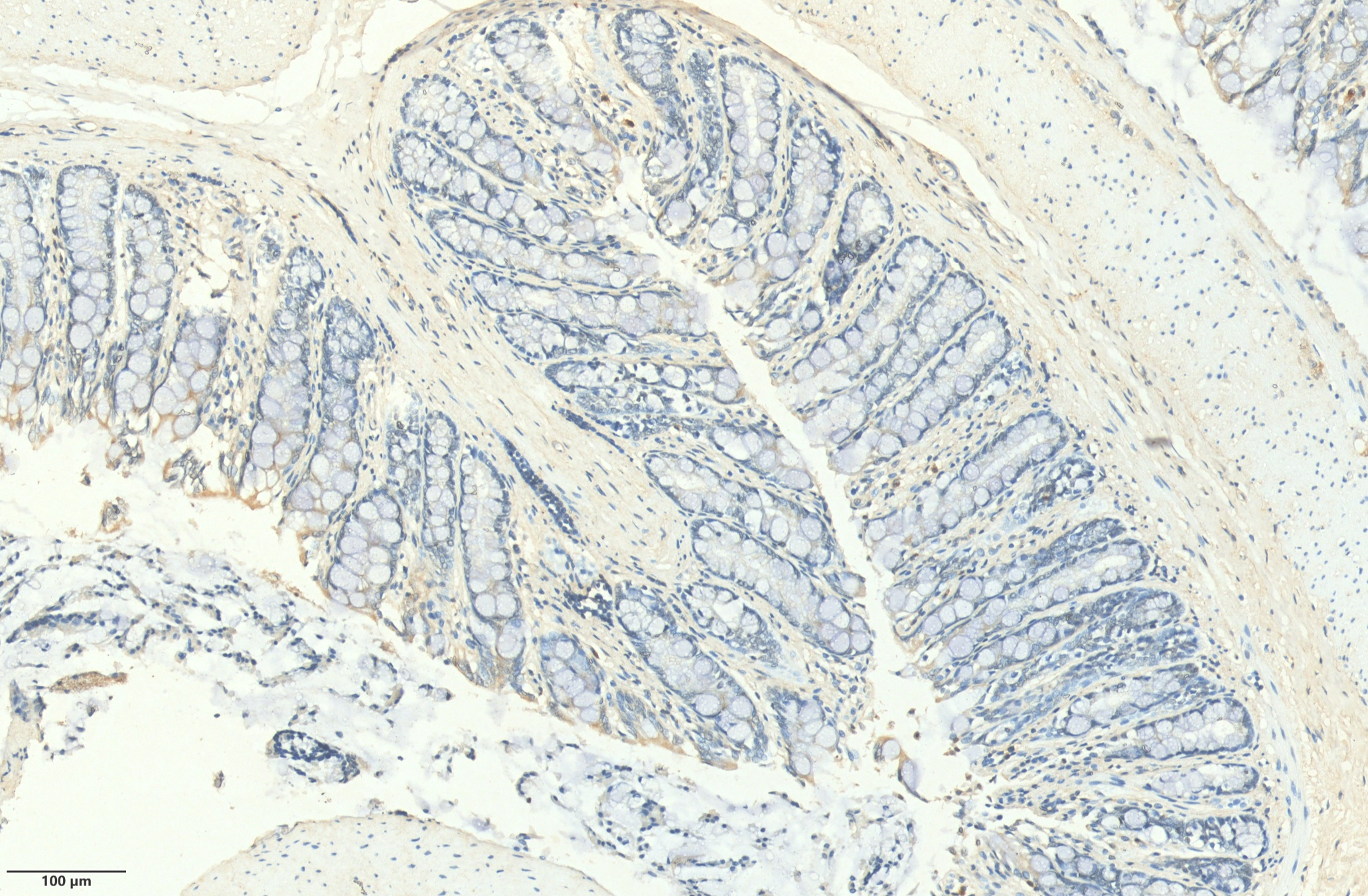

Supplement: S2 Fig — (ZIP) [file pone.0339296.s014.zip › CD55 IHC_raw_image/Control20X-3.tif]

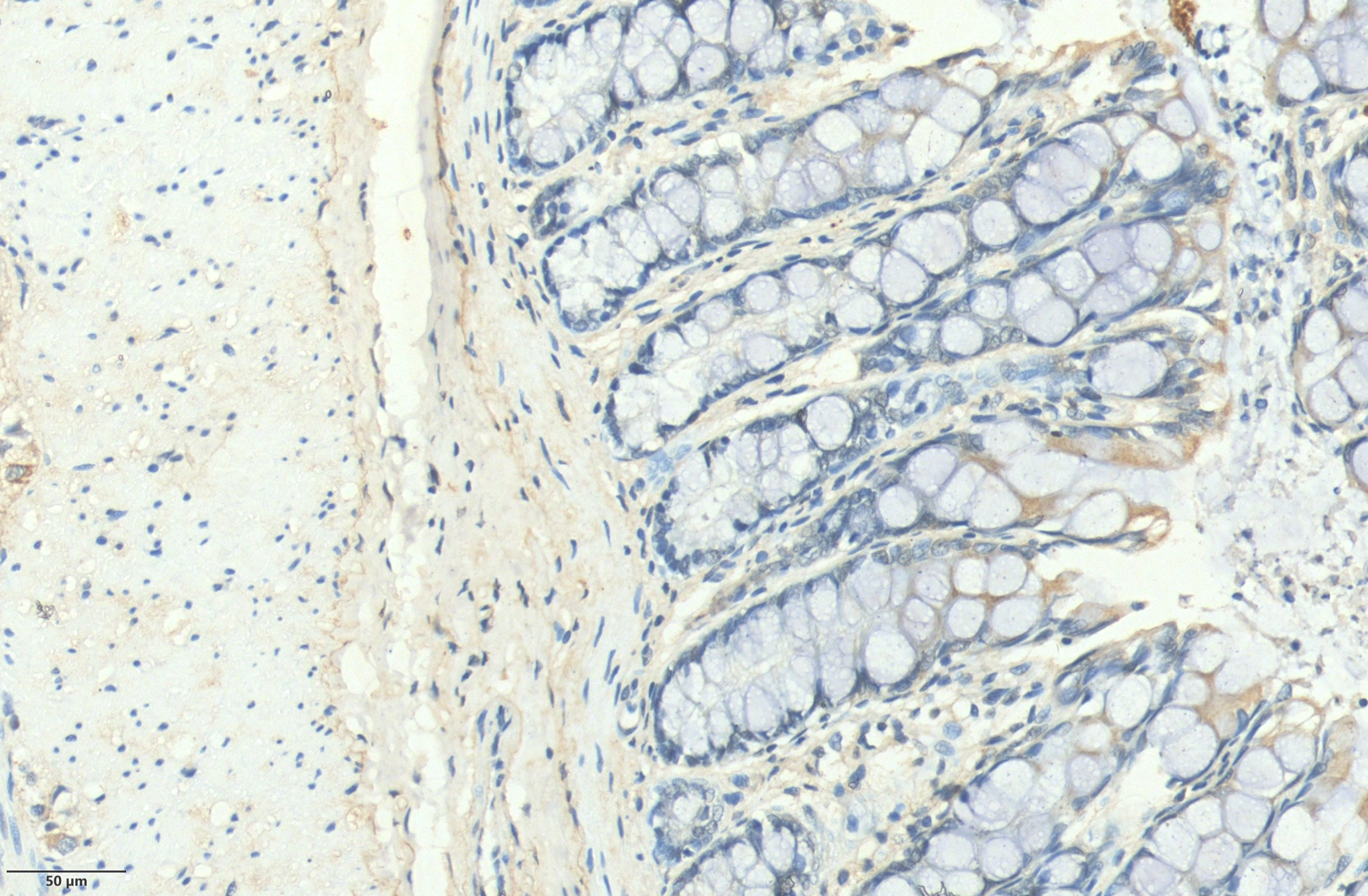

Supplement: S2 Fig — (ZIP) [file pone.0339296.s014.zip › CD55 IHC_raw_image/Control40x-1.tif]

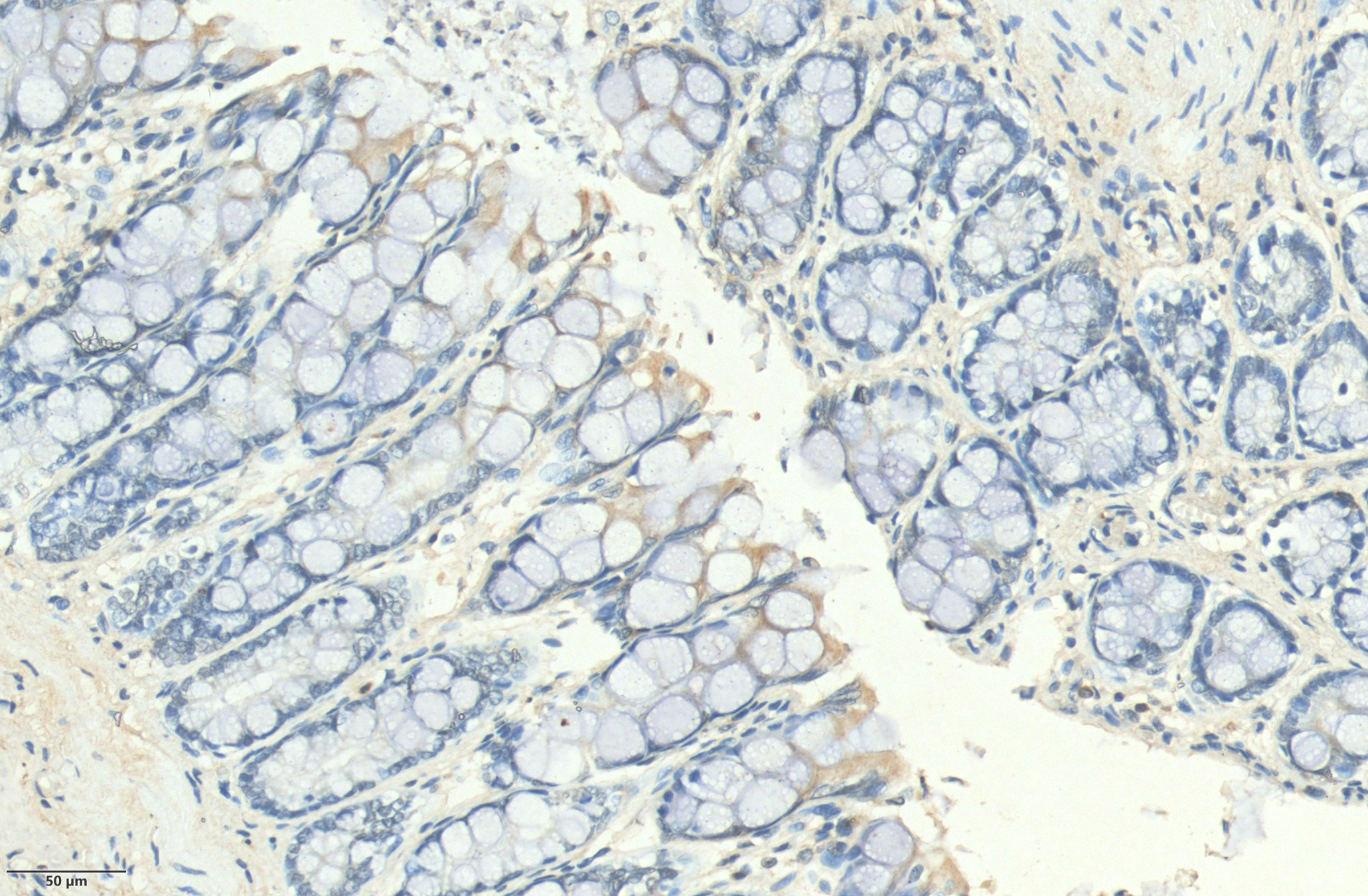

Supplement: S2 Fig — (ZIP) [file pone.0339296.s014.zip › CD55 IHC_raw_image/control40X-2.tif]

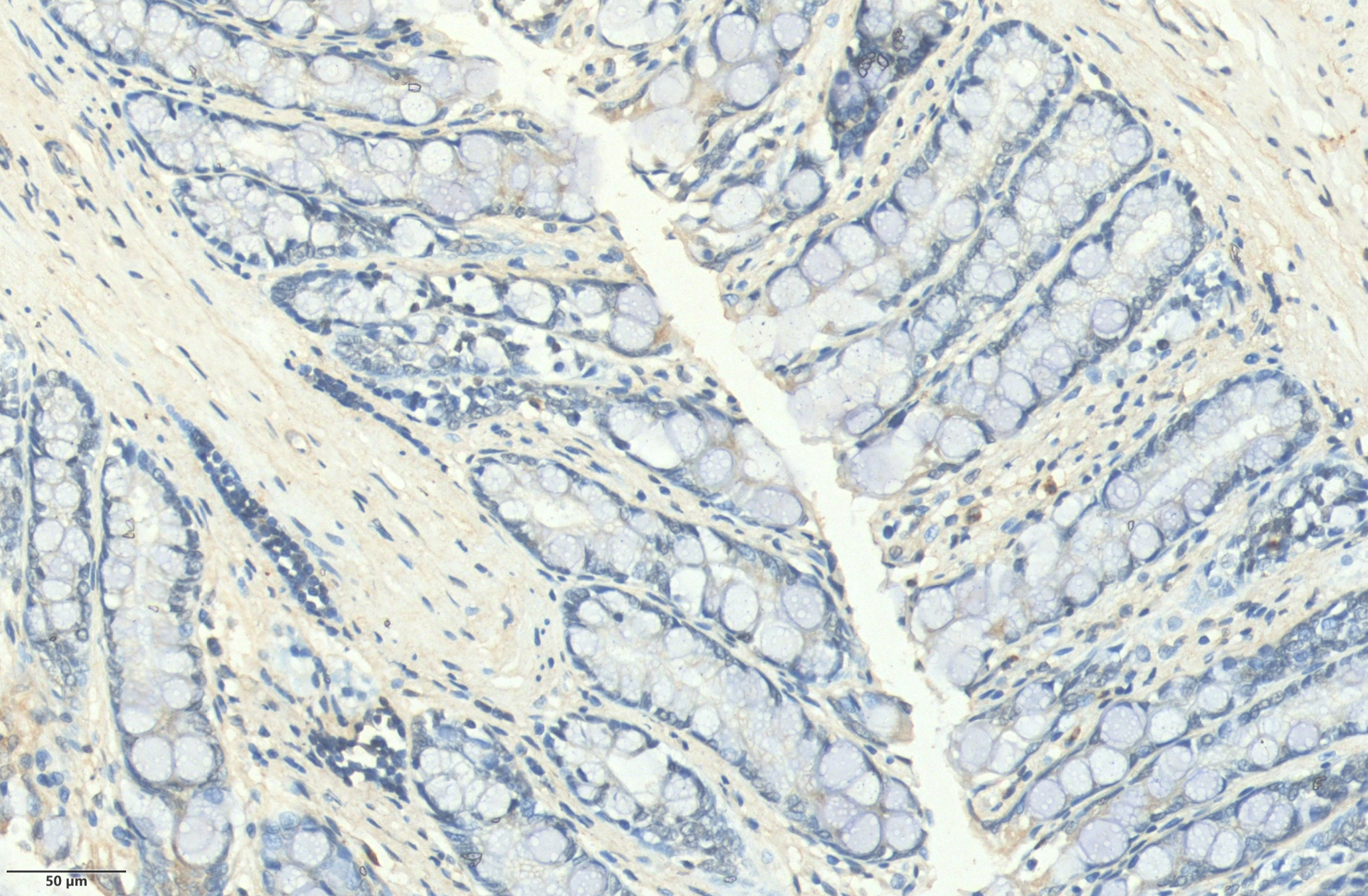

Supplement: S2 Fig — (ZIP) [file pone.0339296.s014.zip › CD55 IHC_raw_image/Control40X-3.tif]

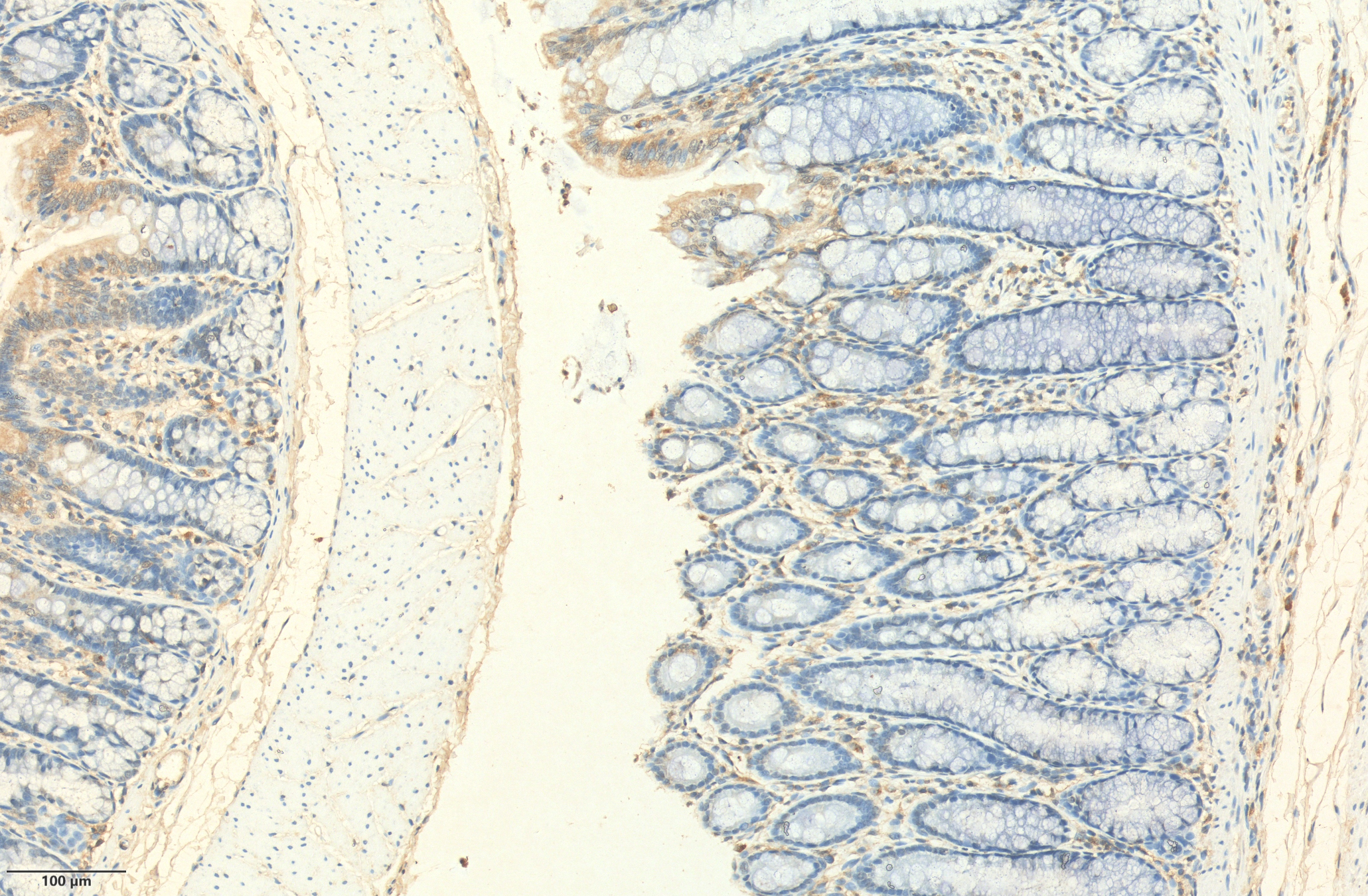

Supplement: S2 Fig — (ZIP) [file pone.0339296.s014.zip › CD55 IHC_raw_image/Model20x-1.tif]

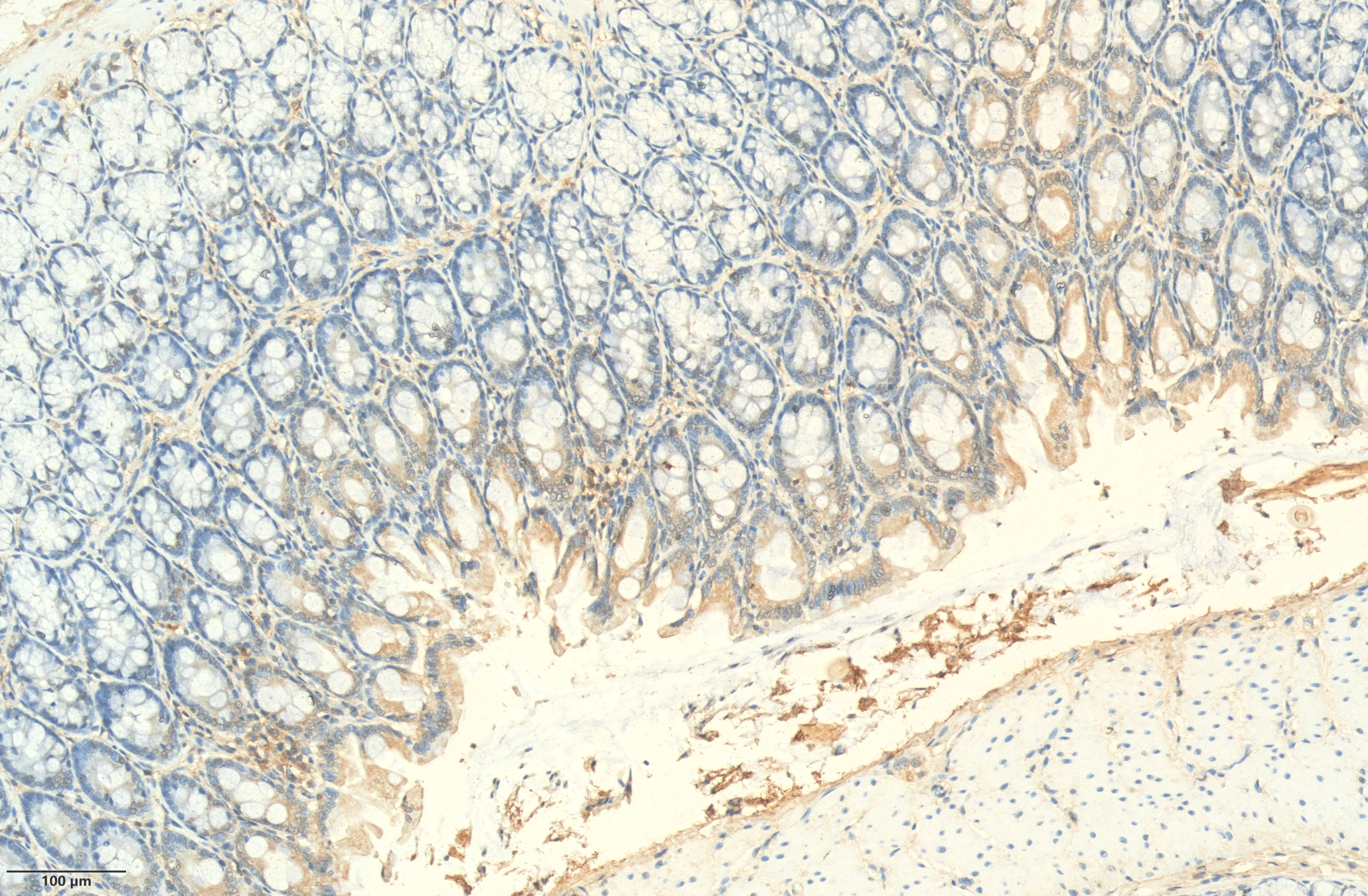

Supplement: S2 Fig — (ZIP) [file pone.0339296.s014.zip › CD55 IHC_raw_image/model20x-2.tif]

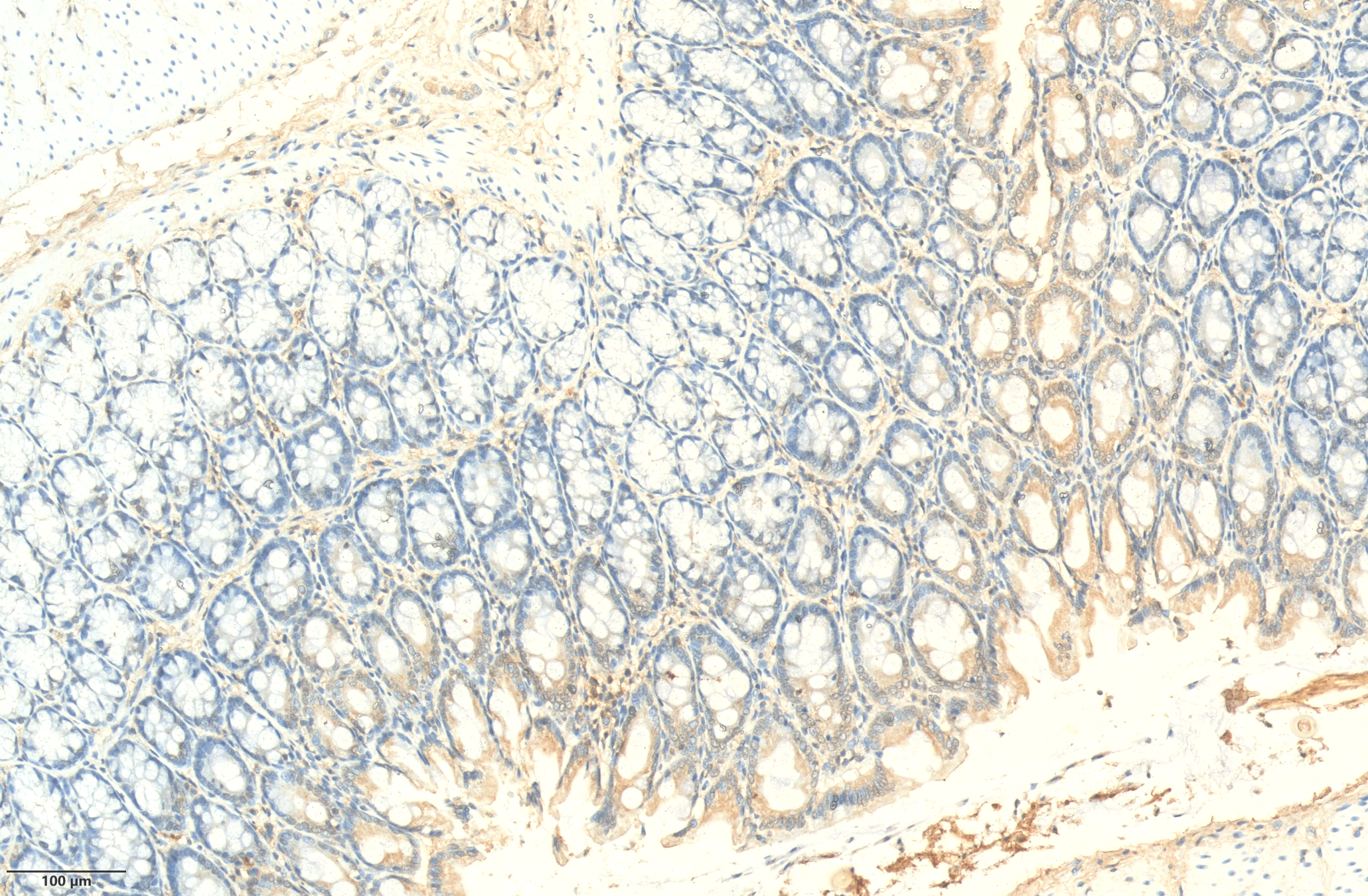

Supplement: S2 Fig — (ZIP) [file pone.0339296.s014.zip › CD55 IHC_raw_image/model20X-3.tif]

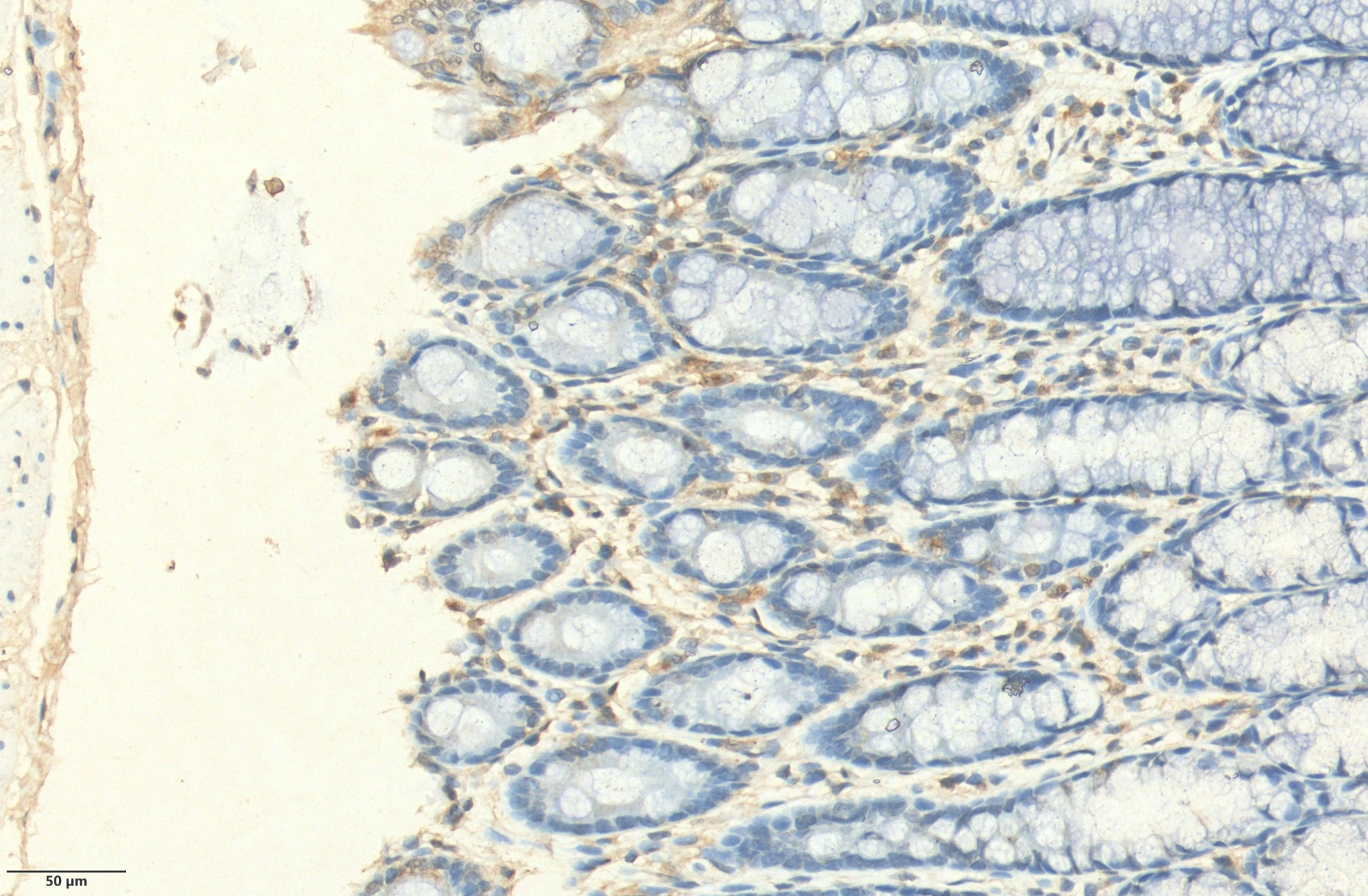

Supplement: S2 Fig — (ZIP) [file pone.0339296.s014.zip › CD55 IHC_raw_image/model40x-1.tif]

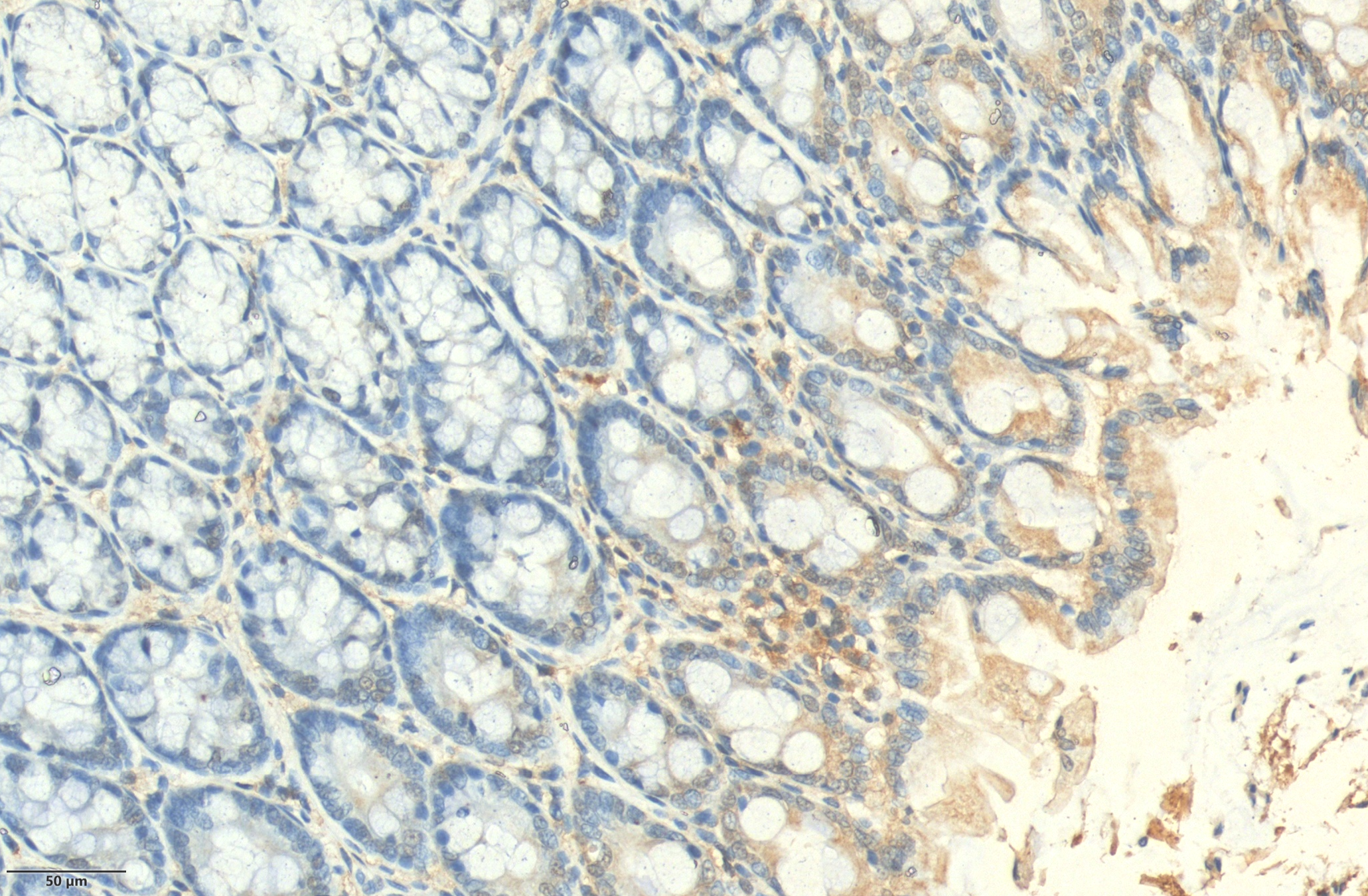

Supplement: S2 Fig — (ZIP) [file pone.0339296.s014.zip › CD55 IHC_raw_image/model40x-2.tif]

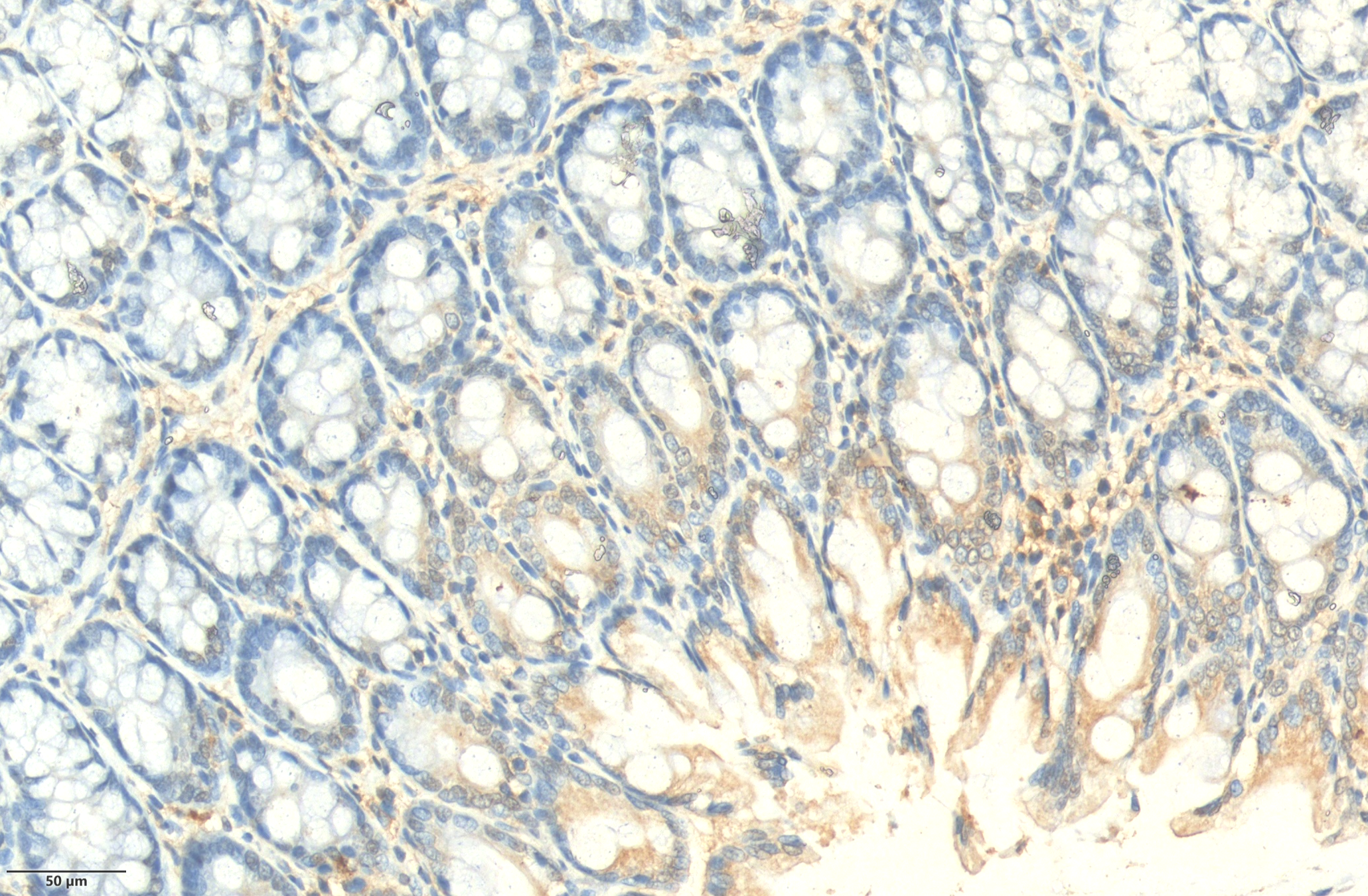

Supplement: S2 Fig — (ZIP) [file pone.0339296.s014.zip › CD55 IHC_raw_image/model40x-3.tif]

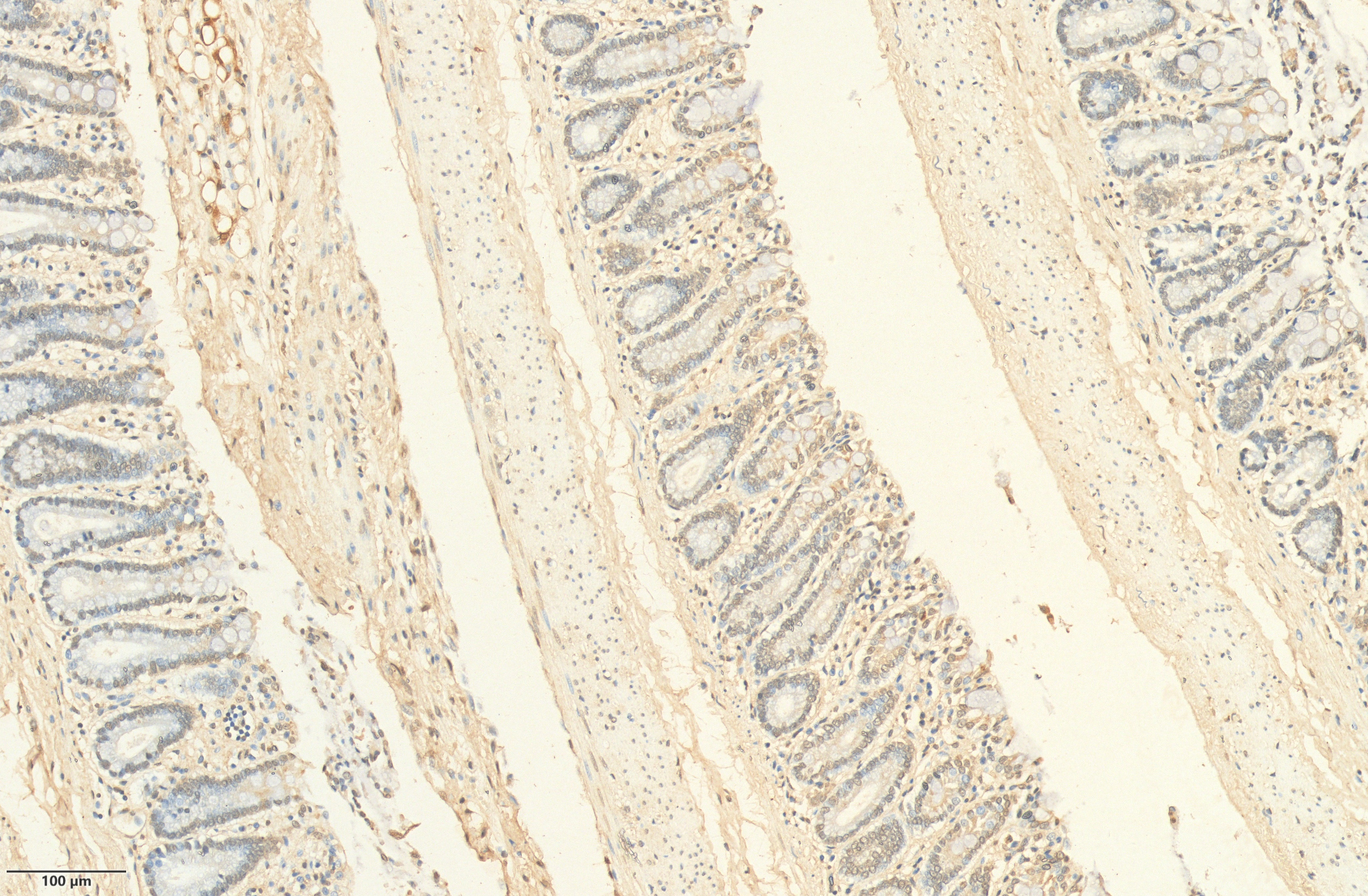

Supplement: S3 Fig — (ZIP) [file pone.0339296.s015.zip › CPT1A IHC_raw_image/control-1-20x.tif]

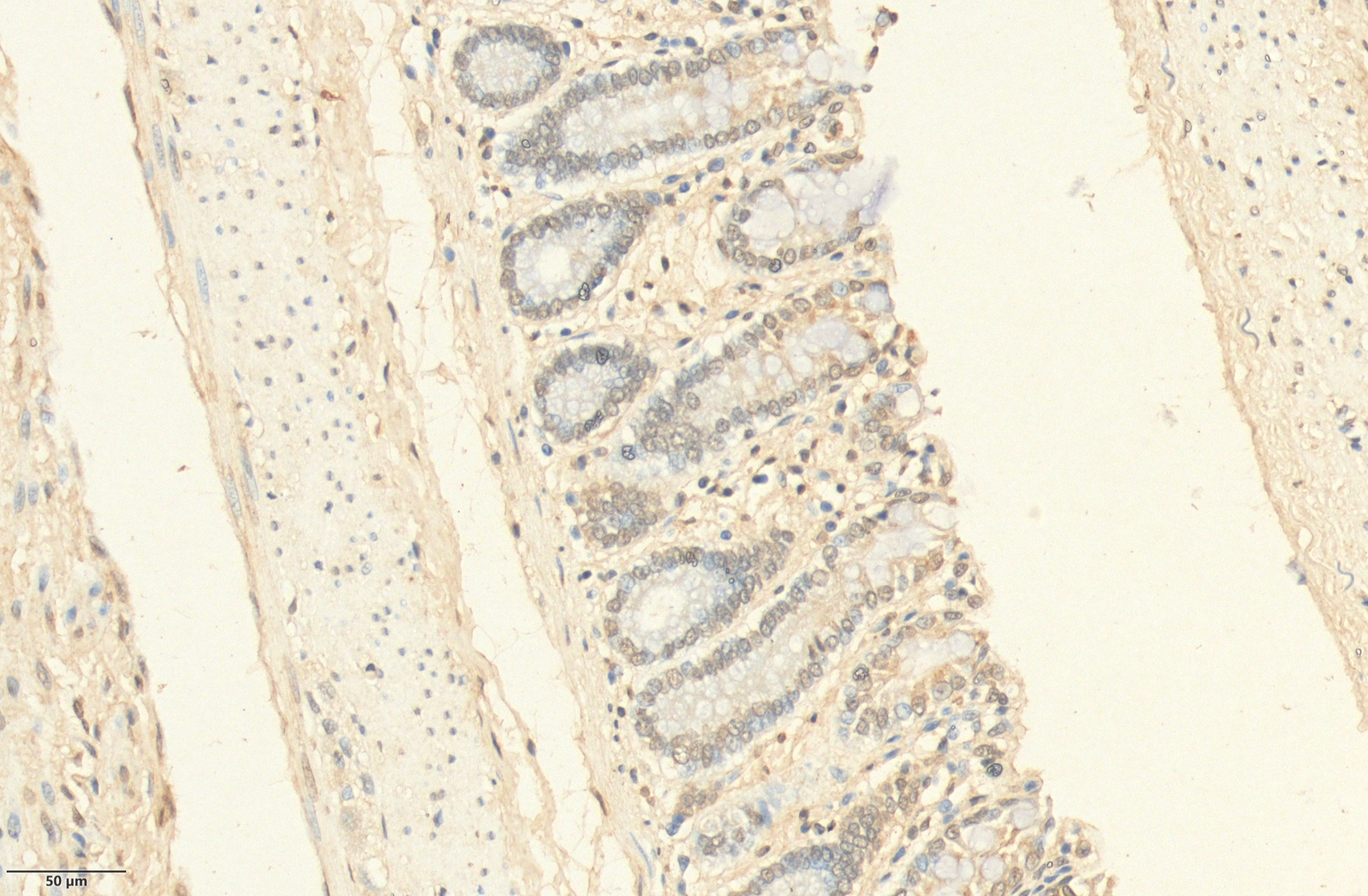

Supplement: S3 Fig — (ZIP) [file pone.0339296.s015.zip › CPT1A IHC_raw_image/control-1-40X.tif]

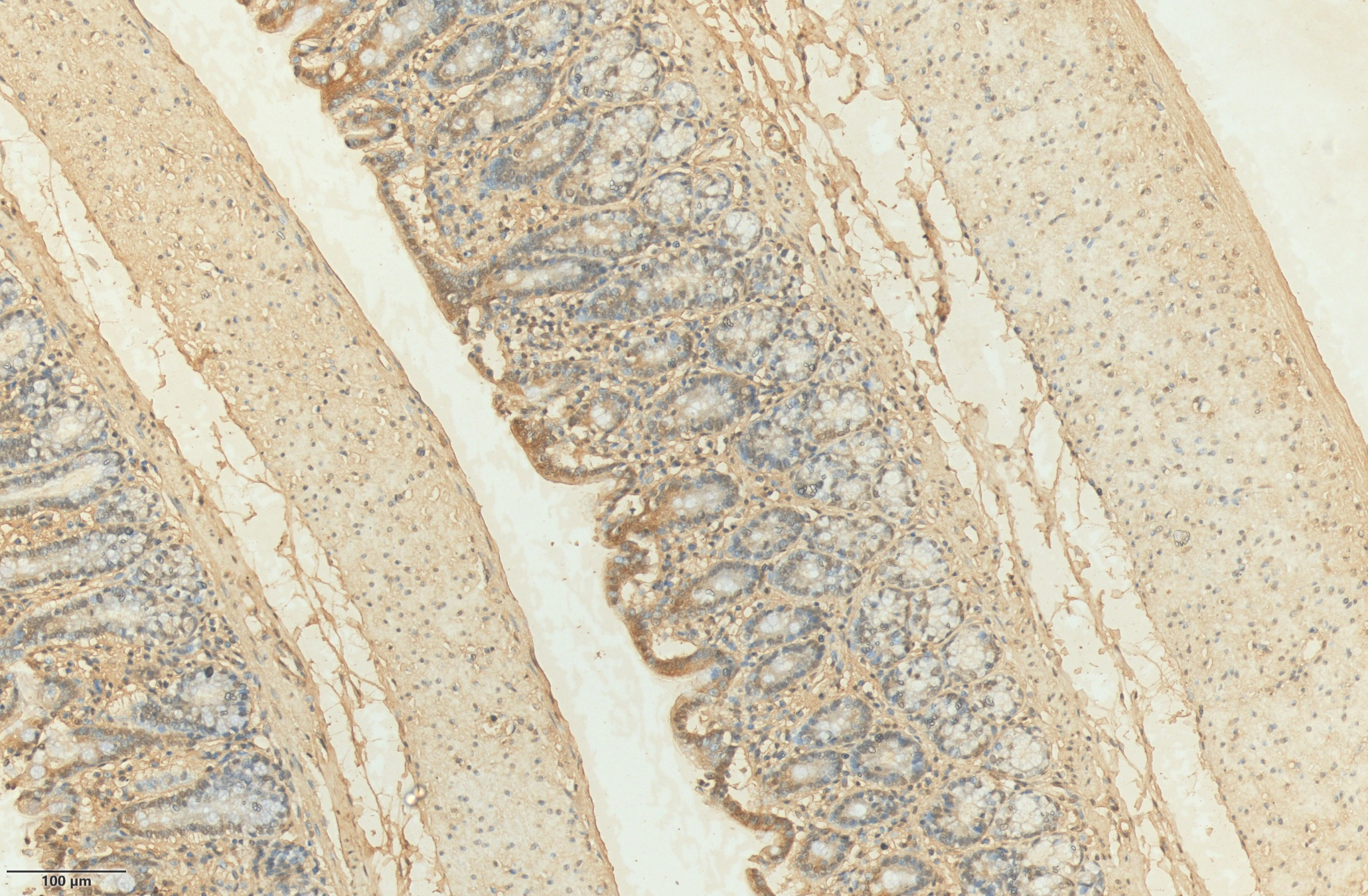

Supplement: S3 Fig — (ZIP) [file pone.0339296.s015.zip › CPT1A IHC_raw_image/Control-2-20x.tif]

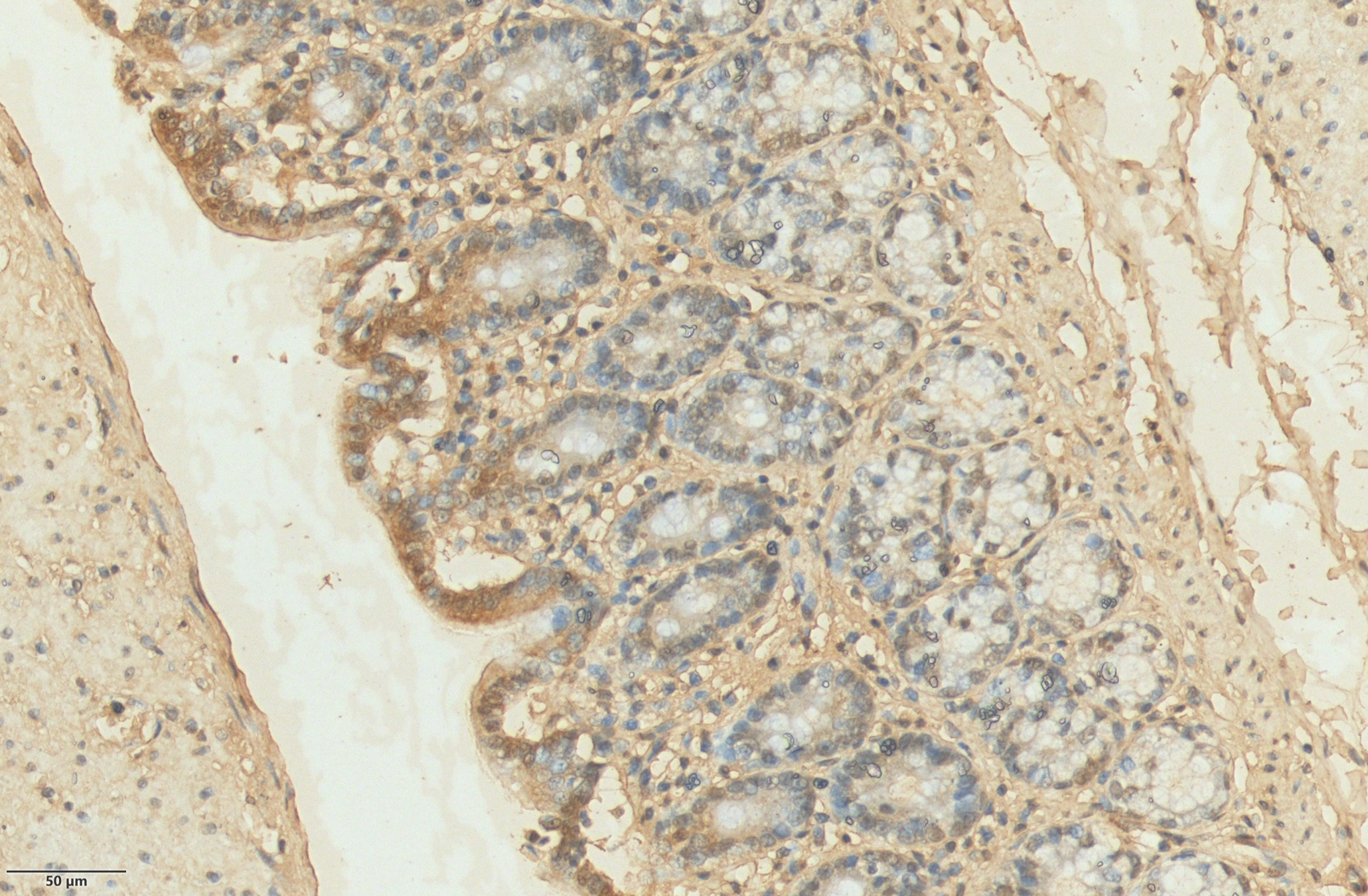

Supplement: S3 Fig — (ZIP) [file pone.0339296.s015.zip › CPT1A IHC_raw_image/Control-2-40x.tif]

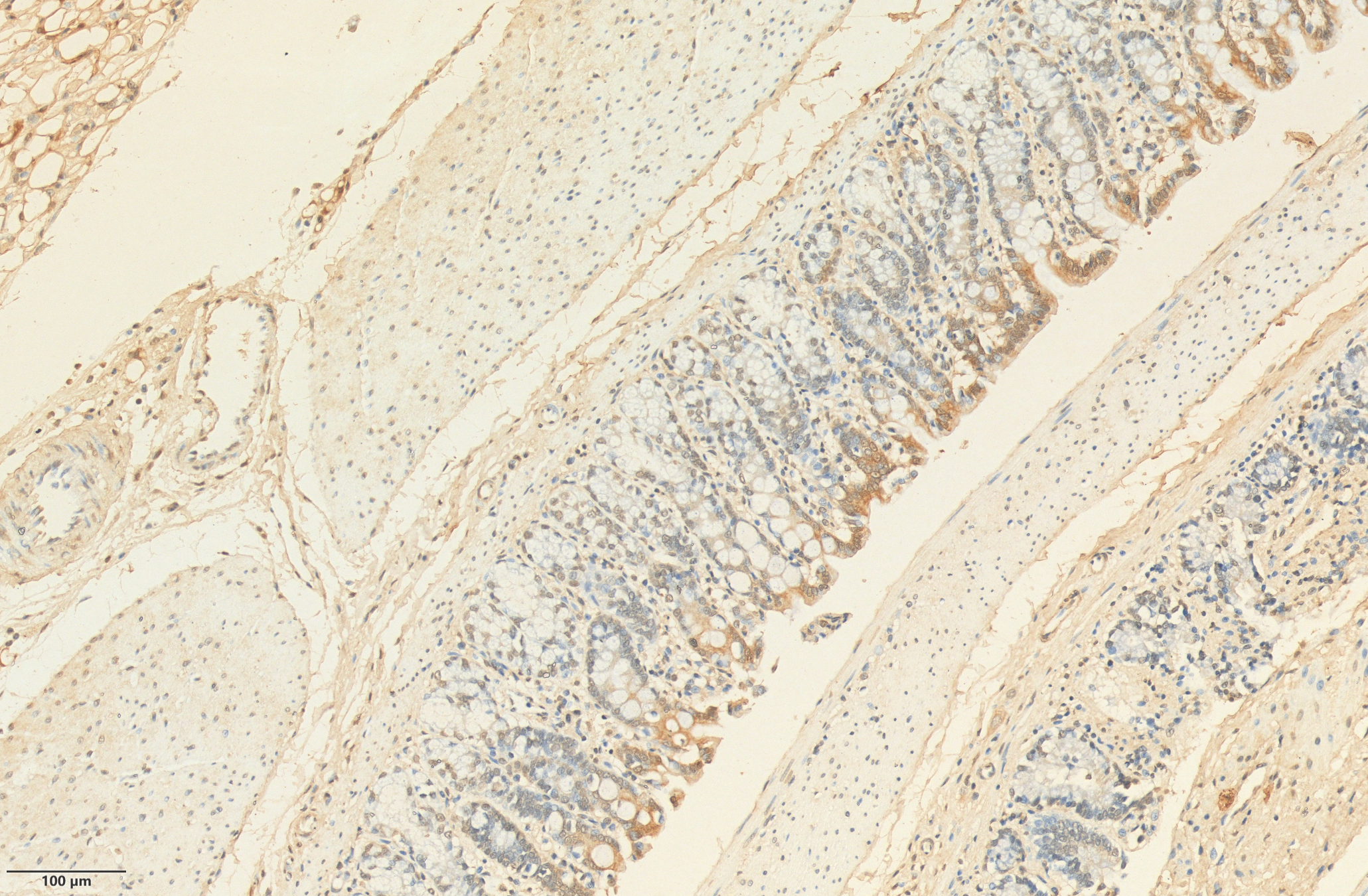

Supplement: S3 Fig — (ZIP) [file pone.0339296.s015.zip › CPT1A IHC_raw_image/Control-3-20X.tif]

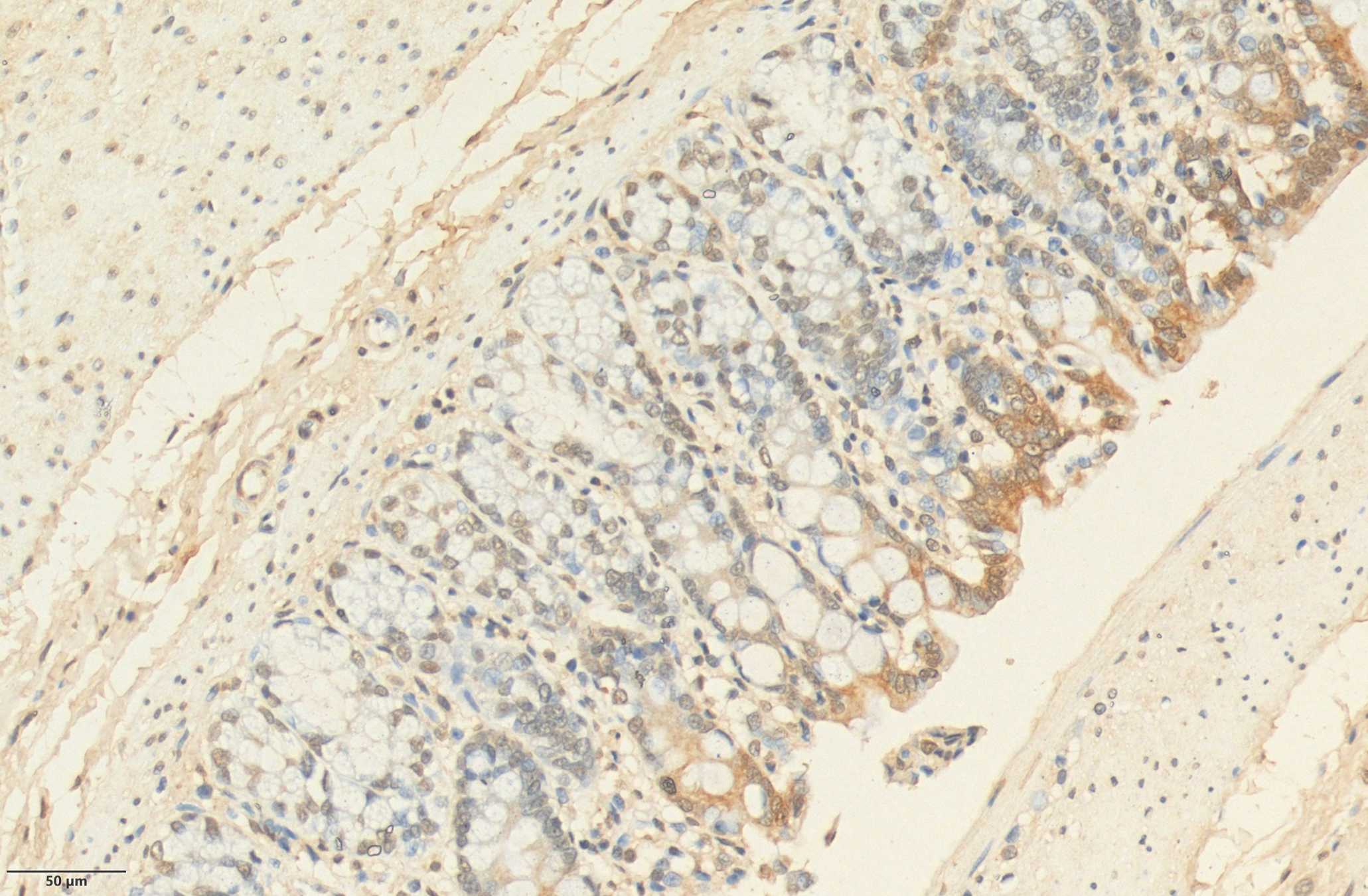

Supplement: S3 Fig — (ZIP) [file pone.0339296.s015.zip › CPT1A IHC_raw_image/Control-3-40x.tif]

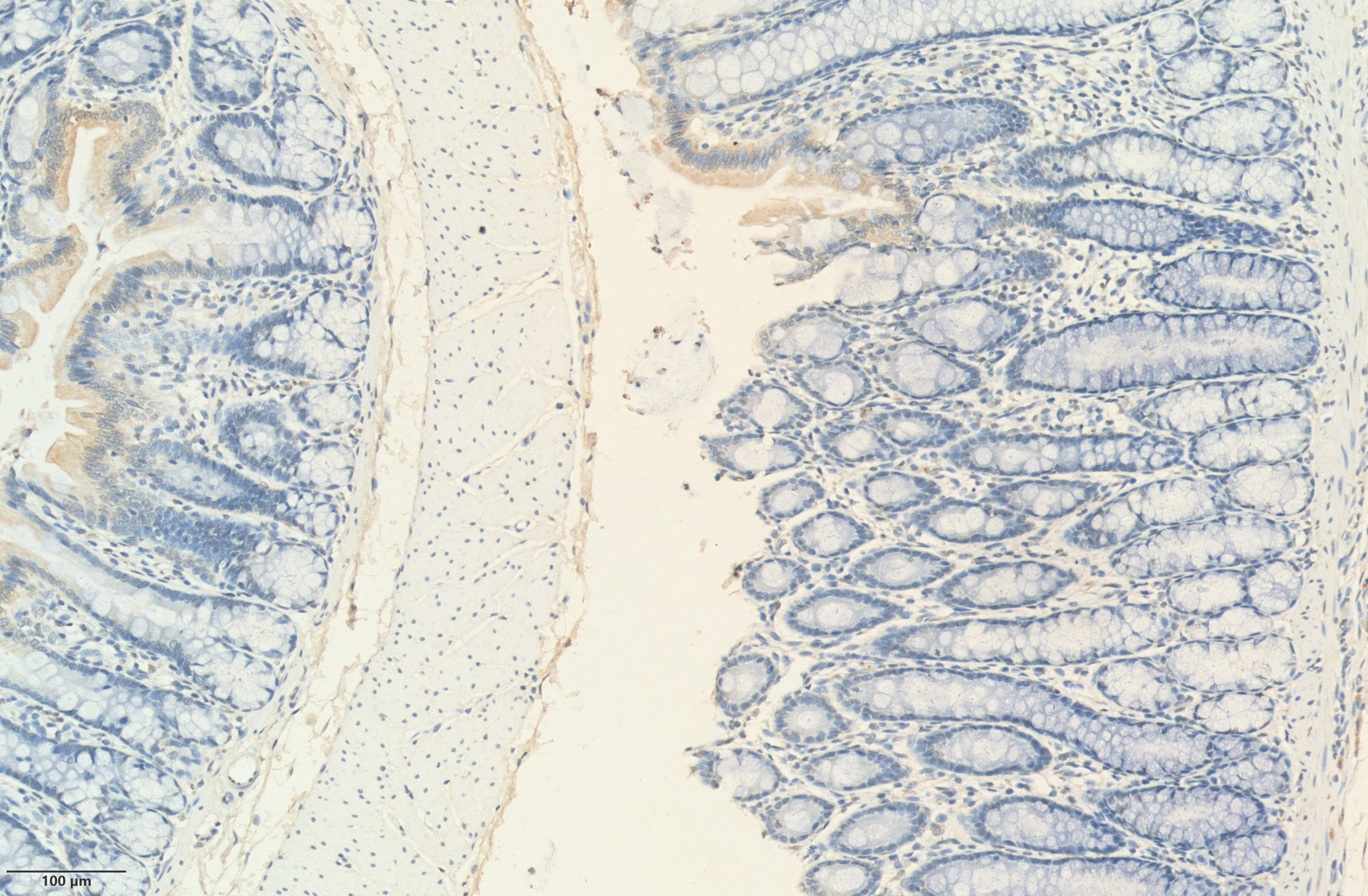

Supplement: S3 Fig — (ZIP) [file pone.0339296.s015.zip › CPT1A IHC_raw_image/MODEL-1-20X.tif]

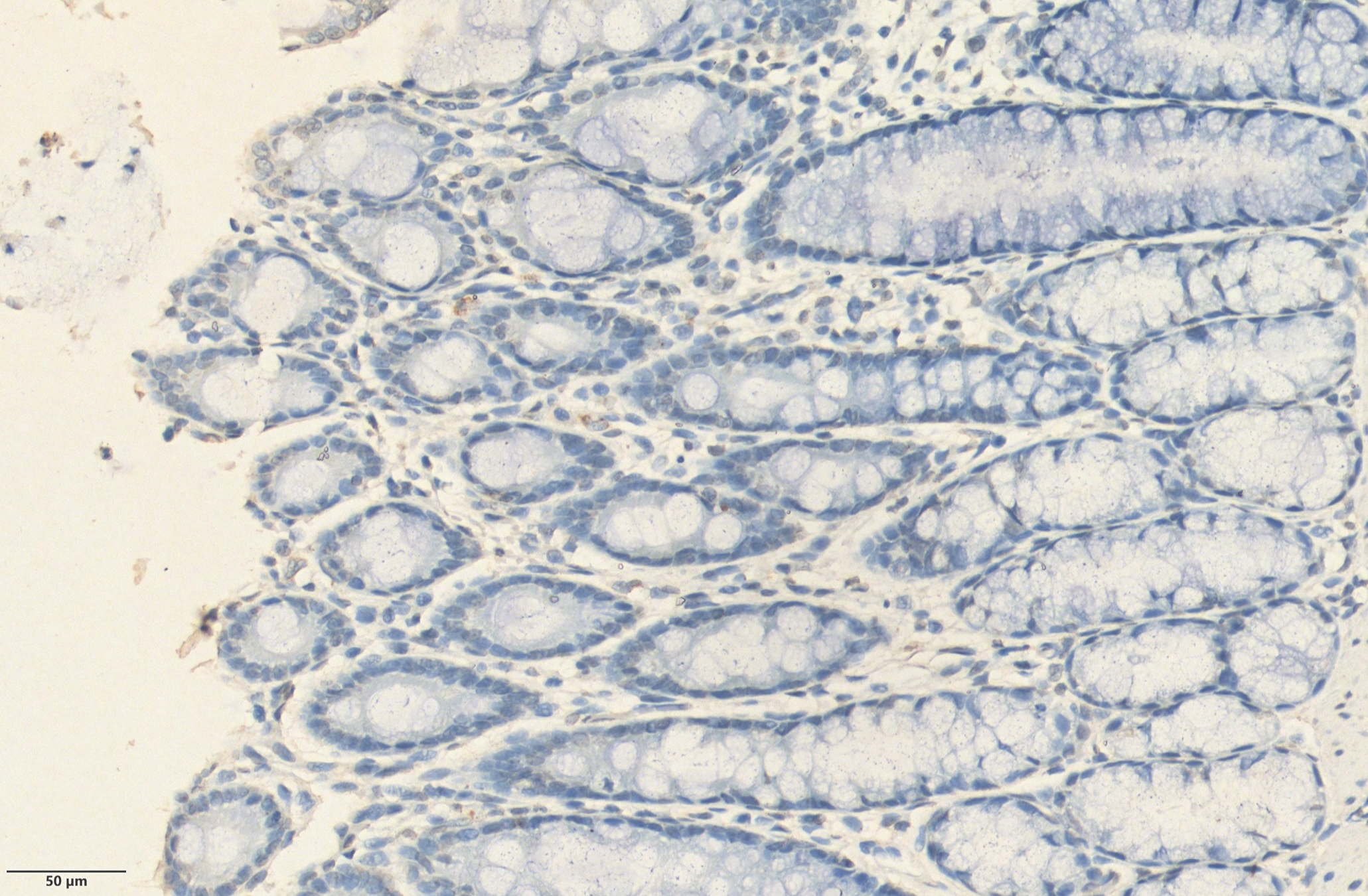

Supplement: S3 Fig — (ZIP) [file pone.0339296.s015.zip › CPT1A IHC_raw_image/MODEL-1-40X.tif]

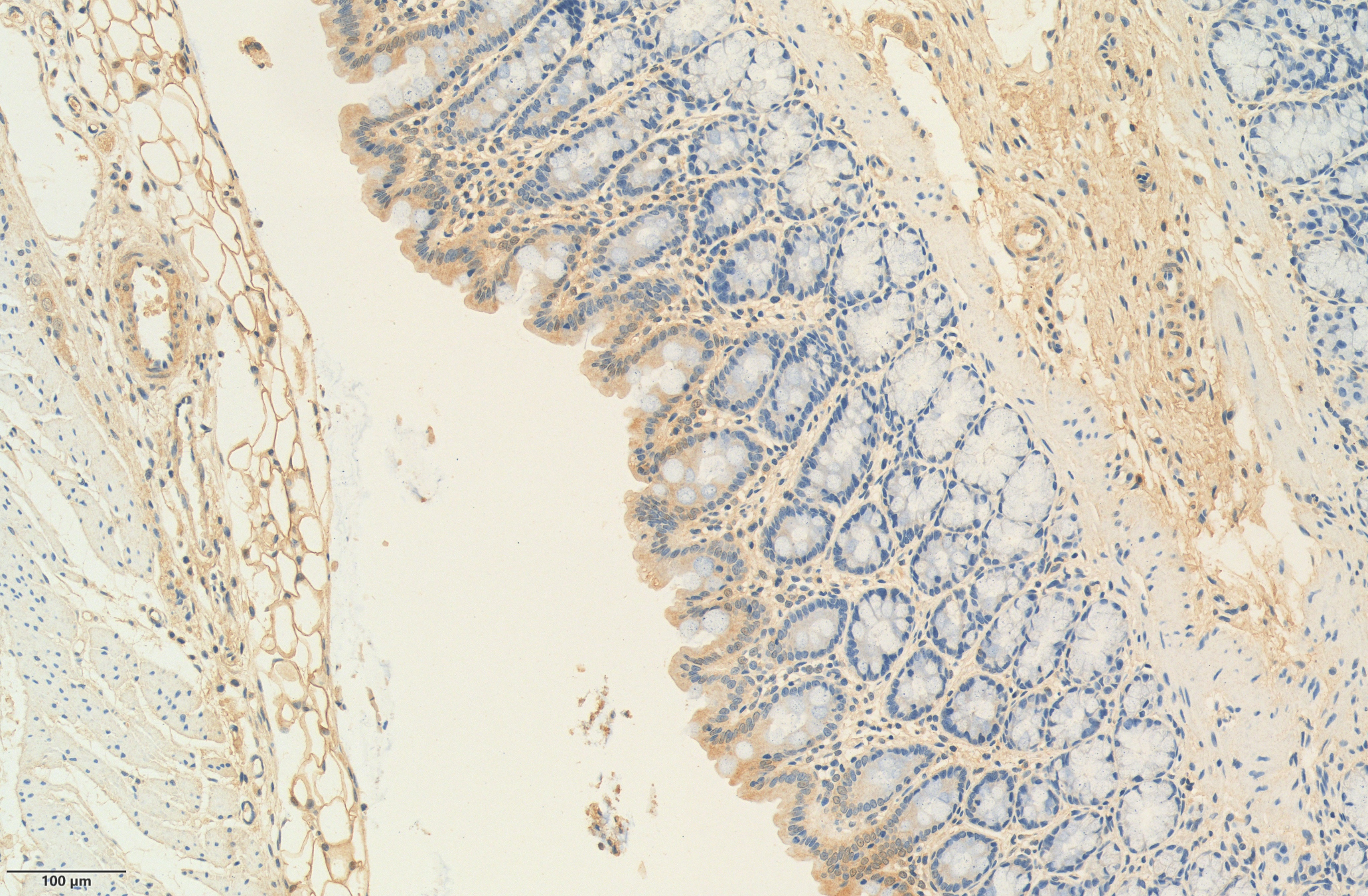

Supplement: S3 Fig — (ZIP) [file pone.0339296.s015.zip › CPT1A IHC_raw_image/Model-2-20x.tif]

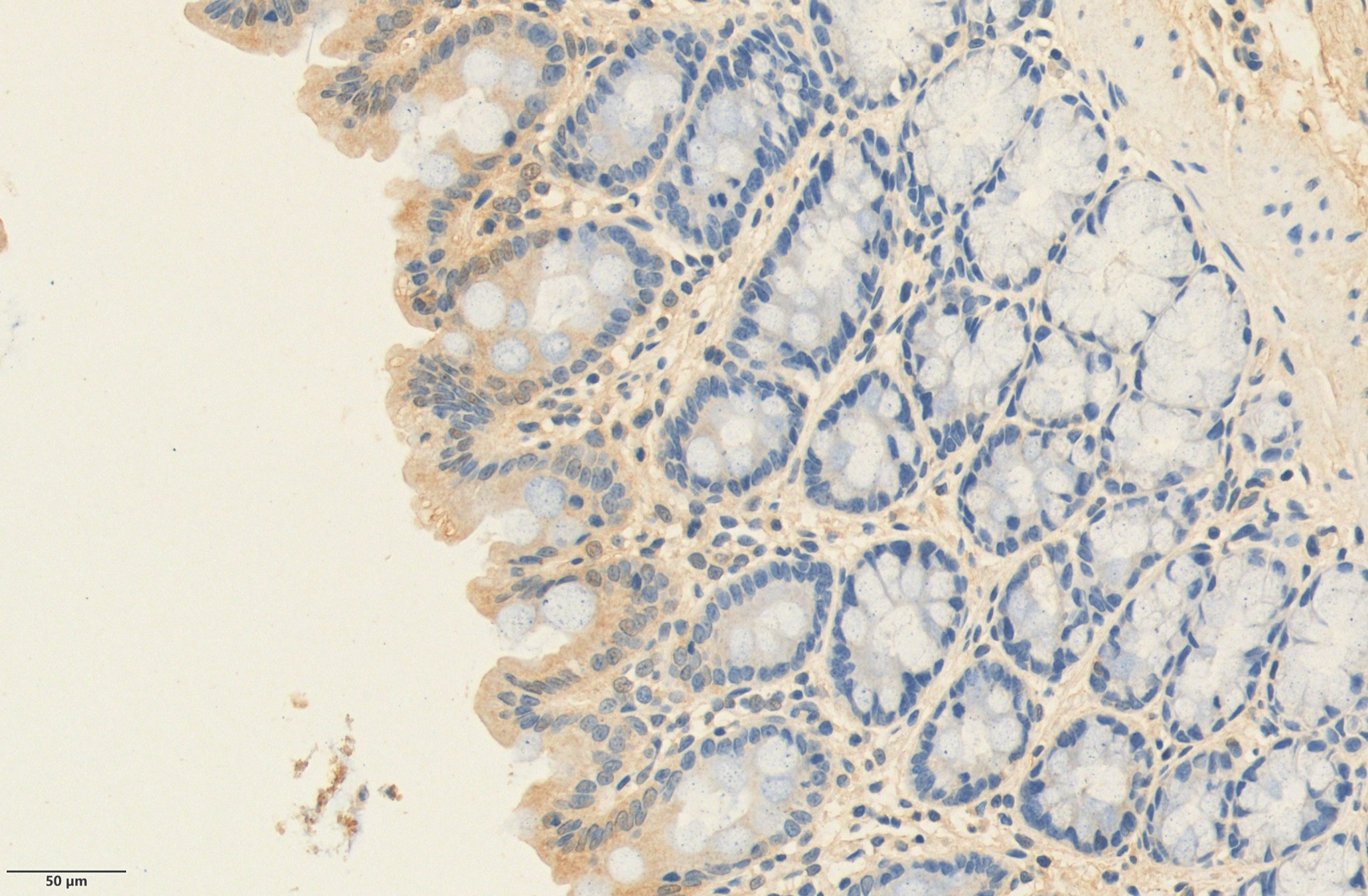

Supplement: S3 Fig — (ZIP) [file pone.0339296.s015.zip › CPT1A IHC_raw_image/Model-2-40x.tif]

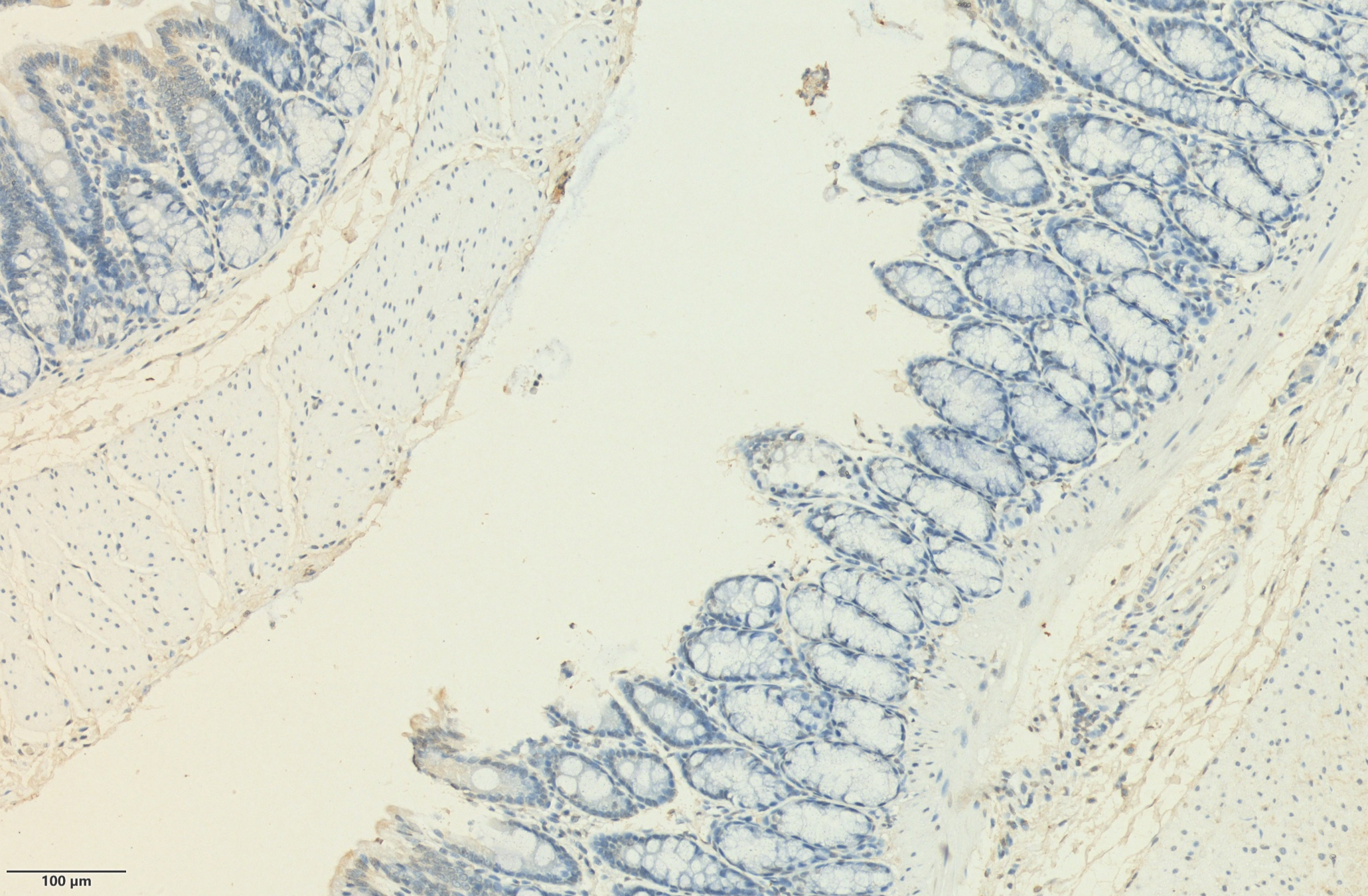

Supplement: S3 Fig — (ZIP) [file pone.0339296.s015.zip › CPT1A IHC_raw_image/Model-3-20x.tif]

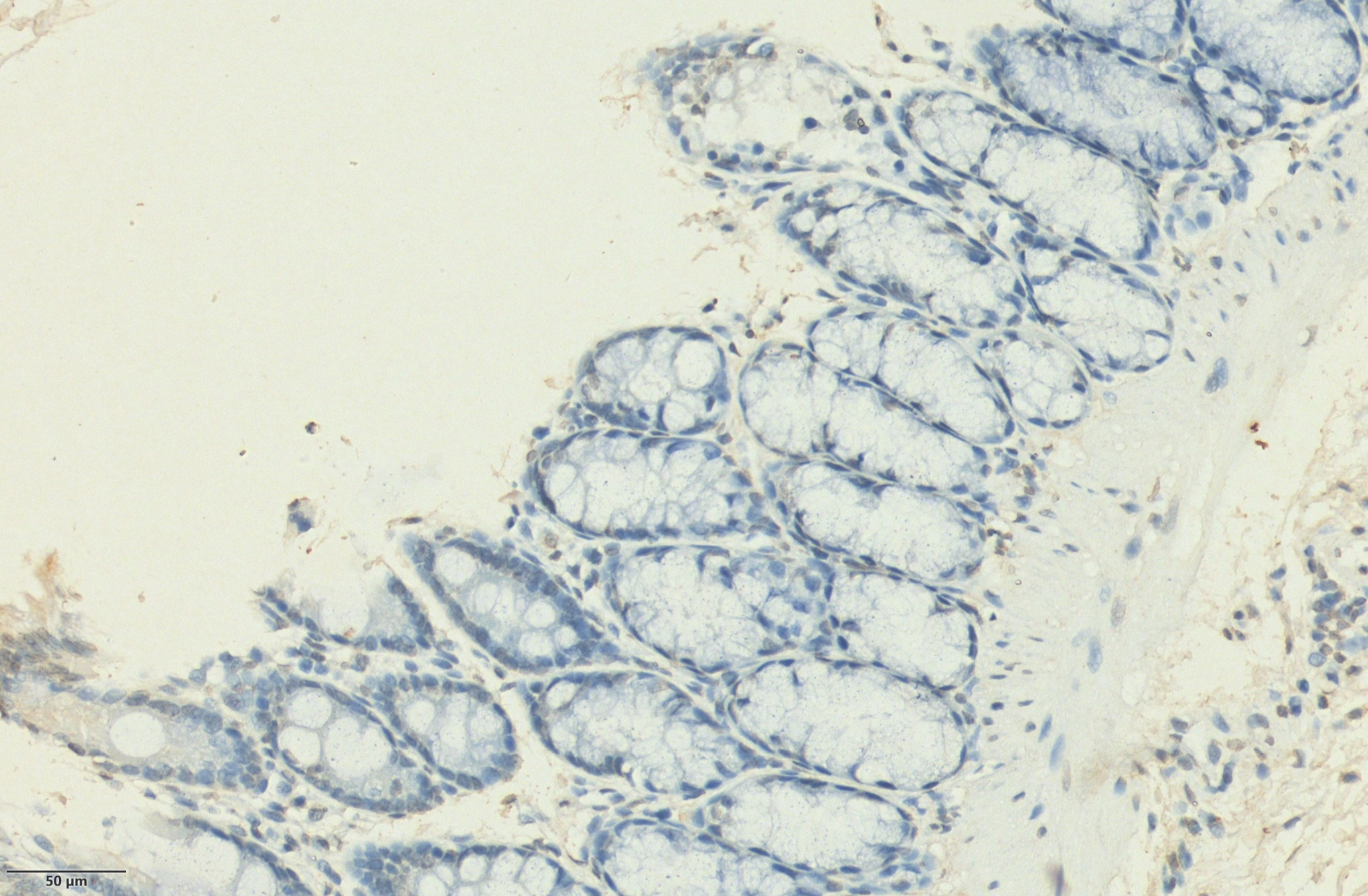

Supplement: S3 Fig — (ZIP) [file pone.0339296.s015.zip › CPT1A IHC_raw_image/Model-3-40x.tif]

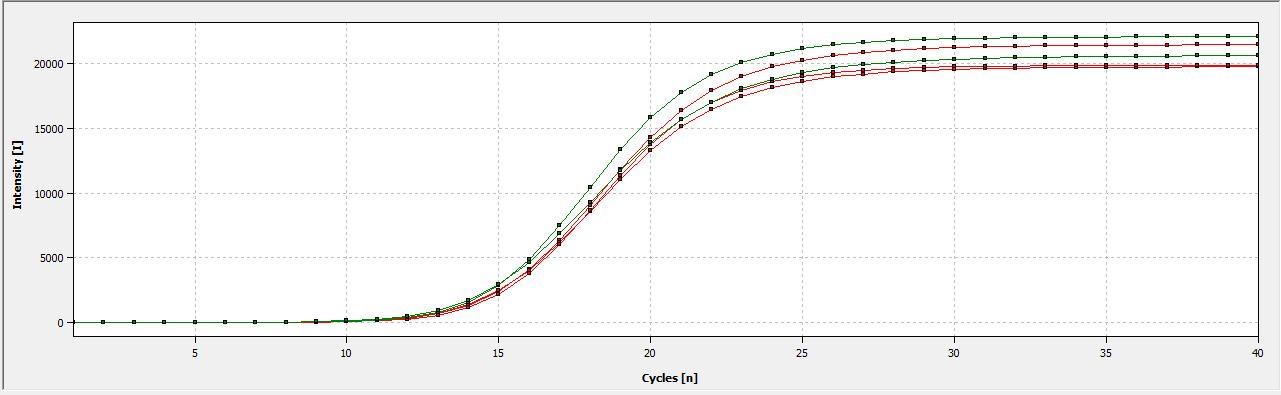

Supplement: S5 Fig — (ZIP) [file pone.0339296.s017.zip › qRT-PCR/actin/actin-2.JPG]

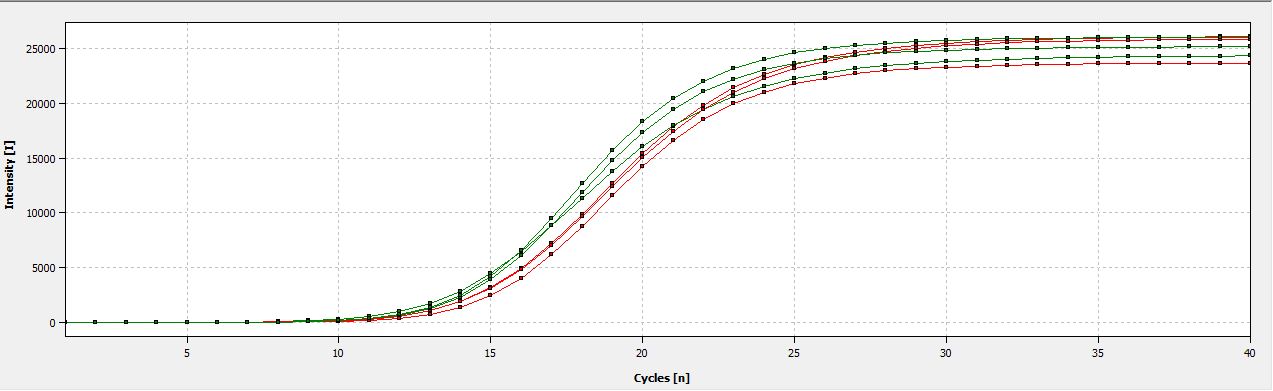

Supplement: S5 Fig — (ZIP) [file pone.0339296.s017.zip › qRT-PCR/actin/actin-3.JPG]

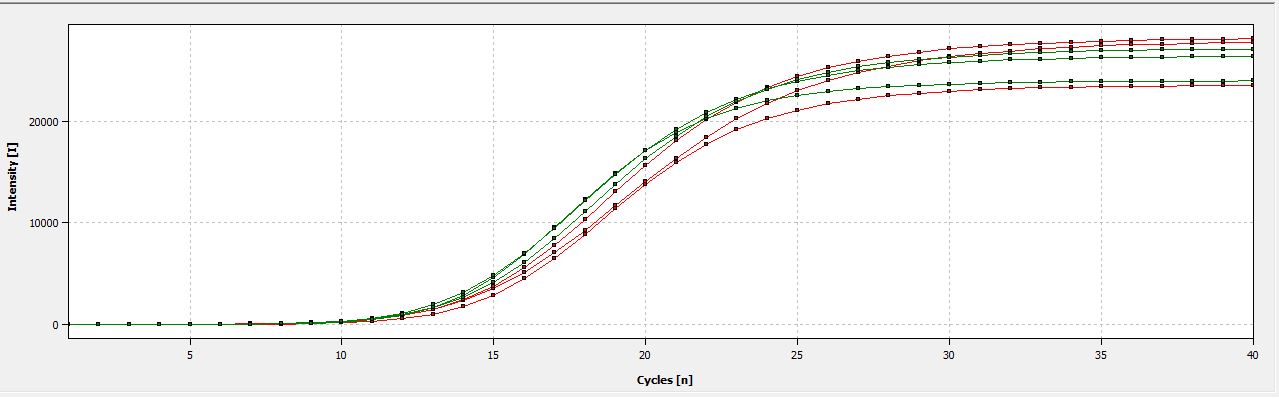

Supplement: S5 Fig — (ZIP) [file pone.0339296.s017.zip › qRT-PCR/actin/actin-4.JPG]

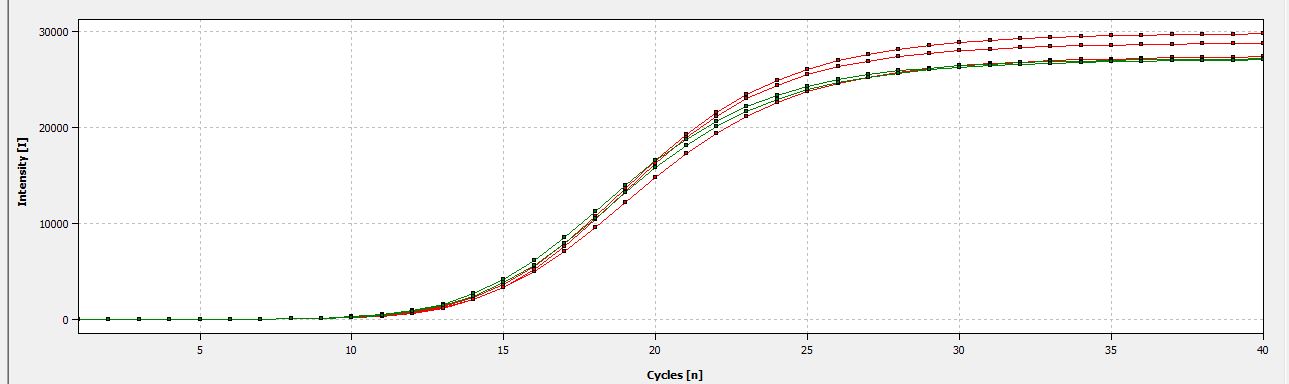

Supplement: S5 Fig — (ZIP) [file pone.0339296.s017.zip › qRT-PCR/actin/actin-5.JPG]

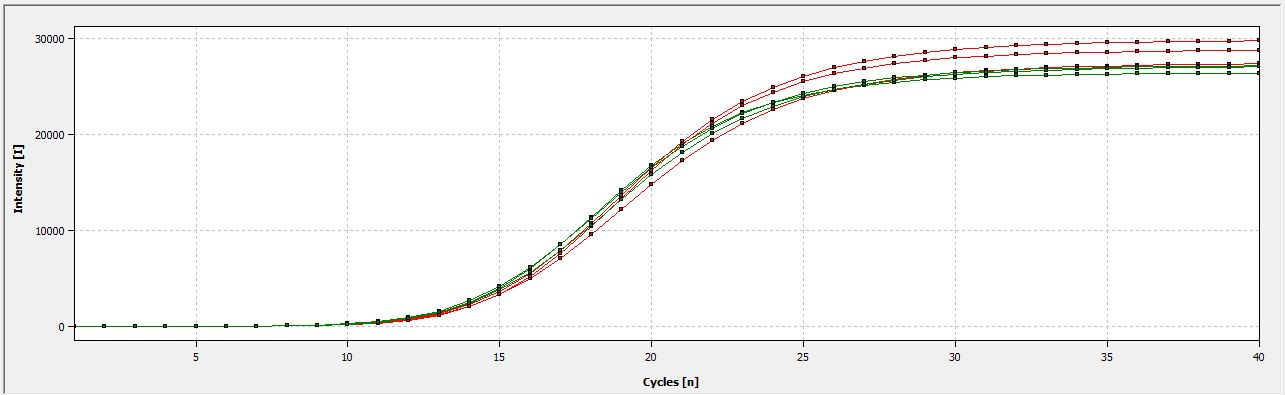

Supplement: S5 Fig — (ZIP) [file pone.0339296.s017.zip › qRT-PCR/actin/actin-6.JPG]

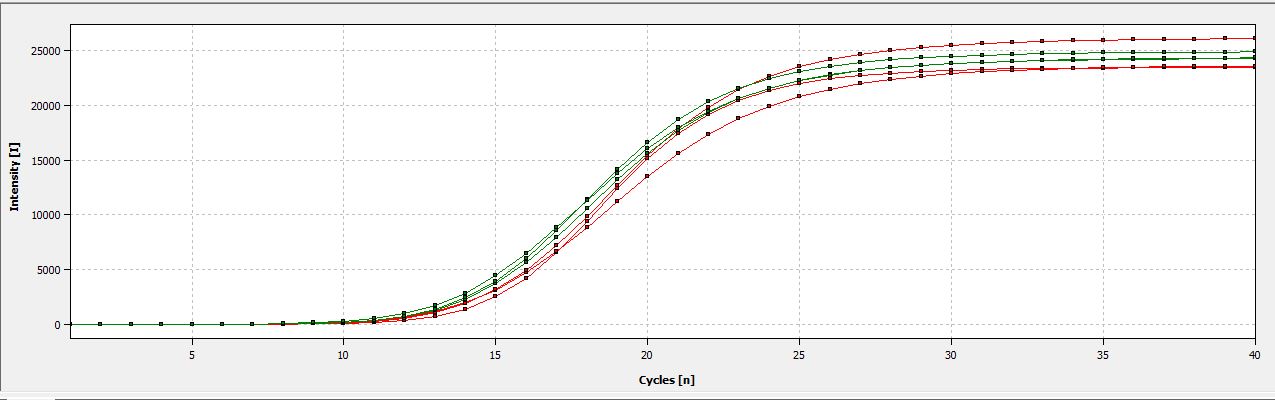

Supplement: S5 Fig — (ZIP) [file pone.0339296.s017.zip › qRT-PCR/actin/actin.JPG]

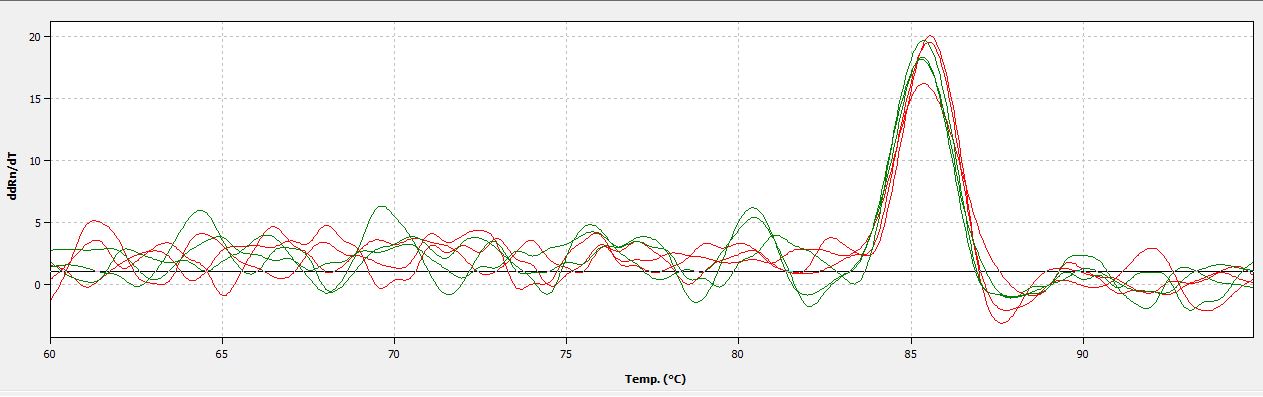

Supplement: S5 Fig — (ZIP) [file pone.0339296.s017.zip › qRT-PCR/actin/actinDissociation curve-2.JPG]

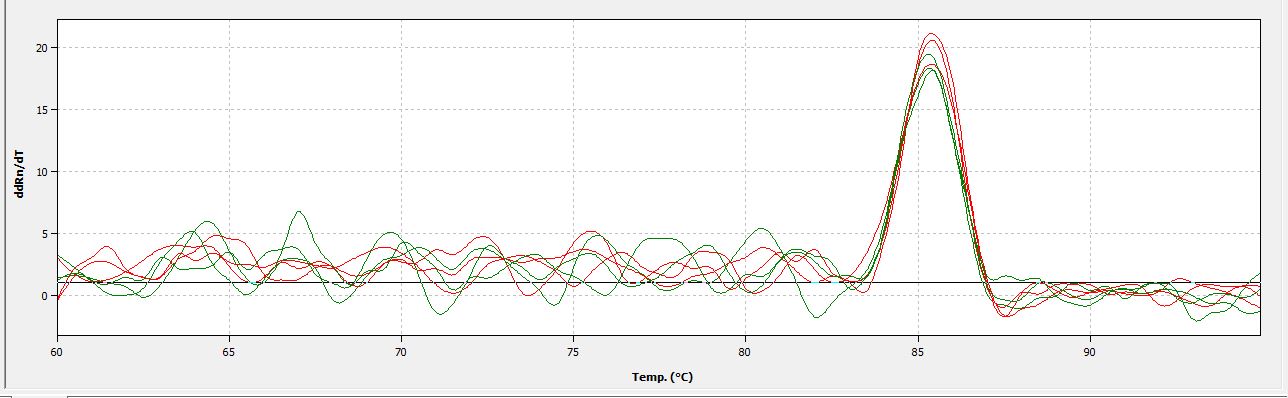

Supplement: S5 Fig — (ZIP) [file pone.0339296.s017.zip › qRT-PCR/actin/actinDissociation curve-3.JPG]

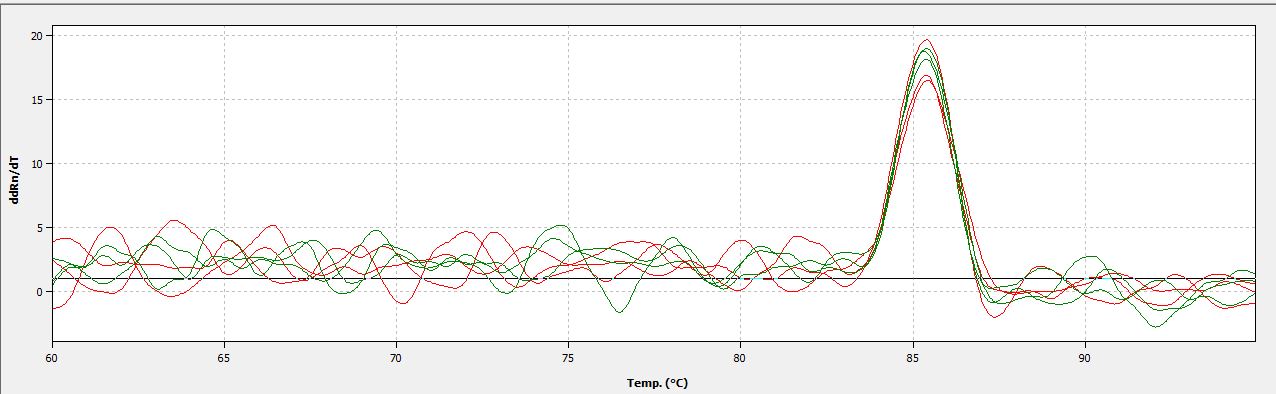

Supplement: S5 Fig — (ZIP) [file pone.0339296.s017.zip › qRT-PCR/actin/actinDissociation curve-4.JPG]

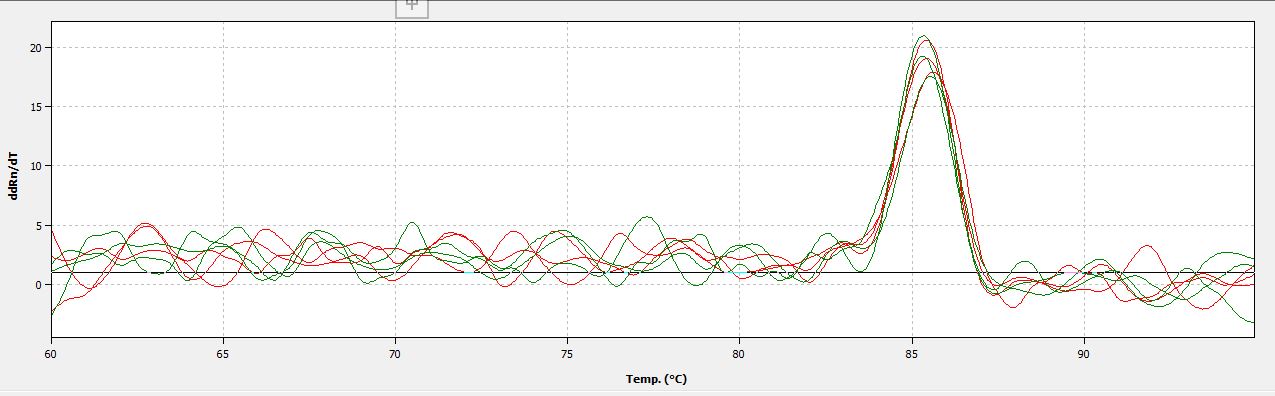

Supplement: S5 Fig — (ZIP) [file pone.0339296.s017.zip › qRT-PCR/actin/actinDissociation curve-5.JPG]

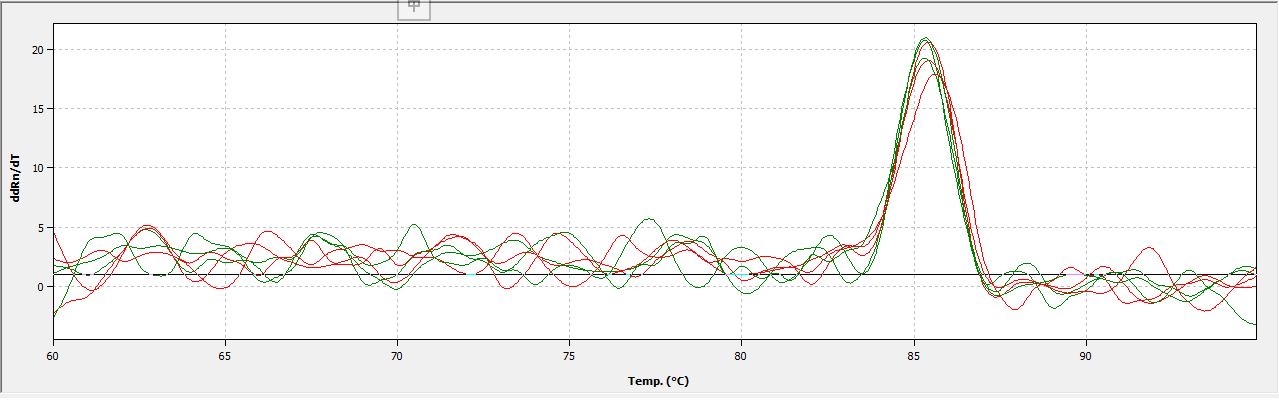

Supplement: S5 Fig — (ZIP) [file pone.0339296.s017.zip › qRT-PCR/actin/actinDissociation curve-6.JPG]

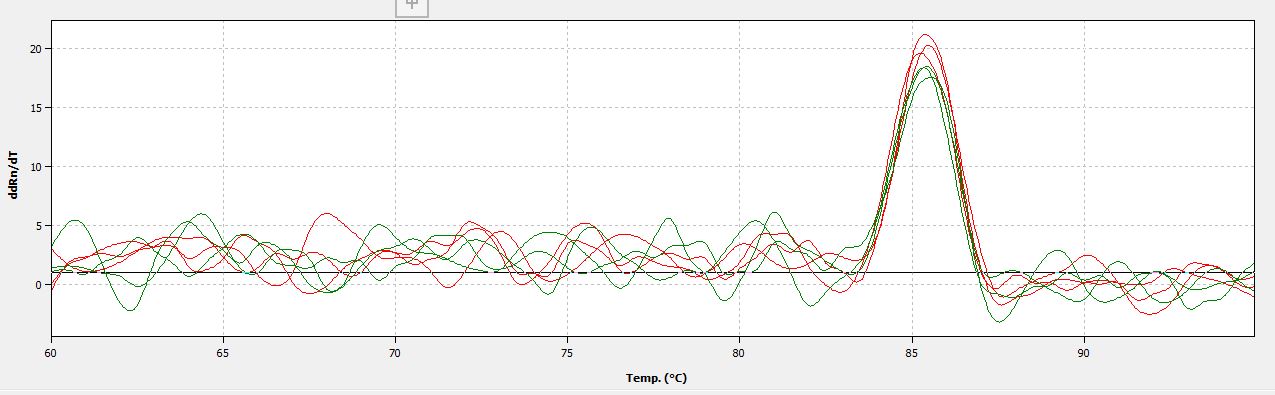

Supplement: S5 Fig — (ZIP) [file pone.0339296.s017.zip › qRT-PCR/actin/actinDissociation curve.JPG]

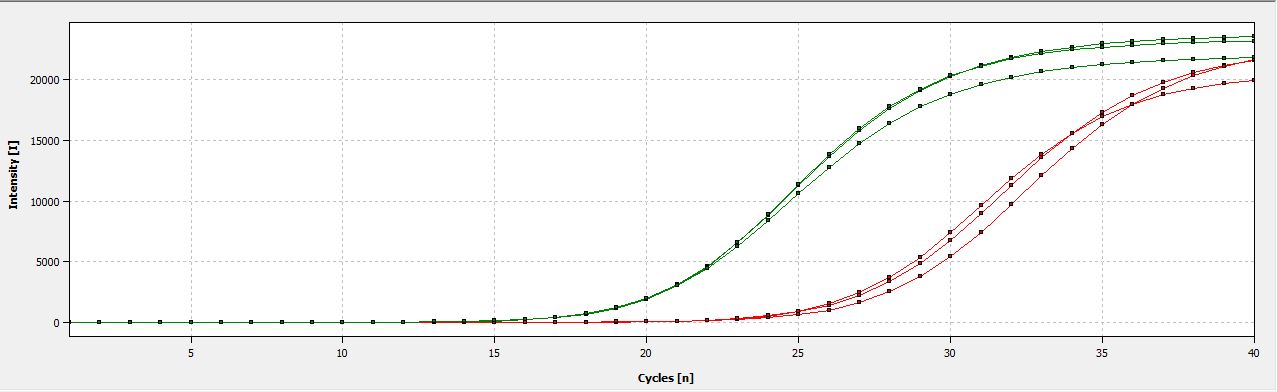

Supplement: S5 Fig — (ZIP) [file pone.0339296.s017.zip › qRT-PCR/cd55/cd55-1.JPG]

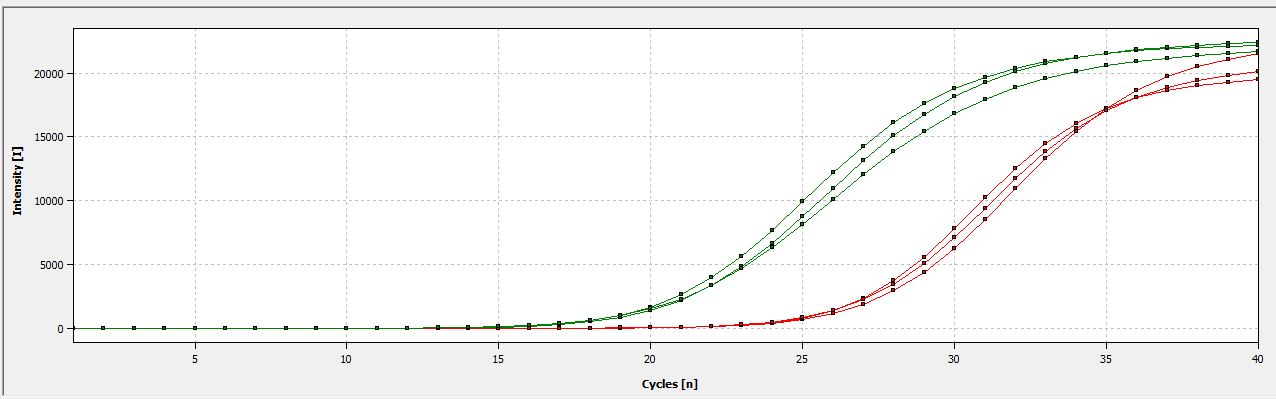

Supplement: S5 Fig — (ZIP) [file pone.0339296.s017.zip › qRT-PCR/cd55/cd55-2.JPG]

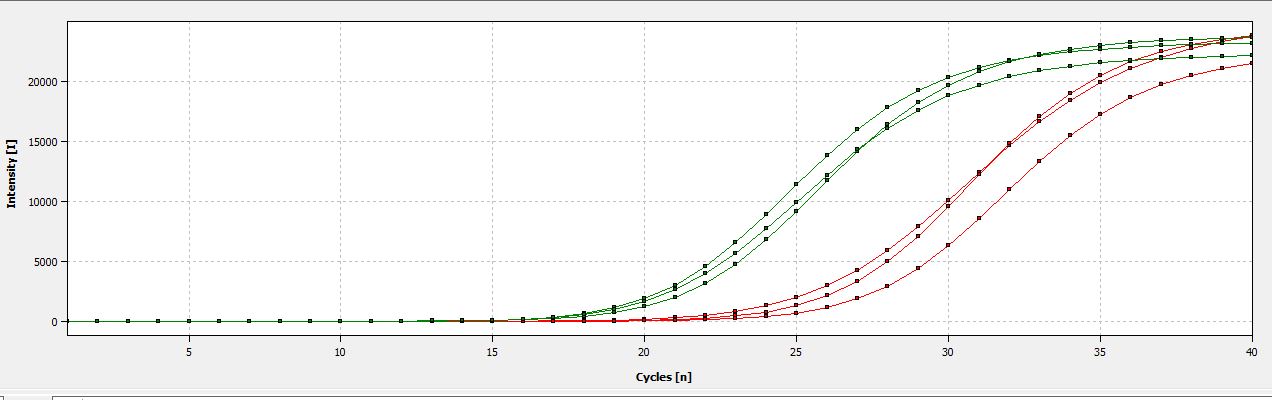

Supplement: S5 Fig — (ZIP) [file pone.0339296.s017.zip › qRT-PCR/cd55/cd55-3.JPG]

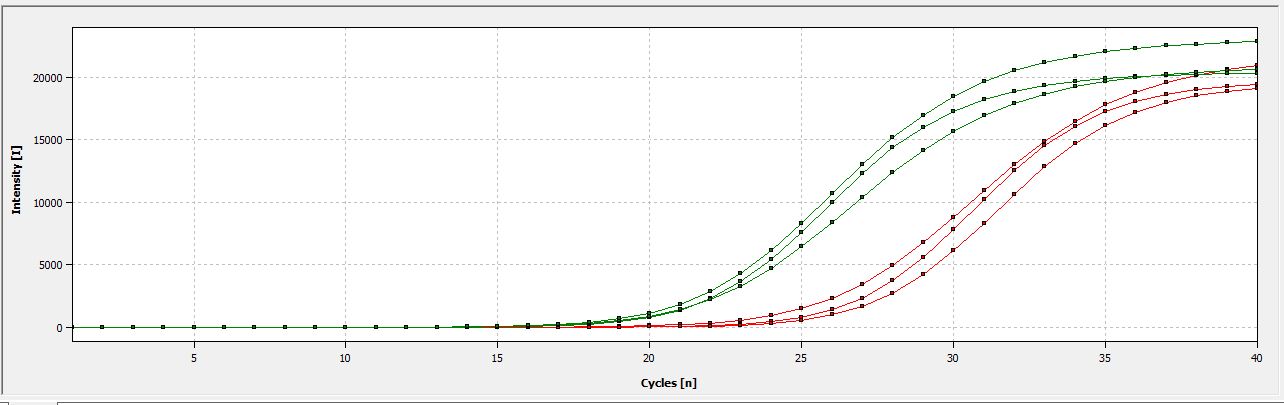

Supplement: S5 Fig — (ZIP) [file pone.0339296.s017.zip › qRT-PCR/cd55/cd55-4.JPG]

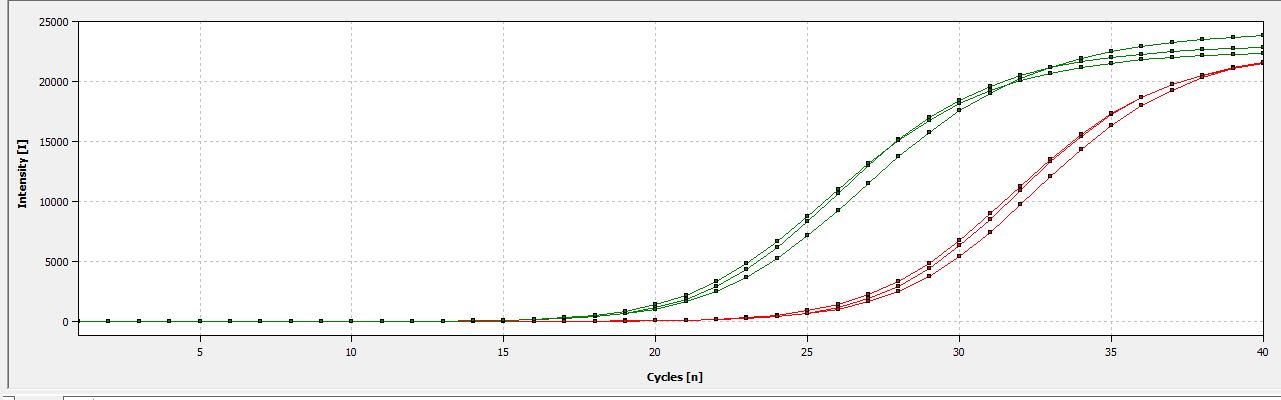

Supplement: S5 Fig — (ZIP) [file pone.0339296.s017.zip › qRT-PCR/cd55/cd55-5.JPG]

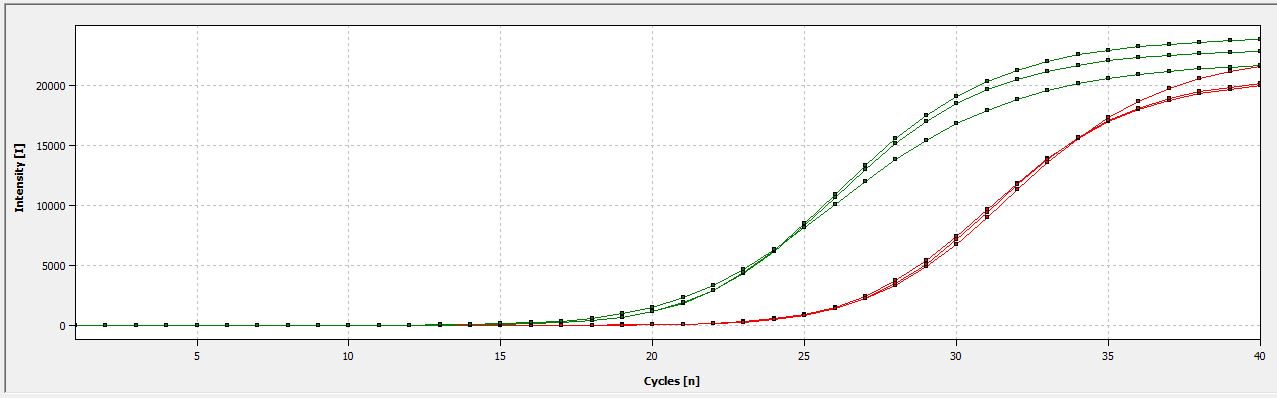

Supplement: S5 Fig — (ZIP) [file pone.0339296.s017.zip › qRT-PCR/cd55/cd55-6.JPG]

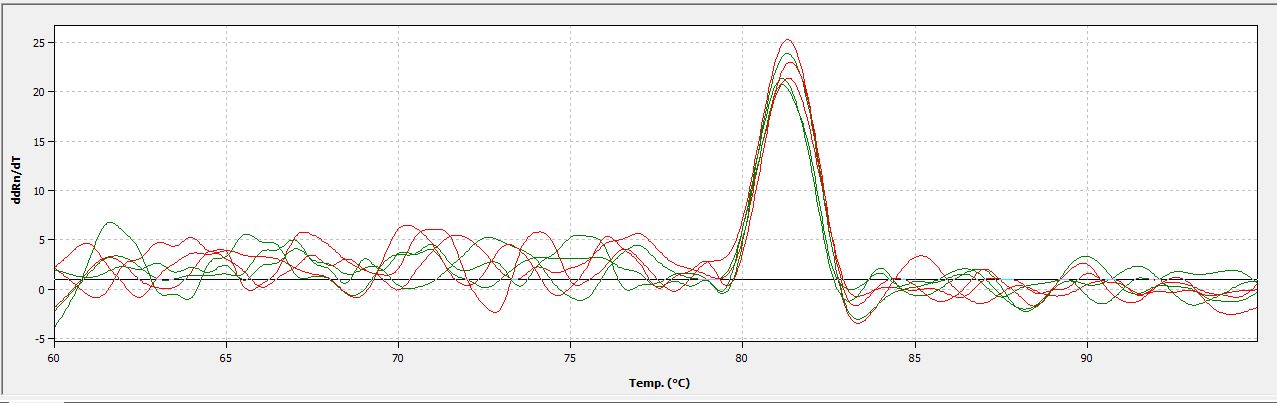

Supplement: S5 Fig — (ZIP) [file pone.0339296.s017.zip › qRT-PCR/cd55/CD55Dissociation curve-2.JPG]

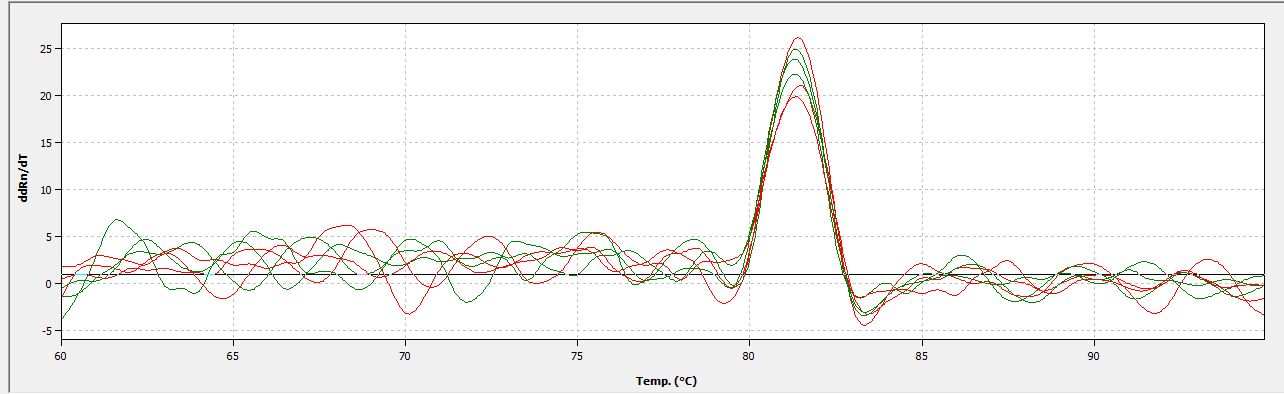

Supplement: S5 Fig — (ZIP) [file pone.0339296.s017.zip › qRT-PCR/cd55/CD55Dissociation curve-3.JPG]

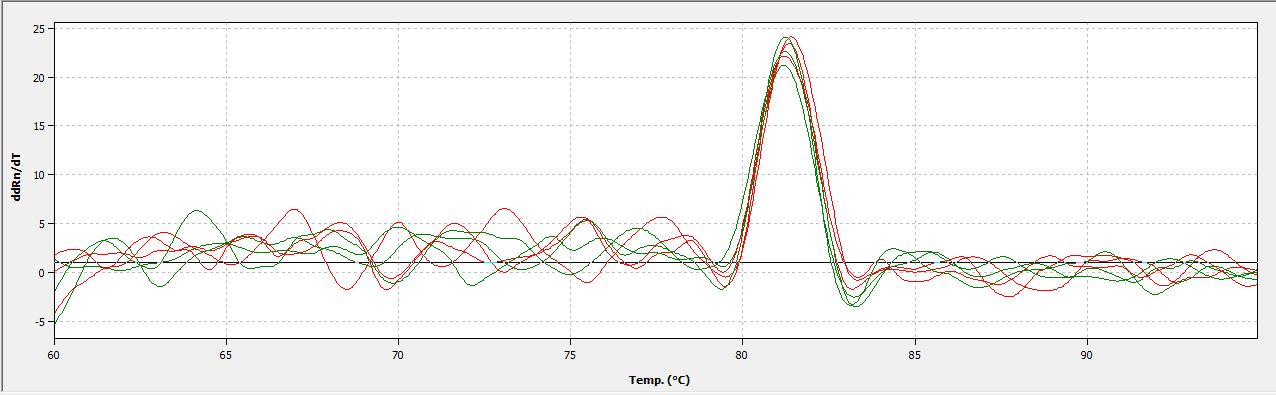

Supplement: S5 Fig — (ZIP) [file pone.0339296.s017.zip › qRT-PCR/cd55/CD55Dissociation curve-4.JPG]

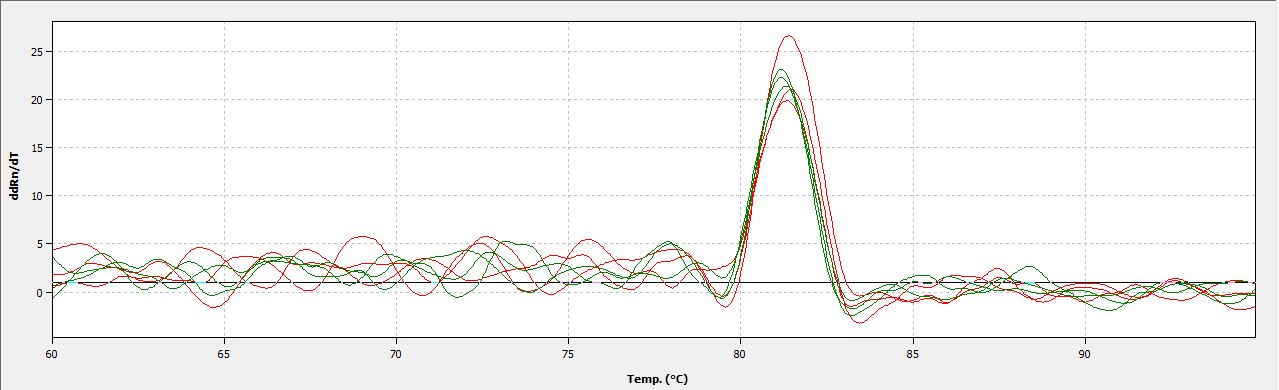

Supplement: S5 Fig — (ZIP) [file pone.0339296.s017.zip › qRT-PCR/cd55/CD55Dissociation curve-5.JPG]

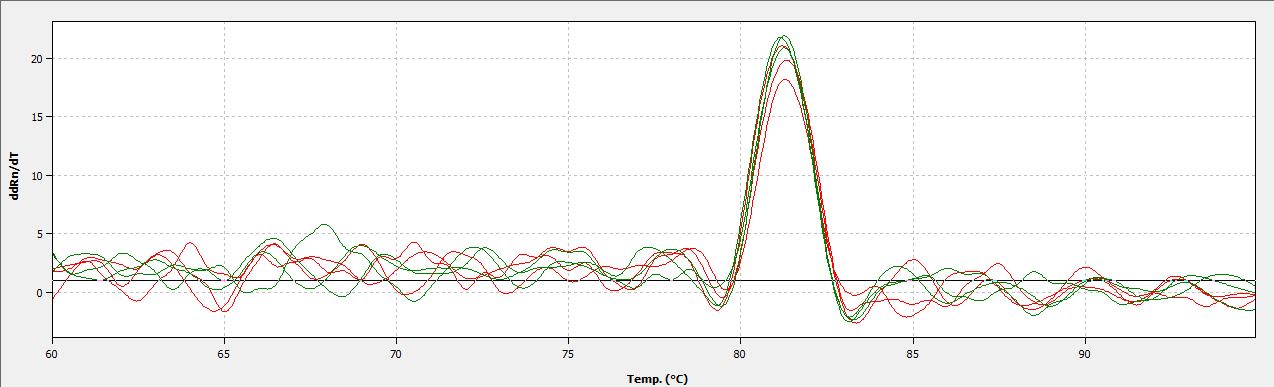

Supplement: S5 Fig — (ZIP) [file pone.0339296.s017.zip › qRT-PCR/cd55/CD55Dissociation curve-6.JPG]

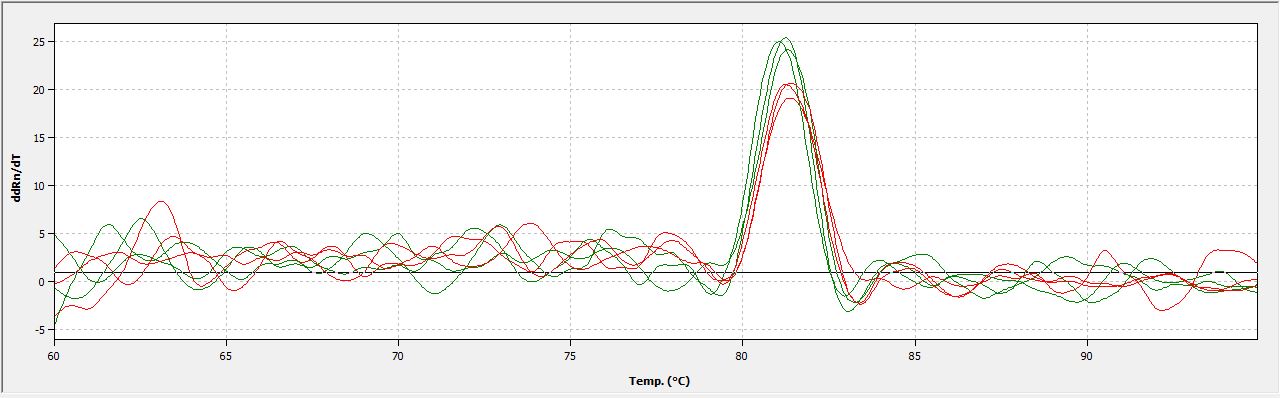

Supplement: S5 Fig — (ZIP) [file pone.0339296.s017.zip › qRT-PCR/cd55/CD55Dissociation curve.JPG]

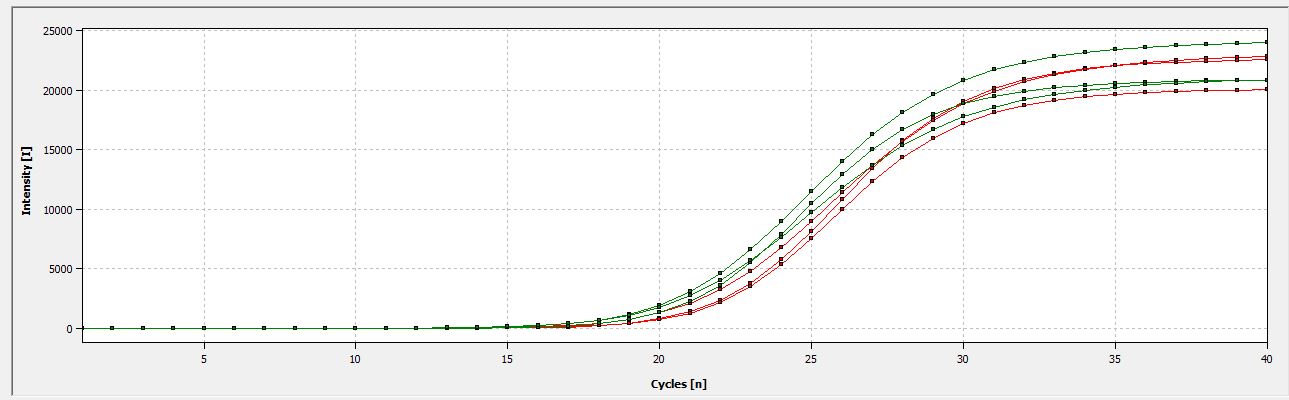

Supplement: S5 Fig — (ZIP) [file pone.0339296.s017.zip › qRT-PCR/cpta1/cpt1a-1.JPG]

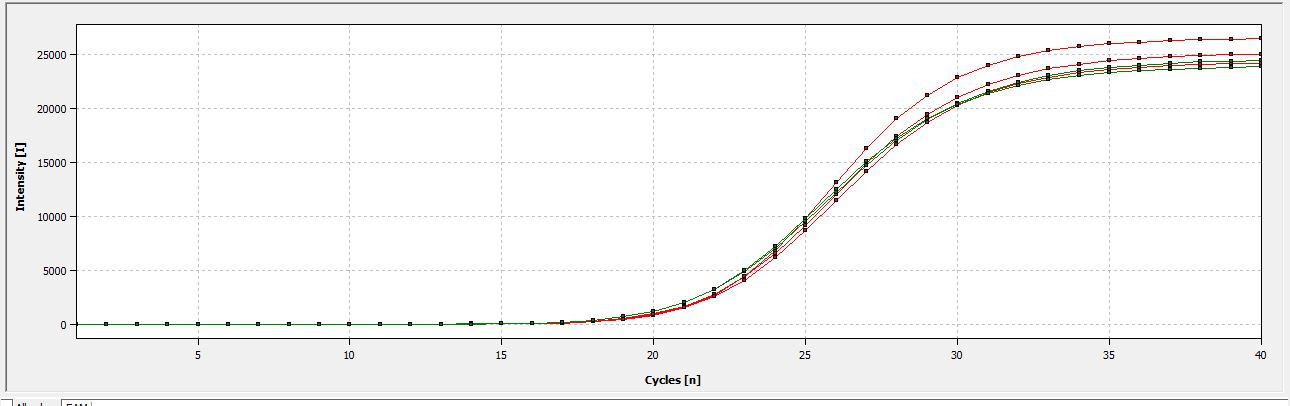

Supplement: S5 Fig — (ZIP) [file pone.0339296.s017.zip › qRT-PCR/cpta1/cpt1a-2.JPG]

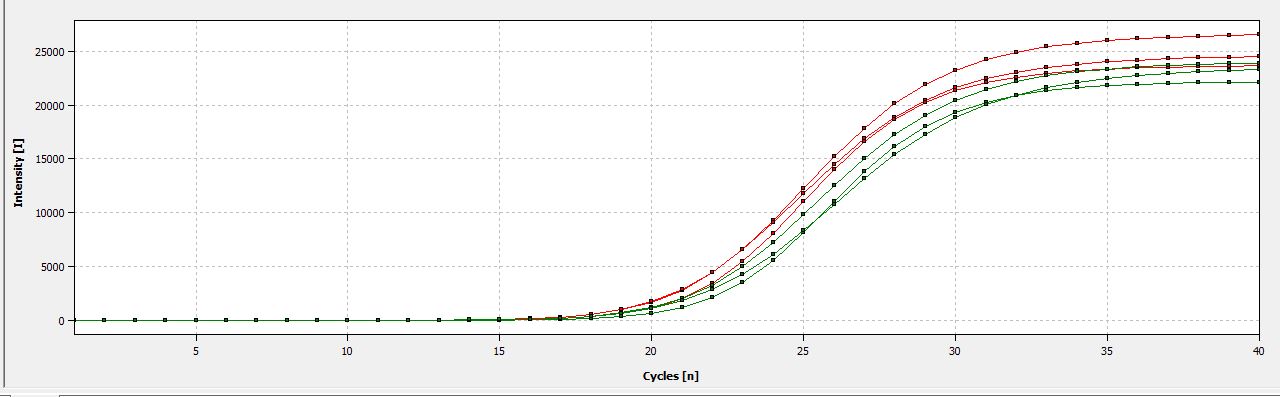

Supplement: S5 Fig — (ZIP) [file pone.0339296.s017.zip › qRT-PCR/cpta1/cpt1a-3.JPG]

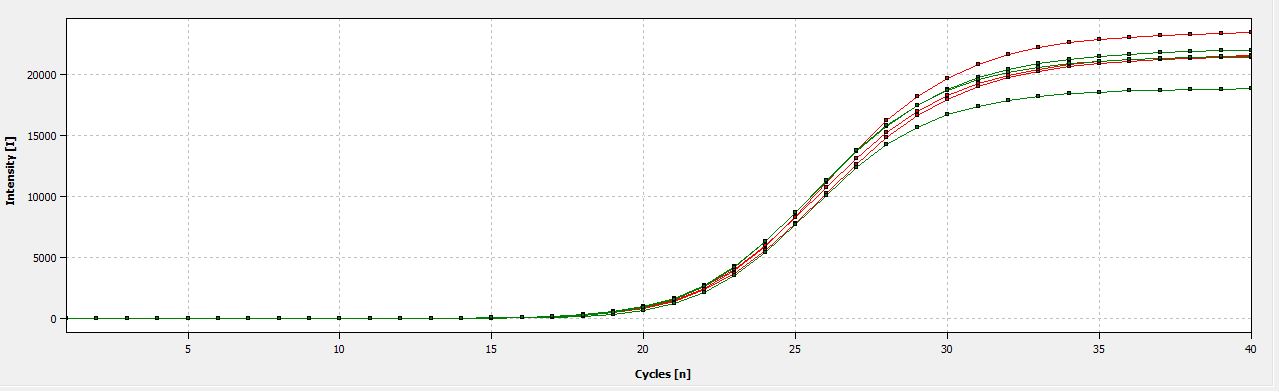

Supplement: S5 Fig — (ZIP) [file pone.0339296.s017.zip › qRT-PCR/cpta1/cpt1a-4.JPG]

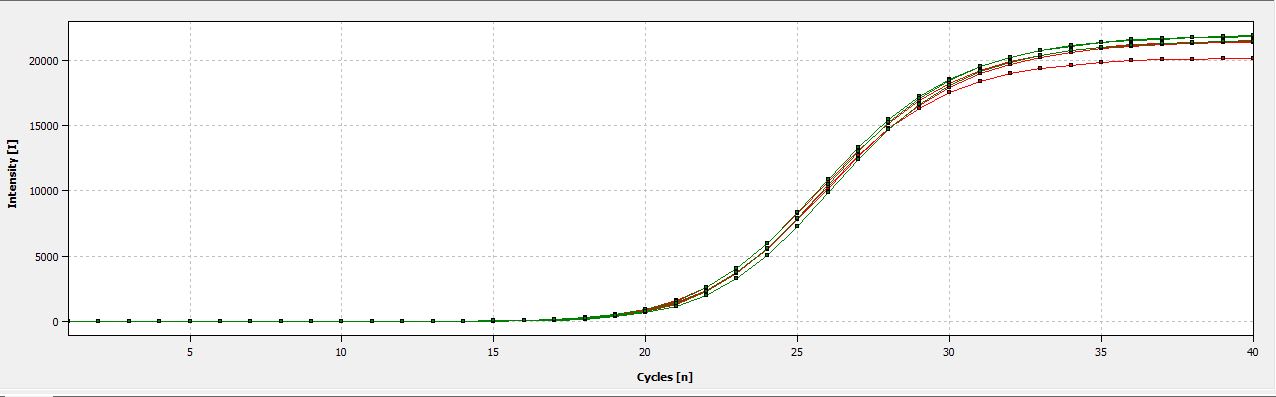

Supplement: S5 Fig — (ZIP) [file pone.0339296.s017.zip › qRT-PCR/cpta1/cpt1a-5.JPG]

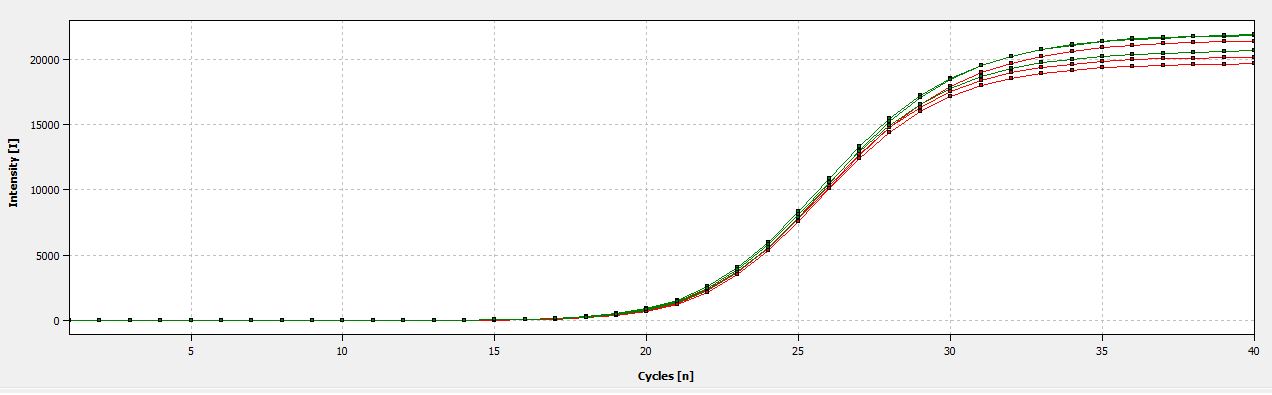

Supplement: S5 Fig — (ZIP) [file pone.0339296.s017.zip › qRT-PCR/cpta1/cpt1a-6.JPG]

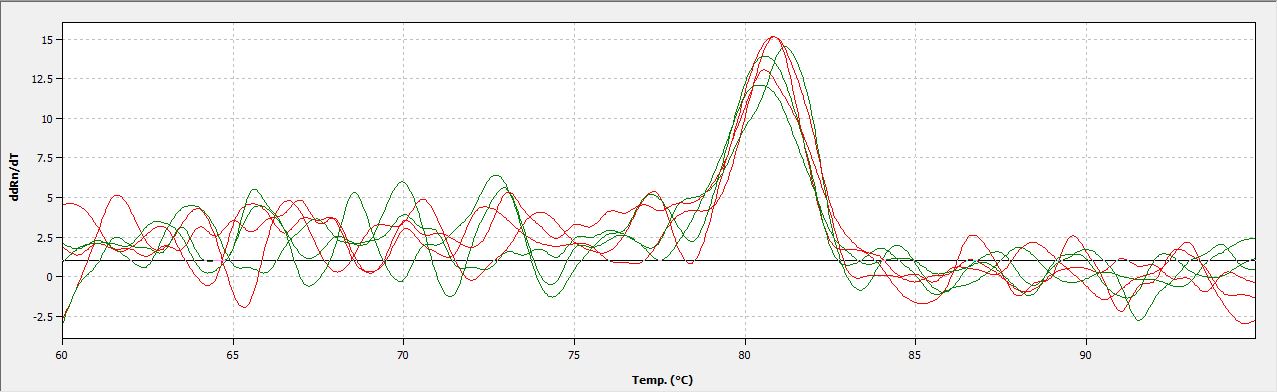

Supplement: S5 Fig — (ZIP) [file pone.0339296.s017.zip › qRT-PCR/cpta1/cpt1aDissociation curve-1.JPG]

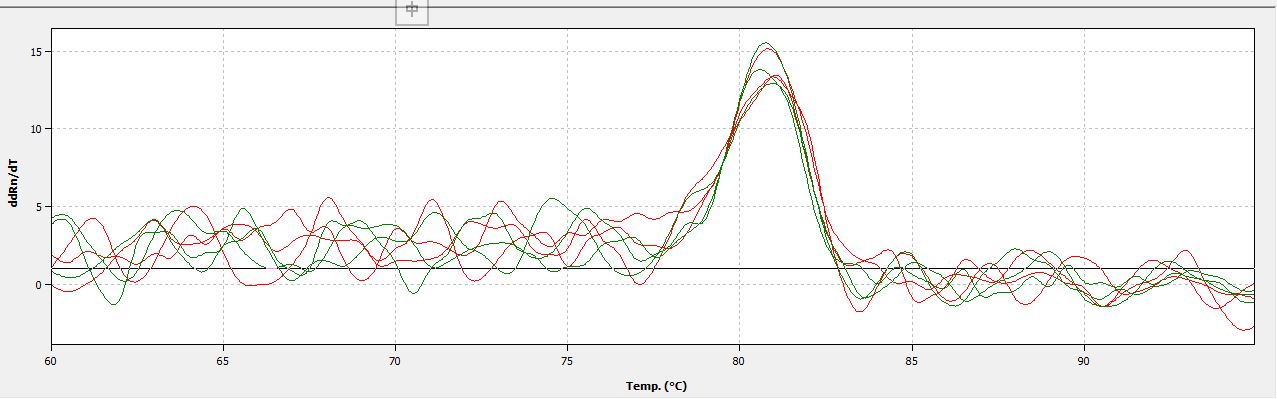

Supplement: S5 Fig — (ZIP) [file pone.0339296.s017.zip › qRT-PCR/cpta1/cpt1aDissociation curve-2.JPG]

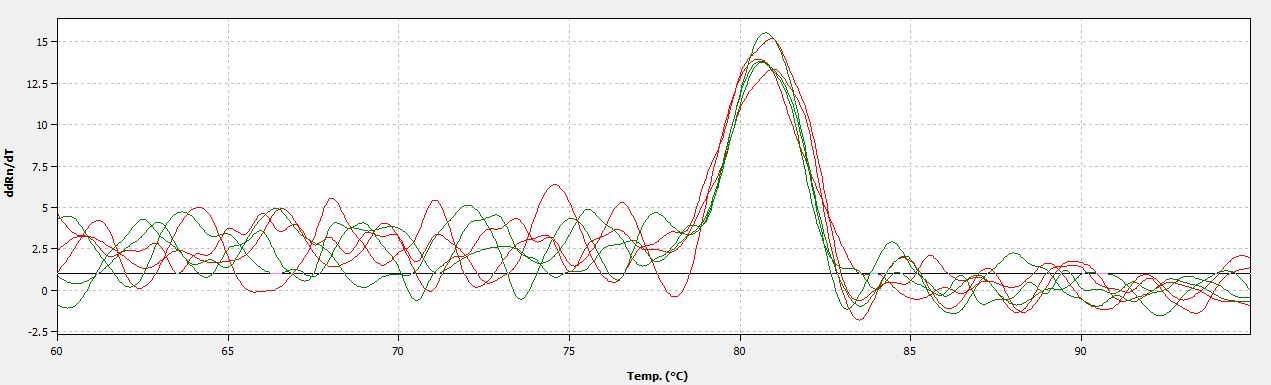

Supplement: S5 Fig — (ZIP) [file pone.0339296.s017.zip › qRT-PCR/cpta1/cpt1aDissociation curve-3.JPG]

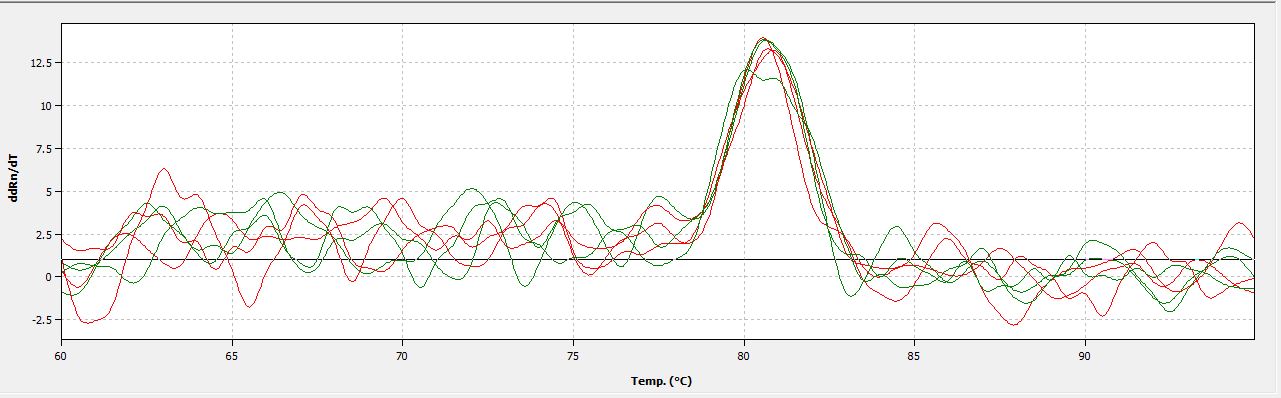

Supplement: S5 Fig — (ZIP) [file pone.0339296.s017.zip › qRT-PCR/cpta1/cpt1aDissociation curve-4.JPG]

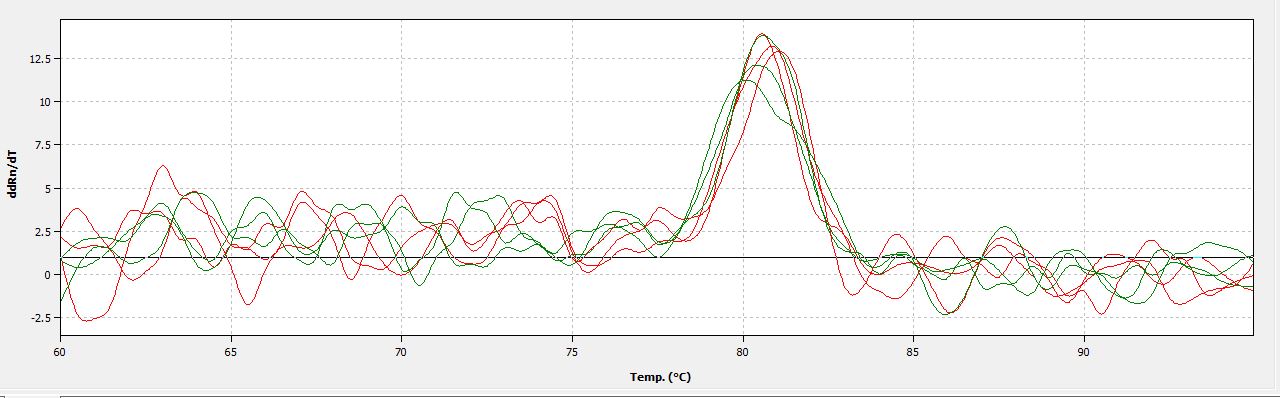

Supplement: S5 Fig — (ZIP) [file pone.0339296.s017.zip › qRT-PCR/cpta1/cpt1aDissociation curve-5.JPG]

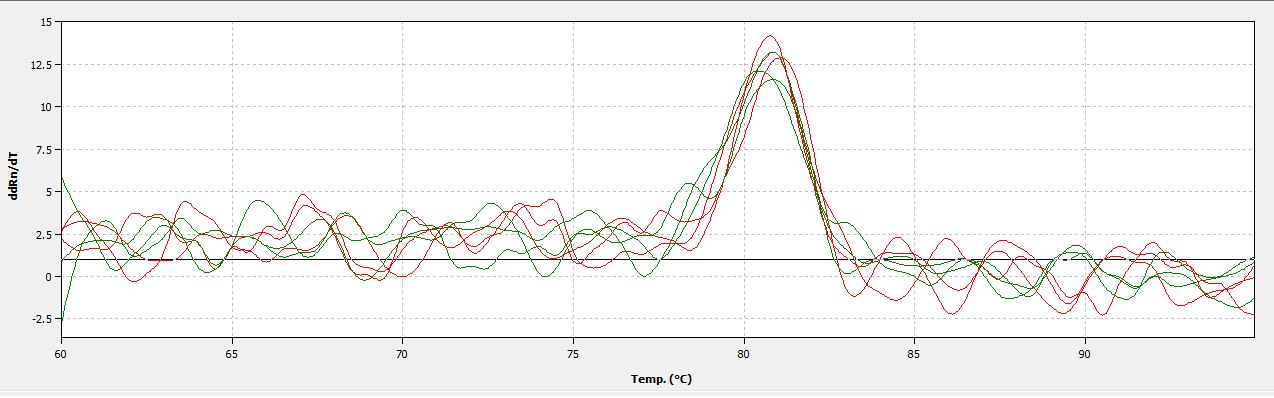

Supplement: S5 Fig — (ZIP) [file pone.0339296.s017.zip › qRT-PCR/cpta1/cpt1aDissociation curve-6.JPG]
