# Supplementary material for: Identification of hypoxia- and mitophagy-related diagnostic biomarkers for ulcerative colitis based on bioinformatic analysis and machine learning
Source: PLoS One. 2026 Jan 21;21(1):e0339296. doi: 10.1371/journal.pone.0339296 (PMC12822963; doi:10.1371/journal.pone.0339296)
Supplement: S4 Fig — (PDF) [file pone.0339296.s016.pdf]

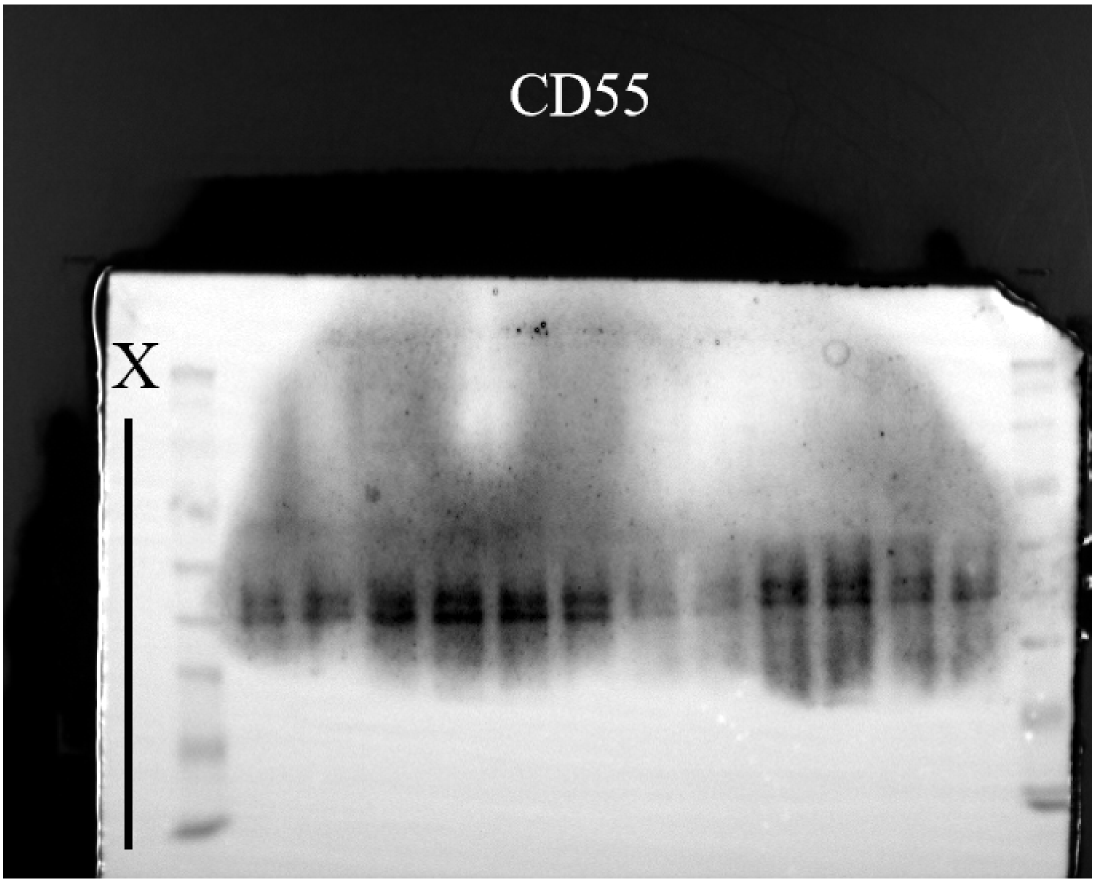

Blue 250kDa
Blue 150kDa
Blue 100kDa
Red 70kDa
Blue 50kDa
Blue 40kDa
Blue 35kDa
Red 25kDa
Blue 20kDa

Unsampled lane

Blue 250kDa
Blue 150kDa
Blue 100kDa
Red 70kDa
Blue 50kDa
Blue 40kDa
Blue 35kDa
Red 25kDa
Blue 20kDa

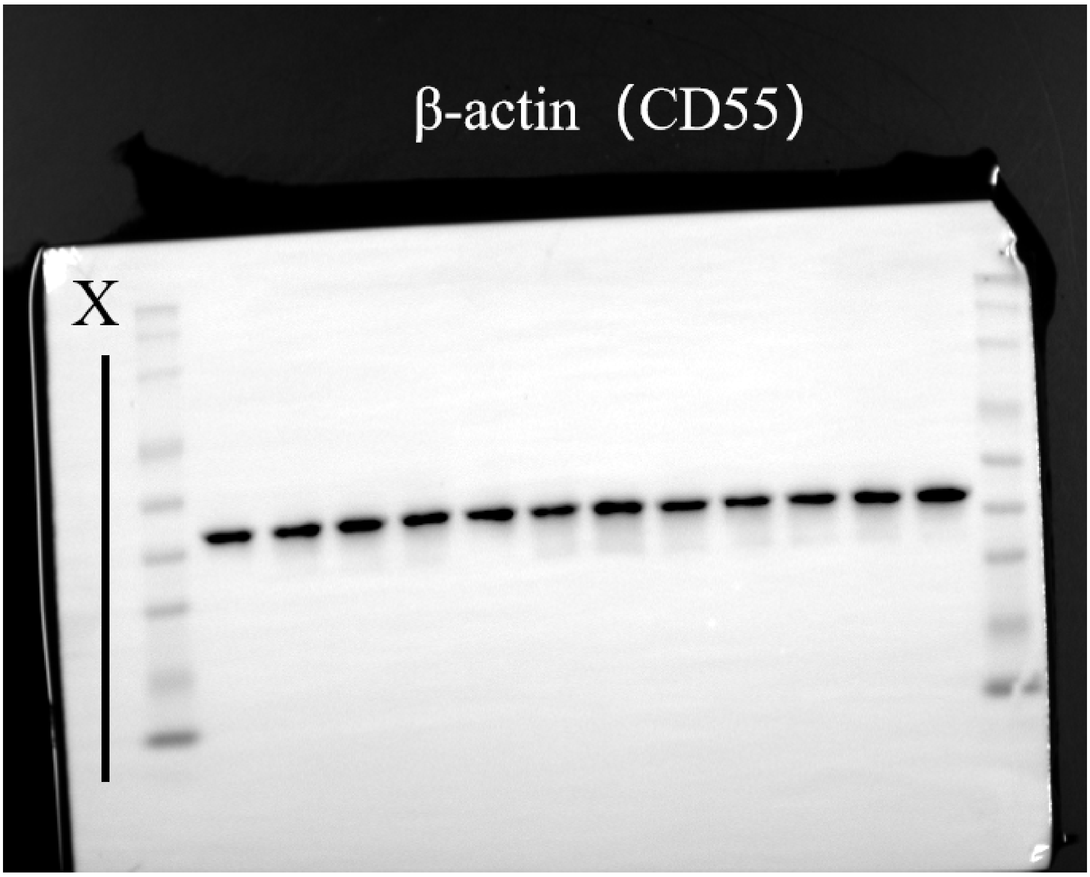

Unsampled lane

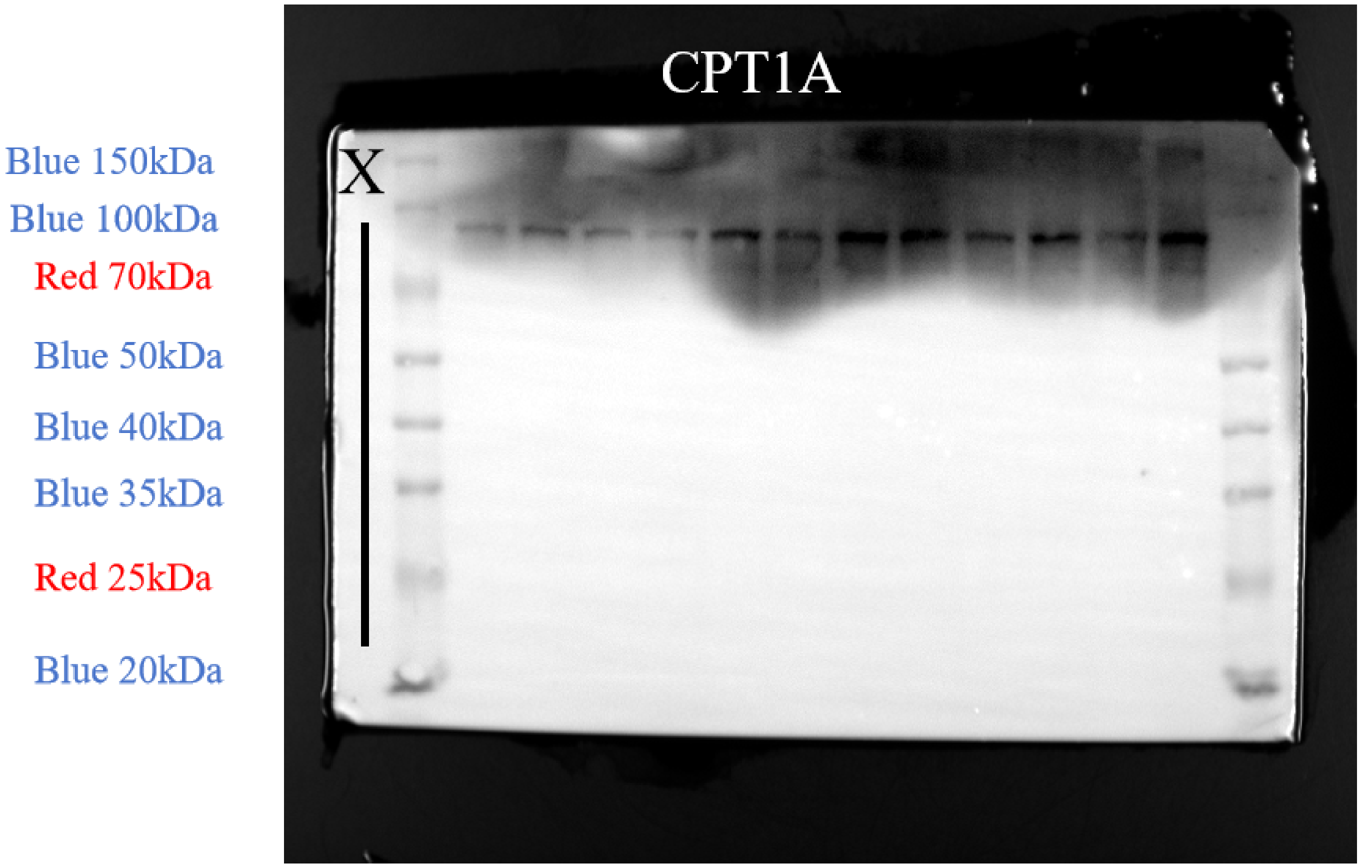

Unsampled lane

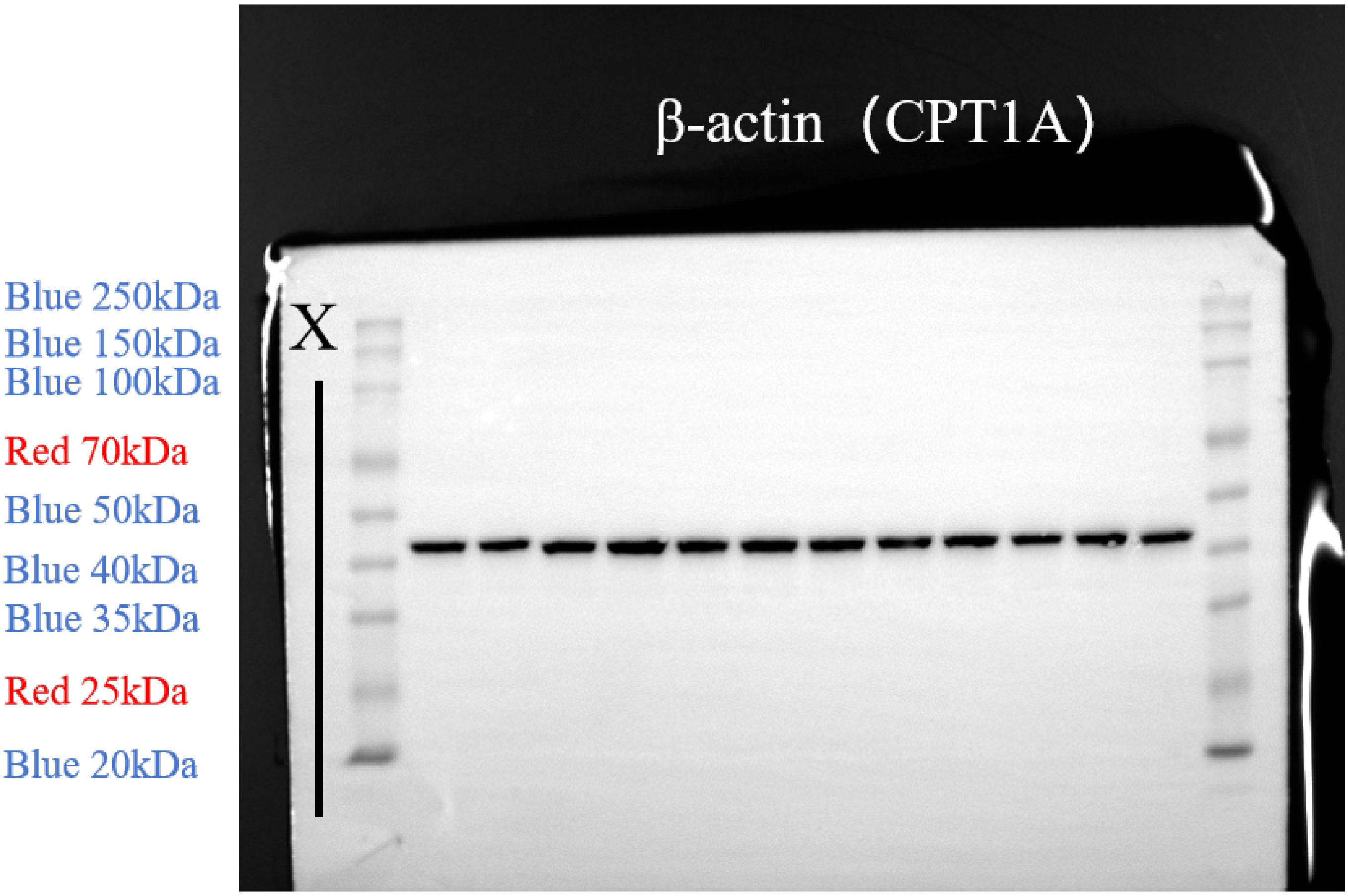

Unsampled lane

Total of sample raw image

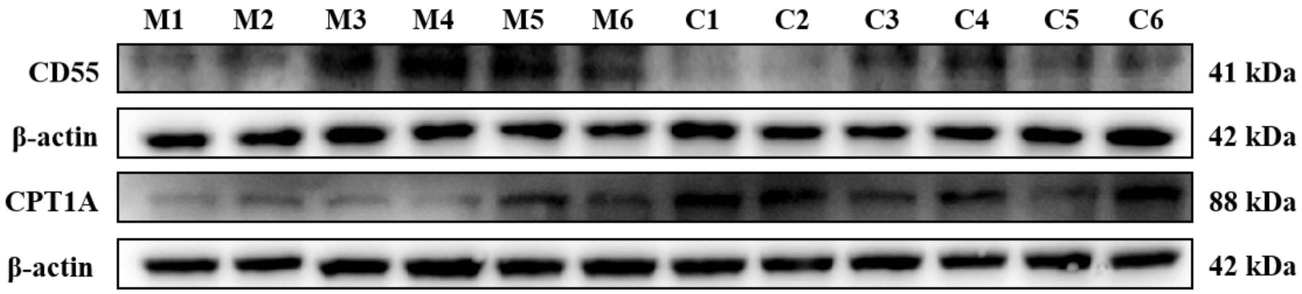

Marker of raw image

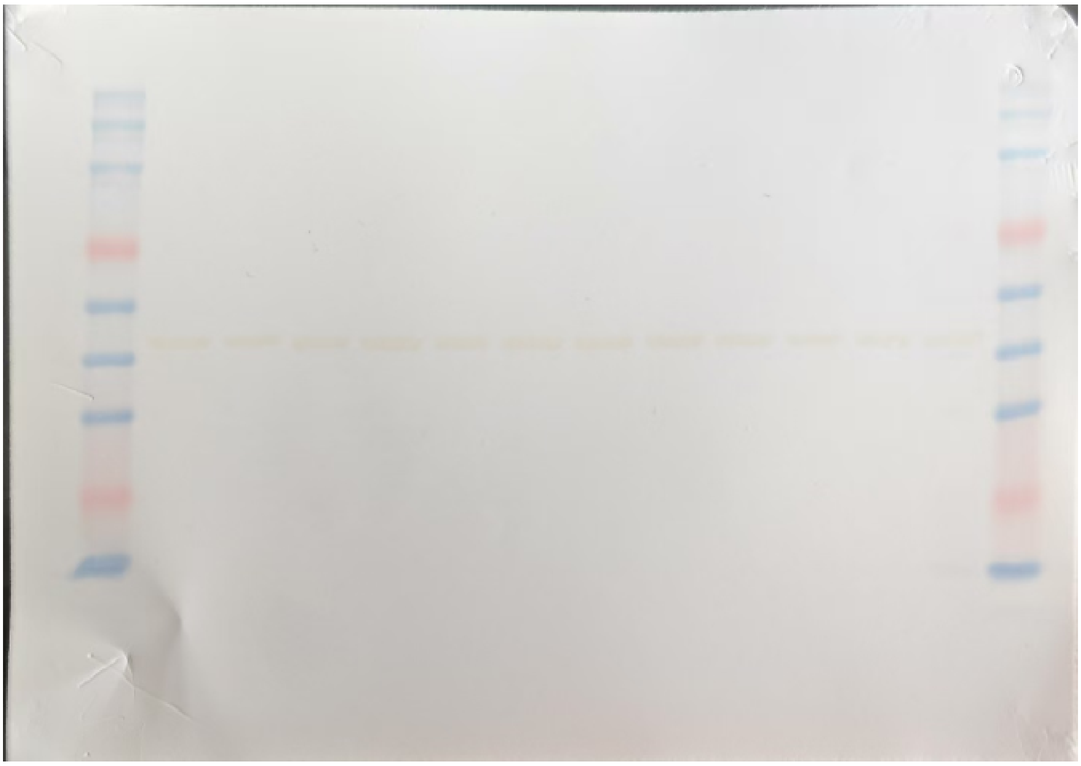

Blue 250kDa
Blue 150kDa
Blue 100kDa
Red 70kDa
Blue 50kDa
Blue 40kDa
Blue 35kDa
Red 25kDa
Blue 20kDa
